# Reduced white matter maturation in the central auditory system of children living with HIV

**DOI:** 10.3389/fnimg.2024.1341607

**Published:** 2024-03-06

**Authors:** Joanah Madzime, Marcin Jankiewicz, Ernesta M. Meintjes, Peter Torre, Barbara Laughton, Andre J. W. van der Kouwe, Martha Holmes

**Affiliations:** ^1^Biomedical Engineering Research Centre, Division of Biomedical Engineering, Department of Human Biology, University of Cape Town, Cape Town, South Africa; ^2^Neuroscience Institute, University of Cape Town, Cape Town, South Africa; ^3^Cape Universities Body Imaging Centre, University of Cape Town, Cape Town, South Africa; ^4^School of Speech, Language, and Hearing Sciences, College of Health and Human Services, San Diego, CA, United States; ^5^Family Centre for Research with Ubuntu, Department of Paediatrics and Child Health, Stellenbosch University, Stellenbosch, South Africa; ^6^A.A. Martinos Center for Biomedical Imaging, Massachusetts General Hospital, Charlestown, MA, United States

**Keywords:** central auditory system, HIV, diffusion tensor imaging, auditory working memory, graph theory

## Abstract

**Introduction:**

School-aged children experience crucial developmental changes in white matter (WM) in adolescence. The human immunodeficiency virus (HIV) affects neurodevelopment. Children living with perinatally acquired HIV (CPHIVs) demonstrate hearing and neurocognitive impairments when compared to their uninfected peers (CHUUs), but investigations into the central auditory system (CAS) WM integrity are lacking. The integration of the CAS and other brain areas is facilitated by WM fibers whose integrity may be affected in the presence of HIV, contributing to neurocognitive impairments.

**Methods:**

We used diffusion tensor imaging (DTI) tractography to map the microstructural integrity of WM between CAS regions, including the lateral lemniscus and acoustic radiation, as well as between CAS regions and non-auditory regions of 11-year-old CPHIVs. We further employed a DTI-based graph theoretical framework to investigate the nodal strength and efficiency of the CAS and other brain regions in the structural brain network of the same population. Finally, we investigated associations between WM microstructural integrity outcomes and neurocognitive outcomes related to auditory and language processing. We hypothesized that compared to the CHUU group, the CPHIV group would have lower microstructural in the CAS and related regions.

**Results:**

Our analyses showed higher mean diffusivity (MD), a marker of axonal maturation, in the lateral lemniscus and acoustic radiations, as well as WM between the CAS and non-auditory regions predominantly in frontotemporal areas. Most affected WM connections also showed higher axial and radial diffusivity (AD and RD, respectively). There were no differences in the nodal properties of the CAS regions between groups. The MD of frontotemporal and subcortical WM-connected CAS regions, including the inferior longitudinal fasciculus, inferior fronto-occipital fasciculus, and internal capsule showed negative associations with sequential processing in the CPHIV group but not in the CHUU group.

**Discussion:**

The current results point to reduced axonal maturation in WM, marked by higher MD, AD, and RD, within and from the CAS. Furthermore, alterations in WM integrity were associated with sequential processing, a neurocognitive marker of auditory working memory. Our results provide insights into the microstructural integrity of the CAS and related WM in the presence of HIV and link these alterations to auditory working memory.

## Introduction

Despite evidence of altered auditory and language processing in children perinatally infected with HIV (CPHIV), the structural integrity of white matter (WM) fibers involved in auditory neural signaling has not been investigated in this population (Chao et al., 2012; Torre et al., 2012, 2015; Rice et al., 2013; Maro et al., 2017). Throughout childhood, WM maturational changes facilitate more efficient communication between central auditory system (CAS) regions, leading to a highly organized structural network (Huang H. et al., 2015; Bourne et al., 2016). Investigating the integrity of the CAS pathway, from the cochlear nucleus (CN) and superior olivary complex (SOC) in the brainstem to the inferior colliculus (IC) in the midbrain to the medial geniculate nucleus (MGN) in the thalamus and into the primary auditory cortex (PAC) in the superior temporal lobe in children living with HIV may provide insight into observed auditory processing complications (Torre et al., 2012, 2015). Furthermore, investigating the integrity of auditory WM to non-auditory regions may lead to a greater understanding of the language-processing delays that have been reported in these populations (Rice et al., 2013).

One of the most reliable and non-invasive tools used to measure microstructural properties as markers of WM integrity is diffusion tensor imaging (DTI; Basser et al., 1994; Basser and Pierpaoli, 1996). The main DTI parameters include fractional anisotropy (FA) and axial diffusivity (AD), markers of axonal integrity, as well as mean diffusivity (MD), and radial diffusivity (RD), which reflect axonal maturation (Feldman et al., 2010). In recent years, DTI has been combined with graph theoretical frameworks to characterize tissue structural connectivity in the context of whole-brain structural networks (Fornito et al., 2016). The combination allows several network metrics to be calculated that reflect either structural segregation, as evidenced by separate clusters of highly connected nodes, or structural integration, characterized by efficient information transfer within networks.

Hearing loss in children and adolescents has been linked to lower FA in acoustic radiation, a set of WM fibers connecting the MGN to the PAC (Miao et al., 2013; Huang L. et al., 2015; Wu et al., 2016), with improved microstructural integrity, as reflected by higher FA, evident in both the PAC and acoustic radiation of children with cochlear implants (Chang et al., 2012). In addition, FA changes in the MGN, the PAC, and acoustic radiation demonstrate a strong positive correlation with language scores, suggesting a link between the microstructural changes of these regions and language development (Huang H. et al., 2015). Using a graph theoretical network, a recent study in school-aged children with auditory processing disorder reported lower betweenness centrality (how often a region is between other nodes, thus acting as a bridge or intermediary for communication between nodes in the structural network) of the inferior precentral gyrus, a region that interacts with the CAS for efficient communication (Alvand et al., 2022). Altogether, these studies show that hearing impairments not only affect CAS regions but the regions that they are anatomically linked to as well.

In CPHIVs, several imaging studies have reported alterations in brain regions related to or involved in auditory and language processing. Reduced FA and/or higher MD, in the superior/inferior longitudinal fasciculus (S/ILF), the inferior fronto-occipital fasciculus (IFOF), and the uncinate fasciculus (UF), has been reported in CPHIVs compared to age-matched controls (Li et al., 2015; Ackermann et al., 2016; Jankiewicz et al., 2017; Hoare et al., 2018; Madzime et al., 2021). Even fibers connecting subcortical auditory areas to the cortex, including the corpus callosum (CC) and the internal capsule, demonstrate microstructural and volumetric alterations in CPHIV (Ackermann et al., 2016; Randall et al., 2017; Hoare et al., 2018).

Although graph-theory-based studies in CPHIVs are limited, Li et al. (2018) reported a difference in the regional profiles of brain regions in adolescents living with HIV compared to controls. In the CPHIV group, hub regions were predominantly in sensorimotor and temporal areas, and the inferior temporal gyrus (ITG) and middle temporal gyrus (MTG) showed fewer structural connections (so-called nodal degree), pointing to organizational changes in auditory-related areas. Yadav et al. (2017) also reported changes in the regional profile of auditory-related regions including lower and higher betweenness centrality of the middle frontal regions and the fusiform, respectively. In adults living with HIV, weaker brain network structural segregation and integration have been reported (Baker et al., 2017; Bell et al., 2018). These differences were associated with immune health markers and seem to persist even in the presence of combination antiretroviral therapy (cART) (Bell et al., 2018). A recent study reported reduced structural integration (so-called nodal efficiency) of the occipital lobe and limbic cortex, as well as better structural segregation (so-called clustering coefficient) of the frontal, insula, and thalamus in adults living with HIV compared to controls (Aili et al., 2022).

We used DTI-based tractography and graph theory measures to assess WM microstructural integrity and structural organization in both the intra-auditory and inter-auditory structural networks of a cohort of 11-year-olds. We hypothesized that CPHIVs would have reduced WM integrity, marked by lower FA and higher MD, compared to children who are HIV unexposed and uninfected (CHUUs) in tracts between CAS regions, as well as tracts connecting CAS regions to the whole brain. Given previous findings of altered structural organization in auditory-related regions in CPHIV, we further hypothesized that CAS regions would have reduced strength and efficiency in CPHIV compared to CHUU in both intra- and inter-auditory networks. The children in this cohort have been followed with neuroimaging and neurocognitive testing since age 5. To investigate the potential contribution of HIV-related WM abnormalities to auditory outcomes, we examined associations with neurocognitive measures related to aspects of auditory and language abilities.

## Methods

### Study cohort

The participants were 87 children aged 11–12 years (59 CPHIVs, 28 CHUUs) who completed structural and DTI scans. The children living with HIV were recruited from the randomized Children with HIV Early Antiretroviral Therapy (CHER) trial and have been monitored since birth at the Family Center for Research with Ubuntu (FamCRU), Tygerberg Children's Hospital, in Cape Town, South Africa (Violari et al., 2008; Cotton et al., 2014). At the time of the study, all the CPHIVs were on antiretroviral therapy (ART). The uninfected children, who have also been followed longitudinally at FAMCRU, were recruited from a parallel vaccine study conducted at the same time as the CHER trial in the same community (Madhi et al., 2010). The study protocol was approved by the human research ethics committees of the Universities of Cape Town and Stellenbosch. Parents or guardians provided written informed consent, and children provided oral assent. Children were first familiarized with the scanning procedures on a mock scanner.

### T1-weighted structural and DTI acquisition

Imaging was done using a 3 Tesla Siemens Skyra Magnetic Resonance Imaging (MRI) scanner (Erlangen, Germany) at the Cape Universities Body Imaging Centre (CUBIC) of the University of Cape Town. The children underwent high-resolution (1 × 1 × 1 mm^3^) T1-weighted (T1w) structural MRI (6 min) using a multi-echo magnetization prepared rapid gradient echo sequence with repetition time (TR) = 2,530 ms, echo times (TEs) = 1.69/3.54/5.39/7.24 ms, inversion time (TI) 1,100 ms, flip angle = 7°, and FOV = 224 × 224 × 176 mm^3^, and two whole-brain DTI acquisitions with the phase encoding direction inverted to correct for echo planar imaging (EPI)–related distortions at air–tissue interfaces (7 min each). Acquisition parameters for diffusion were TR/TE 11,100/92 ms, 84 slices, 2 × 2 × 2 mm^3^, Field of View (FOV) = 244 × 244 × 168 mm^3^, 30 non-collinear diffusion directions, b = 1,000 s/mm^2^, and 5 non-diffusion-weighted (b0) acquisitions.

### Image preprocessing

The process described here was done for each subject. Both T1w structural and DTI data were converted from DICOMs to NIFtI readable format. Subsequently, we created a T2-weighted (T2w) anatomical image imitation of the T1w structural image. For this step, we used the *fat_proc_imit2w_from_t1w* function from the Analysis of Functional Neuroimages (AFNI) software package (Cox, 1996). The T2w volume would serve as the structural reference for DTI processing. DTI data were processed using the Tolerably Obsessive Registration and Tensor Optimization Indolent Software Ensemble (TORTOISE) version 3.2 (Pierpaoli et al., 2009). We began by visually inspecting the DTI data (in both anterior–posterior, AP, and posterior–anterior, PA, directions) and manually removing all distorted volumes or volumes with drop-out slices. Subjects with < 15 diffusion-weighted (DW) volumes and 1 b0 volume remaining were excluded from further analyses. With the T2w volume as the reference, motion, eddy, and EPI distortions were corrected using TORTOISE's *DIFFPREP* and *DRBUDDI* functions. Subsequently, we used the *fat_proc_dwi_to_dt* function to estimate diffusion tensors and DTI parameters (FA, MD, etc.).

### Gray matter regions of interest

In this study, we were interested in examining WM integrity in pathways within and to the auditory network. To define intra-auditory network pathways, we used four bilateral manually traced gray matter (GM) regions that form part of the CAS as seeds. These included a region at the junction between the brainstem pons and the medulla that consists of the CN/SOC complex, the IC, the MGN, and the PAC. We used AFNI's *3dNwarpApply* (AFNI, 2014) to co-register the DTI and structural T1w data and map regions of interest (ROIs) to DTI space.

**Figure 1:**
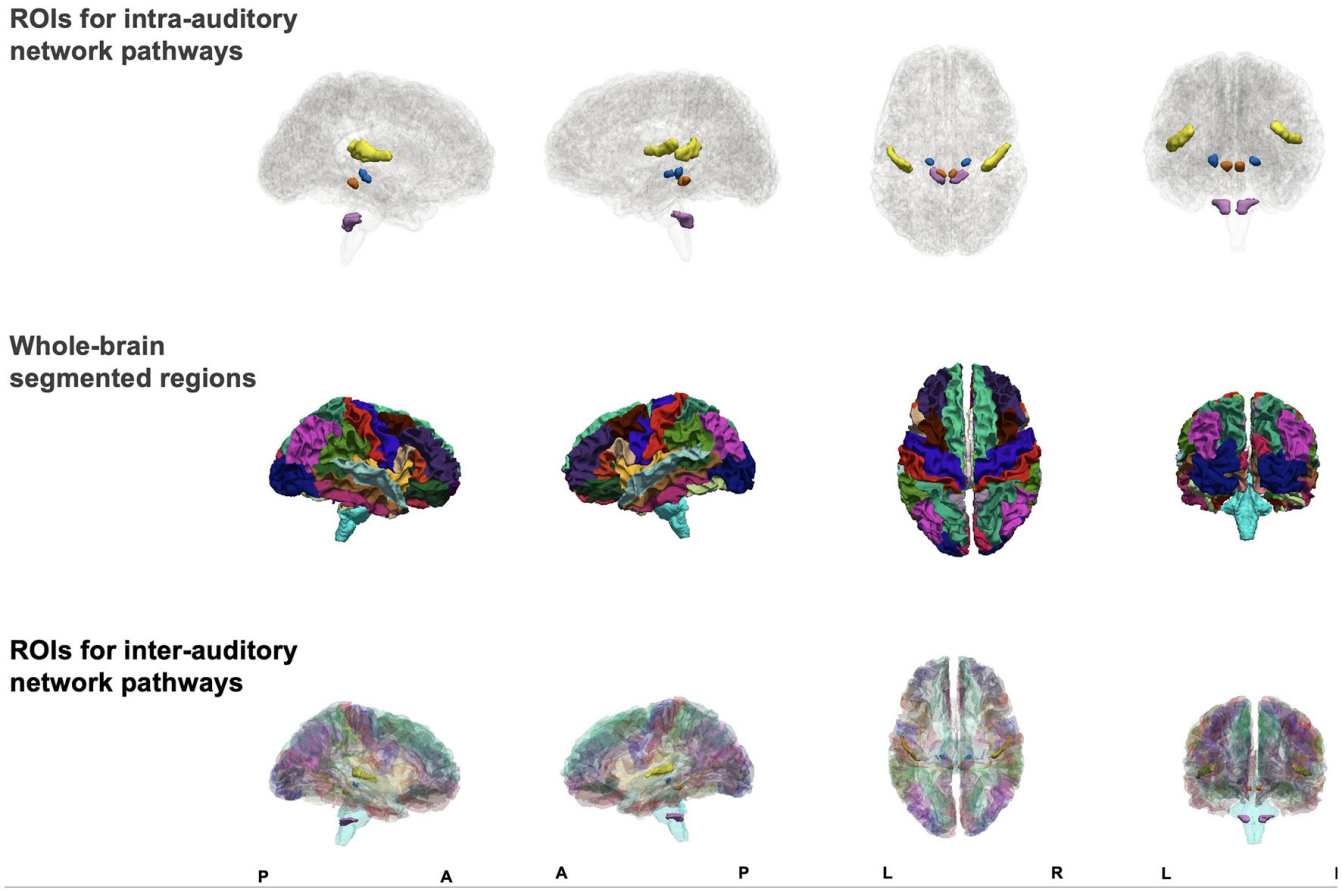
Figure showing sagittal, axial, and coronal views of manually traced regions of interest (ROIs) for intra-auditory pathway analysis **(top row)**, automatically segmented whole-brain ROIs **(middle row)**, and combined ROIs **(bottom row)** to form one atlas for inter-auditory network pathway analysis.

The inter-network pathways were obtained using a set of automatically segmented ROIs of the whole brain merged with the manually traced auditory pathway ROIs. We used Connectome Mapper (CMP) 3 (v3.0.0-RC4) to automatically segment the brain and produce multi-resolution (*N* = 5) morphological subject atlases (Daducci et al., 2012; Tourbier et al., 2022). In the current study, we used the lowest scale, comprising 126 subcortical and cortical segmentations. A list of all ROIs in the atlas and their abbreviations are given in Appendix A. We combined the automatically segmented ROIs with the manually traced auditory ROIs using *3dcalc* (Figure 1). Where the manually traced ROIs overlapped with the automatically segmented ROIs, we excluded the entire automatically segmented ROI and maintained the manually traced ROI. In this way, a whole-brain atlas was created for each subject (Figure 1).

### DTI-based tractography

Prior to performing tractography, all ROIs in our whole brain set were inflated by 1 mm using 3dROIMaker to ensure they would extend into WM. Because our initial tractography using the manually traced seeds did not produce key auditory tracts, we further inflated the manually segmented auditory seeds in 1-mm increments until key tracts were generated. Key auditory tracts were successfully reconstructed after inflation by 4 mm.

Tractography was performed using the Functional and Tractographic Connectivity Analysis Toolbox (FATCAT, version 1.1; Taylor and Saad, 2013) in AFNI. The *3dTrackID* function was used to perform full probabilistic tractography (Taylor et al., 2015). The algorithm options included FA ≥ 0.2, angle ≤ 50°, length ≥ 10 mm, number of starting seeds per voxel per Monte Carlo iteration ≥ 5, number of Monte Carlo iterations = 1,000. The *3dTrackID* outputs are *N* × *N* adjacency matrices for each DTI measure (FA, MD, AD, RD) of the means and the standard deviations of the WM pathways connecting pairs of ROIs; *N* is the number of ROIs. Other outputs include the fractional number of tracks/streamlines (fNT), defined as the ratio of the number of streamlines in a WM bundle to the total number of streamlines in the whole brain, and the physical volume (PV) of a tracked WM bundle in mm^3^.

### Structural network definition

**Figure 2:**
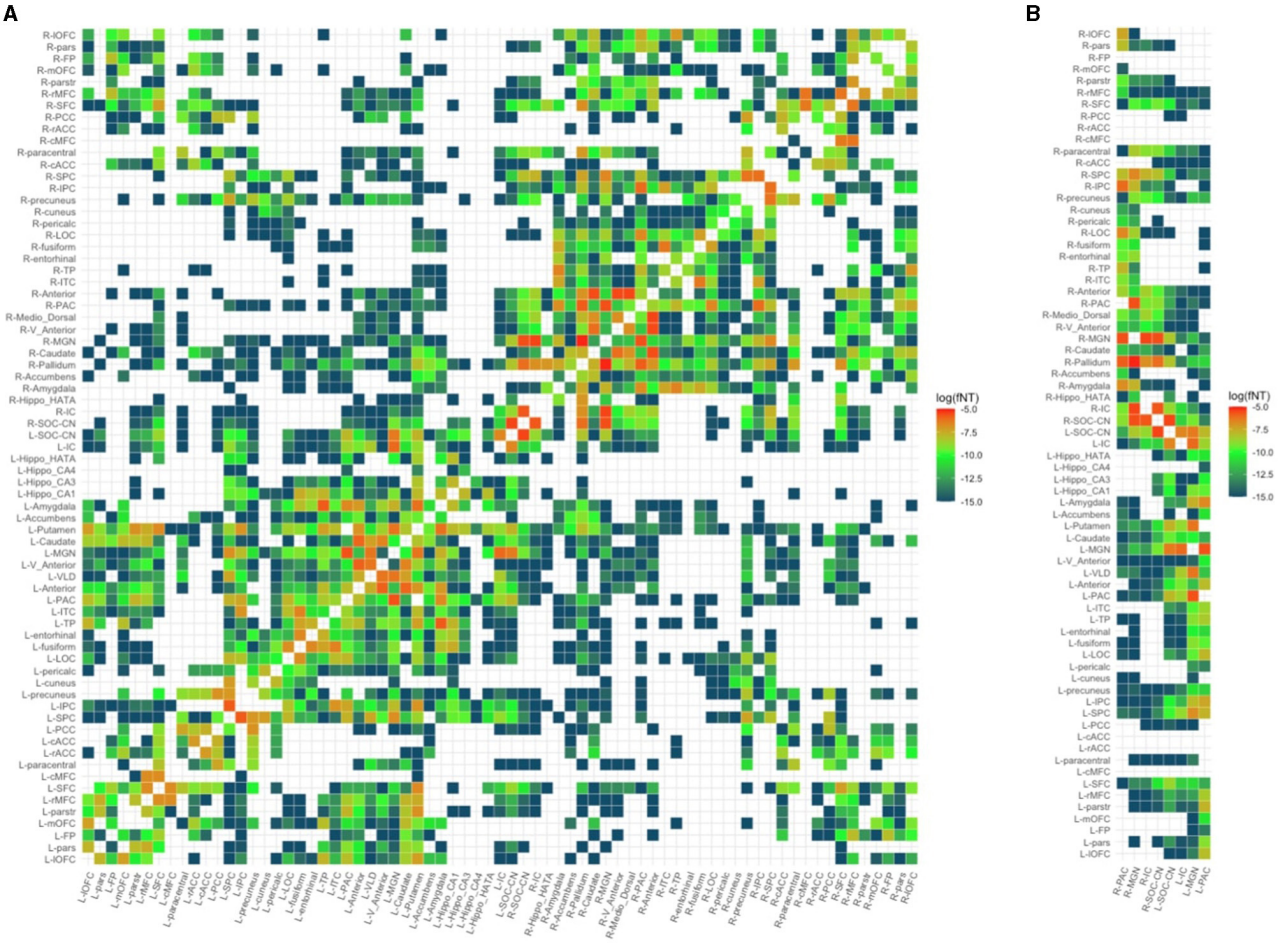
Structural network of a random control subject. **(A)** Full 71 × 71 structural connectivity matrix. **(B)** Reduced matrix showing only columns with auditory regions of interest. PAC, primary auditory cortex; MGN, medial geniculate nucleus; IC, inferior colliculus; SOC/CN, superior olivary complex/cochlear nucleus; L/R_parstr, parstriangularis; L/R_pars, parsorbitalis; L/R_parso, parsopercularis; L/R_rMFC, rostral middle-frontal cortex; L/R_SFC, superior frontal cortex; L/R_cMFC, caudal middle-frontal cortex; L/R_precentral, precentral gyrus; L/R_paracentral, paracentral gyrus; L/R_rACC, rostral anterior cingulate cortex; L/R_cACC, caudal anterior cingulate cortex; L/R_PCC, posterior cingulate cortex; L/R_Isth, isthmus; L/R_postcentral, postcentral gyrus; L/R_SPC, superior parietal cortex; L/R_IPC, inferior parietal cortex; L/R_pericalc, pericalcarine; L/R_LOC, lateral occipital cortex; L/R_lingual, lingual gyrus; L/R_bankssts, banks of superior temporal sulcus; L/R_Anterior, anterior thalamic nucleus; L/R_Medio_Dorsal, medio-dorsal thalamic nucleus; L/R_VLD, ventral latero-dorsal thalamic nucleus; L/R_CLLPM_Pulvinar, central latero-lateral posterior medial pulvinar; L/R_V_Anterior, ventral anterior thalamic nucleus; L/R_Hippo, hippocampus; L/R_Hippo_Pres, hippocampus presubiculum; L/R_Hippo_CA1,3,4, hippocampus cornu ammonis 1,3,4; L/R_Hippo_Mol_layer_HP, hippocampus molecular layer; L/R_Hippo_Tail, hippocampus tail; L/R, left/right.

3dTrackID was used to extract a fNT matrix for every subject. The nodes of these matrices include all the ROIs for which WM connections to other ROIs exist, and the matrix elements are the fNT values for each pair-wise connection. To maintain the same number of ROIs for all subjects in the sample, we identified ROIs that were common to the fNT matrices of all subjects and created individual subject connectivity matrices based on this reduced set of ROIs. Because the number of common ROIs was 71, each subject's structural network was defined by a 71 × 71 (Figure 2) connectivity matrix. The fNT matrices were not thresholded as fNT represents the probability that a WM connection exists between a pair of nodes. Furthermore, thresholding structural network matrices has been reported to have no statistical effect in the estimation of network measures (Colon-Perez et al., 2016; Civier et al., 2019).

### Statistical analyses and visualization

After defining WM connectivity matrices for each subject, spurious WM connections were identified. Spurious WM connections were defined as connections not present in at least 95% of the children. For the remaining connections, FA, AD, RD, MD, fNT, and PV were extracted.

Graph nodal measures were computed for each subject's structural network using the iGraph (v 1.3.1) and brainGraph (v 3.0) packages in R (v 4.1.3) and based on fNT values. Nodal parameters computed included degree, a measure of how many connections a node has; strength, a measure of the density of connections to the node; transitivity, a measure of densely connected groups of nodes; nodal efficiency, a measure of the efficiency of communication between a node and all the other nodes in the network; and local efficiency, a measure of the efficiency of the graph formed in the absence of the node as an indication of the node's level of fault tolerance (Fornito et al., 2016).

Then, for each DTI parameter and nodal measure identified, outliers by connection or node, respectively, were identified. Outliers were defined as values more than 1.5 times the interquartile range (IQR) above or below the upper and lower quartiles, respectively. We subsequently ran linear models for the set of connections and nodes to compare groups, CPHIV vs. CHUU. If including the outliers in the group comparison resulted in a different result than when not included, we excluded the outliers from the analysis. In addition, although both males and females experience significant WM maturation during adolescence, sex differences in trajectory rates have been reported (Simmonds et al., 2014; Gur and Gur, 2016). Sex was therefore included as a confounding variable in all models. Intracranial volume (ICV) was included to control for head size differences, and we examined handedness as a potential confounder. We corrected for multiple comparisons using the false discovery rate (FDR) method. We chose an FDR *q*-value of 0.05 as indicating significance.

The iGraph package in R was used to visualize our results. Additionally, we used Freesurfer (v7.2) to visualize the implicated WM connections. As far as possible we attempted to identify the implicated WM connections in the context of known and established WM connections. To do this, we employed the Johns Hopkins University International Consortium for Brain Mapping (JHU ICBM) tracts (https://neurovault.org/images/1400/) and labels (https://neurovault.org/images/1401/) as WM atlases. After identifying the WM connections that showed significant differences between groups, we used *3dTrackID* to generate masks of the probabilistic representation of each affected WM connection in one random control subject. We then registered the WM atlases to the subject space and identified the possible WM tract by finding the largest overlap between the identified WM connection and the WM atlas.

#### Associations between measures of structural network integrity and cognitive outcomes

We refer to the DTI parameters (FA or MD, AD and RD) and graph measures (degree, strength, transitivity, and nodal and local efficiency) as structural integrity outcomes in this section. We examined the association between structural integrity outcomes and a subset of neurocognitive measures directly and indirectly related to auditory and language function. Tests included the Kaufman Assessment Battery for Children–Second Edition (KABC-II; Kaufman and Kaufman, 2014) in the current cohort and at the same age. Although this battery was developed and standardized in the United States, there is evidence of its utility cross-culturally (Van Wyhe et al., 2017). The KABC neurocognitive measures included in our analyses were Number Recall (pure auditory); Word Order (visual and short-term memory); Learning, Atlantis, Atlantis Delayed, Rebus, Rebus Delayed, and Delayed Recall (long-term storage and retrieval); Story completion (visuospatial); Sequential Processing (auditory working memory); Mental Processing (global cognitive score); and Non-Verbal Index. Additionally, we included a test of verbal or phonematic fluency (Strauss et al., 2010) called Animal Naming, in which children must generate words within a given category.

To investigate the associations, we ran a Pearson correlation between structural integrity outcomes and neurocognitive scores. Where a variable was not normally distributed (Shapiro–Wilk p < 0.05), we investigated the relationship via a Spearman correlation statistical test as it is resistant to outliers. In addition, we investigated the interaction between groups and neurocognitive scores with microstructural integrity. We corrected for multiple comparisons using the FDR method and considered FDR q < 0.05 as our significance level.

#### Associations between structural integrity outcomes and immune health markers

Within the CPHIV group, we examined associations of structural integrity outcomes with immune health markers. Correlation tests were either Pearson or Spearman depending on the normality of the immune health markers. We investigated early immune health (CD4% at study enrollment, age 6–8 weeks), immune health of participants at the time of the 11-year scan, and treatment-related health markers [age at cART initiation and first viral load (VL) suppression]. We corrected for multiple comparisons using the FDR method and considered FDR *q* < 0.05 as our significance level.

### Investigating the robustness of results

Due to the imbalance in the group sample sizes (59 CPHIVs vs. 22 CHUUs), we investigated the robustness of the results by running the same-group comparisons on 10 random CPHIV samples equivalent to the final CHUU sample. We ran the comparisons between FA, MD, AD, and RD.

## Results

We excluded 6 (3 CPHIVs and 3 CHUUs) subjects due to motion artifacts present in more than half of the DW volumes. On average each participant had 28 ± 3 DW volumes and 3 ± 1 b0s and there were no significant group differences. Further, we excluded data from 10 (7 CPHIVs and 3 CHUUs) children who had less than half of the average number of connections in the sample. We therefore present results for 22 CHUUs and 49 CPHIVs. Sample demographics are given in Table 1. Notably, groups did not differ in age. Among CPHIVs, roughly two-thirds had initiated ART before age 12 weeks and 96% were virally suppressed at the time of the scan.

**Table 1:** Sample characteristics of CPHIVs and CHUUs (N = 71).

	**CHUUs**	**CPHIVs**	***p*-value**	**FDR *q*-value**
**Demographics**				
*N*	22	49		
Sex: Female *N* (%)	8 (36%)	28 (57%)	0.27	
Age at scan: Mean + *SD* (yr)	11.65 ± 0.25	11.62 ± 0.28	0.58	
**Clinical measures at baseline (age 6–8 weeks)**				
CD4 count (cells/mm^3^) (Mean ±*SD*)		1842.76 ± 861.40		
CD4% (Mean ±*SD*)		34.65 ± 9.61		
High VL (>750,000) *N* (%)		28 (57)		
Low VL (400–750,000) *N* (%)		21 (43)		
Suppressed VL (< 400) *N* (%)		0 (0)		
**Clinical measures at scan**				
CD4 count (cells/mm^3^) (Mean ±*SD*)		913.63 ± 403.74		
CD4% (Mean ±*SD*)		38.64 ± 7.32		
High VL (>750,000) *N* (%)		0 (0)		
Low VL (400–750,000) *N* (%)		2 (4)		
Suppressed VL (< 400) *N* (%)		47 (96)		
**Treatment-related measures**				
Age at ART initiation (weeks; mean ±*SD*)		16.03 ± 14.25		
ART initiation before age 12 weeks, *N* (%)		34 (69)		
Age at ART interruption (weeks; *N* = 26^†^; mean ±*SD*)		77.82 ± 28.38		
Duration of ART interruption (weeks; *N* = 26^†^; mean ±*SD*)		82.48 ± 106.33		
**Language-related measures**				
Sequential processing (Median ± IQR)	82.13 ± 14.63	76.17 ± 9.21	0.67	0.73
Number recall (Mean ±*SD*)	7.41 ± 2.54	6.82 ± 2.13	0.22	0.46
Word order (Mean ±*SD*)	6.42 ± 2.42	5.29 ± 1.84	0.53	0.63
Learning (Mean ±*SD*)	91.91 ± 13.24	88.20 ± 13.83	**0.03**	0.35
Atlantis (Median ± IQR)	10.2 ± 2.11	9.20 ± 2.34	0.29	0.47
Atlantis delayed (Median ± IQR)	9.20 ± 1.68	9.18 ± 2.26	0.25	0.46
Rebus (Mean ±*SD*)	6.5 ± 3.22	6.16 ± 2.66	0.08	0.35
Rebus delayed (Mean ±*SD*)	6.86 ± 2.82	6.02 ± 2.61	0.81	0.81
Story completion (Mean ±*SD*)	7.45 ± 3.25	7.27 ± 2.45	0.24	0.46
Delayed recall (Median ± IQR)	88.55 ± 10.73	85.17 ± 12.31	0.41	0.53
Animal naming (Mean ±*SD*)	11.37 ± 4.19	10.31 ± 3.20	0.20	0.46
Non-verbal index (Median ± IQR)	80.11 ± 15.21	85.13 ± 10.20	0.35	0.51
Mental processing (Mean ±*SD*)	82 ± 13.77	79.94 ± 9.44	0.06	0.35

Where a variable was not normally distributed (Shapiro–Wilk p < 0.05), we summarize with the median and the IQR.

CPHIVs, children perinatally infected and living with HIV; CHUUs, children who are HIV unexposed and uninfected; FDR, false discovery rate; VL, viral load; ART, antiretroviral therapy; IQR, interquartile range. ^†^23 subjects were not interrupted and received ART continuously. *p*-values less than 0.05 are bold.

### DTI-based tractography results: WM microstructural integrity

We found a total of 498 WM connections present in at least 95% of the children. Of these WM connections, 18 were within the auditory network (intra-auditory pathways), 109 were from/to the auditory network (inter-auditory pathways), and the rest were between non-auditory regions (other intra- and inter-network pathways). Because the focus of this study is auditory processing, we only examined structural integrity in intra- and inter-auditory pathways. In the intra-auditory pathway, these connections were between 4 bilateral ROIs, and in the inter-auditory pathway between these same 4 bilateral ROIs and 45 whole brain ROIs.

**Figure 3:**
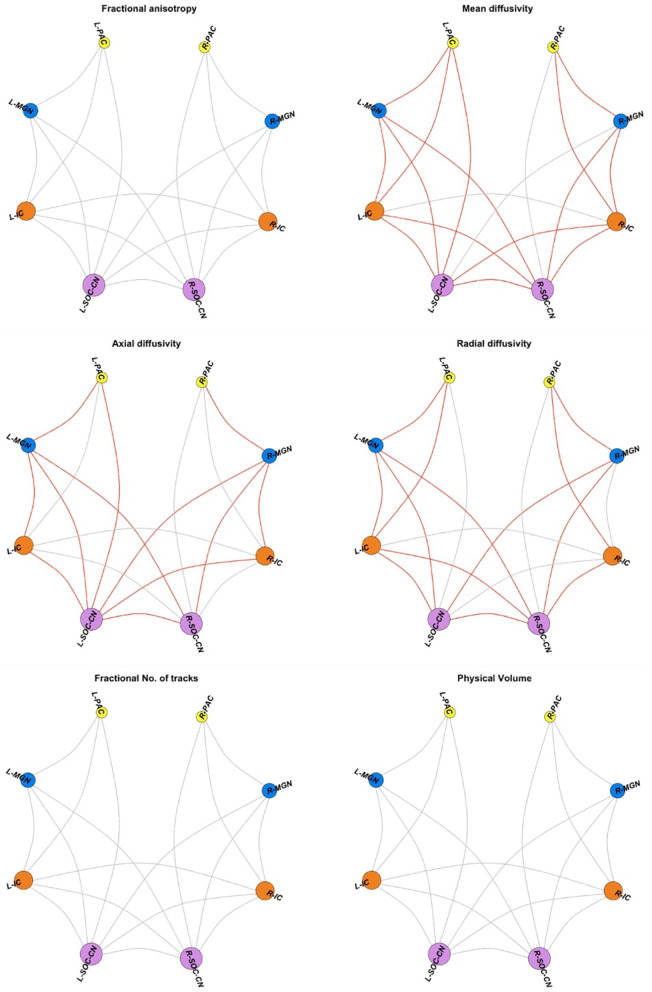
Intra-auditory pathway. Spheres are the four bilateral auditory regions of interest (ROIs); gray links represent diffusion tensor image (DTI) measures for white matter (WM) connections between pairs of ROIs. DTI measures include fractional anisotropy (FA), mean diffusivity (MD), axial diffusivity (AD), radial diffusivity (RD), the fractional number of tracks/streamlines (fNT), and physical volume (PV). Red links indicate WM connections showing higher values for the relevant DTI measure in CPHIVs compared to CHUUs. ROIs are sized according to the number of connections to the region. PAC, primary auditory cortex; MGN, medial geniculate nucleus; IC, inferior colliculus; SOC/CN, superior olivary complex/cochlear nucleus; R, right; L, left.

We found no significant group differences in FA in any WM connections investigated. Among the 18 intra-auditory WM connections, 15 connections demonstrated higher MD in CPHIVs compared to CHUUs (Figure 3). Of these, 8 connections had higher AD and RD, 3 had higher AD only, and 4 had higher RD only. One WM connection (L SOC/CN to R MGN) demonstrated higher AD and RD but no MD increase. None of the intra-auditory connections demonstrated group differences in fNT or PV.

**Figure 4:**
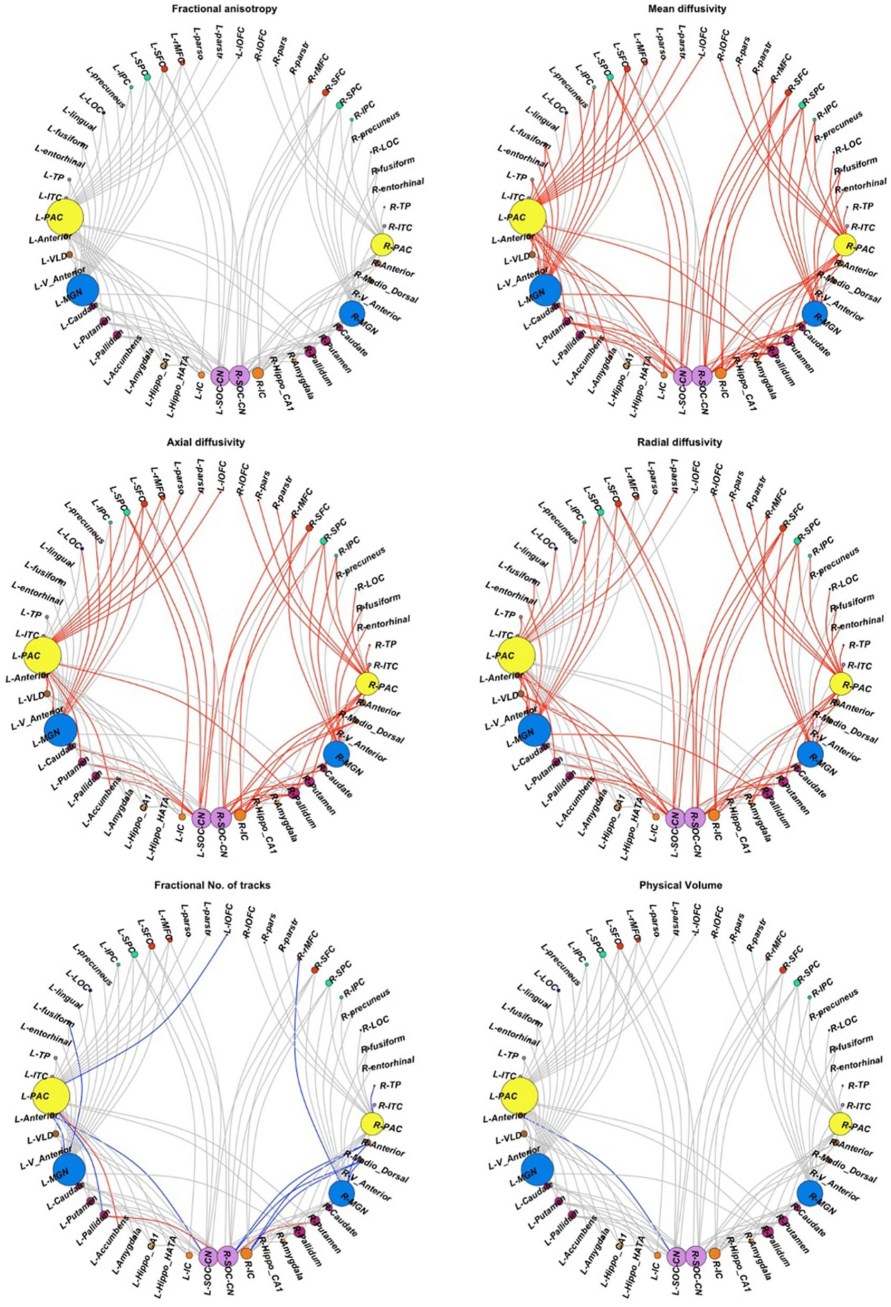
Inter-auditory pathway. Forty-five non-auditory regions of interest (ROIs) exhibited white matter (WM) connections (indicated by gray links) to the four bilateral auditory ROIs. Red links indicate WM connections where CPHIVs have higher values on the measure shown than CHUUs, while blue links indicate WM connections where CPHIVs have lower values than CHUUs. ROIs are sized according to the number of links to the ROI. The ROIs are organized relatively uniformly from the left and right starting at the CN/SOC (cochlear nucleus/superior olivary complex) in the middle bottom of the sphere (purple) and moving on either side to the IC (inferior colliculus, orange), other midbrain ROIs (orange), basal ganglia ROIs (purple), the MGN (medial geniculate nucleus, blue), thalamic nuclei (brown), the PAC (primary auditory cortex, yellow), temporal lobe ROIs (gray), occipital lobe ROIs (royal blue), parietal lobe ROIs (pastel green), and frontal lobe ROIs (red). L/R_parstr, parstriangularis; L/R_ pars, parsorbitalis; L/R_parso, parsopercularis; L/R_rMFC, rostral middle-frontal cortex; L/R_SFC, superior frontal cortex; L/R_cMFC, caudal middle-frontal cortex; L/R_precentral, precentral gyrus, L/R_paracentral, paracentral gyrus; L/R_rACC, rostral anterior cingulate cortex; L/R_cACC, caudal anterior cingulate cortex; L/R_PCC, posterior cingulate cortex; L/R_Isth, isthmus; L/R_postcentral, postcentral gyrus; L/R_SPC, superior parietal cortex; L/R_IPC, inferior parietal cortex; L/R_pericalc, pericalcarine; L/R_LOC, lateral occipital cortex; L/R_lingual, lingual gyrus; L/R_bankssts, banks of superior temporal sulcus; L/R_PAC, primary auditory cortex; L/R_Anterior, anterior thalamic nucleus; L/R_Medio_Dorsal, medio-dorsal thalamic nucleus; L/R_MGN, medial geniculate nucleus; L/R_VLD, ventral latero-dorsal thalamic nucleus; L/R_CLLPM_Pulvinar, central latero-lateral posterior medial pulvinar; L/R_V_Anterior, ventral anterior thalamic nucleus; L/R_Hippo, hippocampus; L/R_Hippo_Pres, hippocampus presubiculum; L/R_Hippo_CA1,3,4, hippocampus cornu ammonis 1,3,4; L/R_Hippo_Mol_layer_HP, hippocampus molecular layer; L/R_Hippo_Tail, hippocampus tail; L/R_IC, inferior colliculus; L/R_SOC_CN, cochlear nucleus/superior olivary complex; L/R, left/right.

Among the inter-auditory WM connections, we found 84 connections with higher MD in CPHIVs than CHUUs, 56 connections with higher AD, 64 with higher RD, 4 with higher fNT, 13 with lower fNT, and 1 connection with lower PV (Figure 4). Summary statistics for all 127 auditory WM connections across DTI parameters can be found in Appendix B.

### Graph theory results: structural network organization

None of the auditory ROIs demonstrated differences in nodal measures between the CHUU and CPHIV groups. However, the CPHIV group showed lower strength in non-auditory ROIs, including the right anterior thalamic nucleus, the right mediodorsal thalamic nucleus, the right ventral anterior thalamic nucleus, and the left lateral orbitofrontal cortex (lOFC). The CPHIV group also showed higher transitivity in the left accumbens compared to the CHUU group (Table 2). Summary statistics for all nodes between groups can be found in Appendix C.

**Table 2:** ROIs showing differences in nodal measures between CHUUs and CPHIVs.

**Node**	**Degree**	**Strength**	**Transitivity**	**Nodal efficiency**	**Local efficiency**
R anterior thalamic nucleus	–	**↓**	–	–	–
R mediodorsal thalamic nucleus	–	**↓**	–	–	–
R ventral anterior thalamic nucleus	–	**↓**	–	–	–
L accumbens	–	–	↑	–	–
L lOFC	–	**↓**	–	–	–

ROI, region of interest; CHUUs, children who are HIV unexposed and uninfected; CPHIVs, children perinatally infected and living with HIV; R, right; L, left; lOFC, lateral orbitofrontal cortex.

### Associations between structural integrity outcomes and cognitive measures

There were no significant HIV differences in auditory and language-related outcomes. Unadjusted results find CPHIVs with lower mean scores in the Learning domain compared to CHUUs (*p* = 0.03; Table 1).

**Figure 5:**
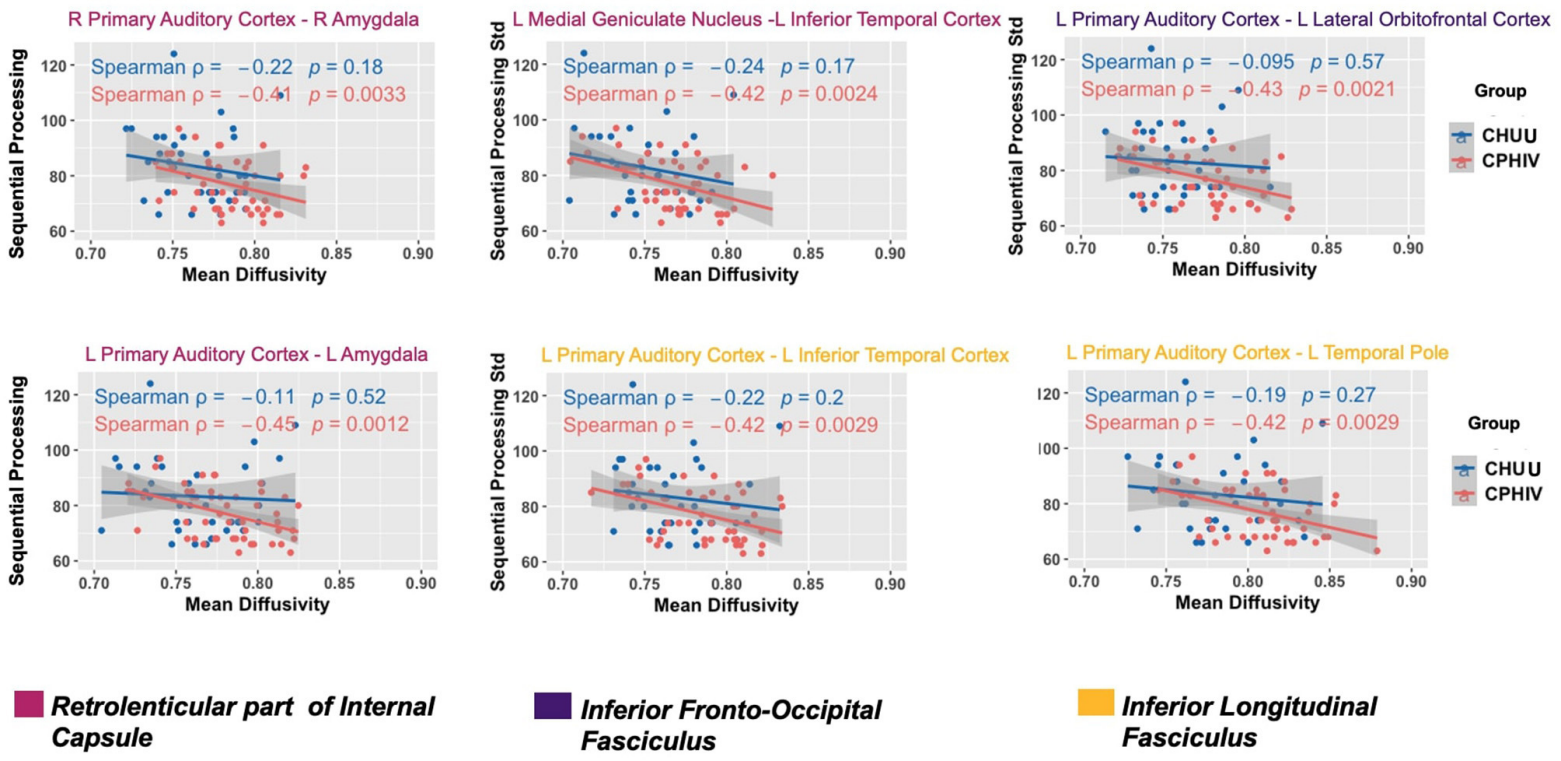
White matter (WM) connections where increasing mean diffusivity (MD) was associated with poorer sequential processing in CPHIVs but not in CHUUs. Two WM connections belonged to fibers of the left retrolenticular part of the internal capsule (RPIC; magenta): (L) Primary auditory cortex to (L) amygdala, (L) medial geniculate nucleus to (L) inferior temporal cortex and one connection belonged to the right RPIC (magenta): (R) primary auditory cortex to (R). One connection belonged to fibers of the (L) inferior fronto-occipital fasciculus (purple): (L) primary auditory cortex to (L) lateral orbitofrontal cortex; two WM connections belonged to fibers of the (L) inferior longitudinal fasciculus (yellow): (L) primary auditory cortex to (L) inferior temporal cortex, (L) primary auditory cortex to (L) temporal pole. To identify the WM fibers that each connection likely belongs to, we identified the areas of the WM atlases, JHU ICBM tracts (https://neurovault.org/images/1400/) and labels (https://neurovault.org/images/1401/), where there was most overlap with the probabilistic representation of each WM connection. The second and third rows are R/L, or right/left.

**Figure 6:**
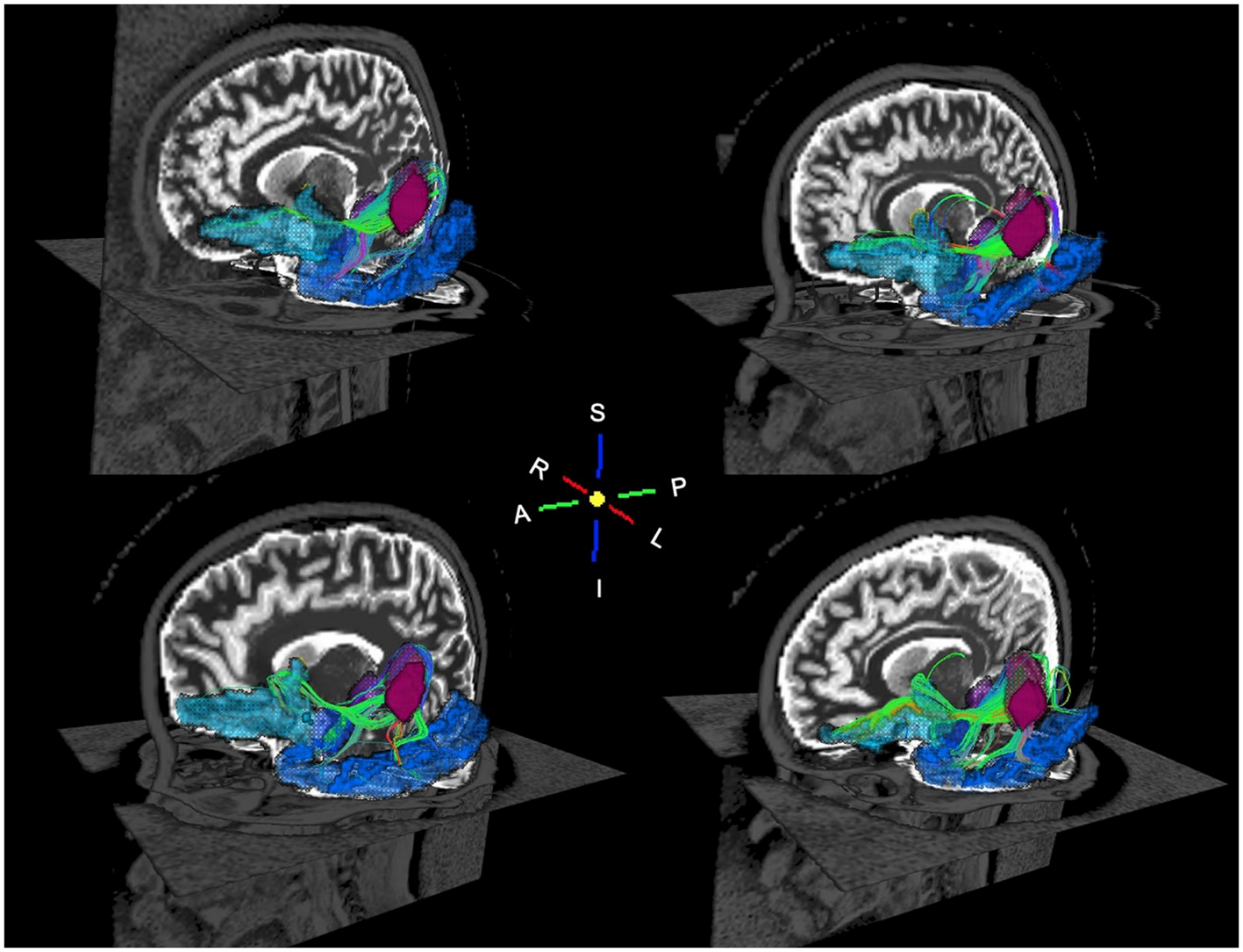
Oblique views of probabilistic tractography white matter streamlines for four random subjects. The streamlines connect the left primary auditory cortex (magenta) to the left amygdala (purple), left lateral orbitofrontal cortex (cyan), and left inferior temporal cortex (blue). The WM streamlines are colored according to the spatial orientation of the WM fibers: red is left to right, green is anterior to posterior, and blue is inferior to superior.

Increasing MD was associated (FDR *q* < 0.05) with poorer sequential processing in six WM connections in CPHIVs but not CHUUs (Figure 5). Notably, all six of these WM connections were among those showing higher MD in CPHIVs than CHUUs in our inter-auditory tractography analysis and were identified as belonging to the right and left retrolenticular part of the internal capsule, the left IFOF, and the left ILF. There were no significant group interactions in all tracts except the connection between the left MGN and left ITC (internal capsule; Figure 5). All six WM connections showed a negative association between MD and sequential processing in the whole sample (Spearman rhos between −0.32 and −0.37, all *p*s < 0.007). The probabilistic representations of the connections that were to the left PAC are shown in Figure 6 for four random subjects. We found no significant associations between other structural integrity outcomes and neurocognitive scores.

#### Associations between structural integrity outcomes and immune health markers

We found no significant associations between structural integrity outcomes and immune health makers after correcting for multiple comparisons.

### Investigating the robustness of results

**Figure 7:**
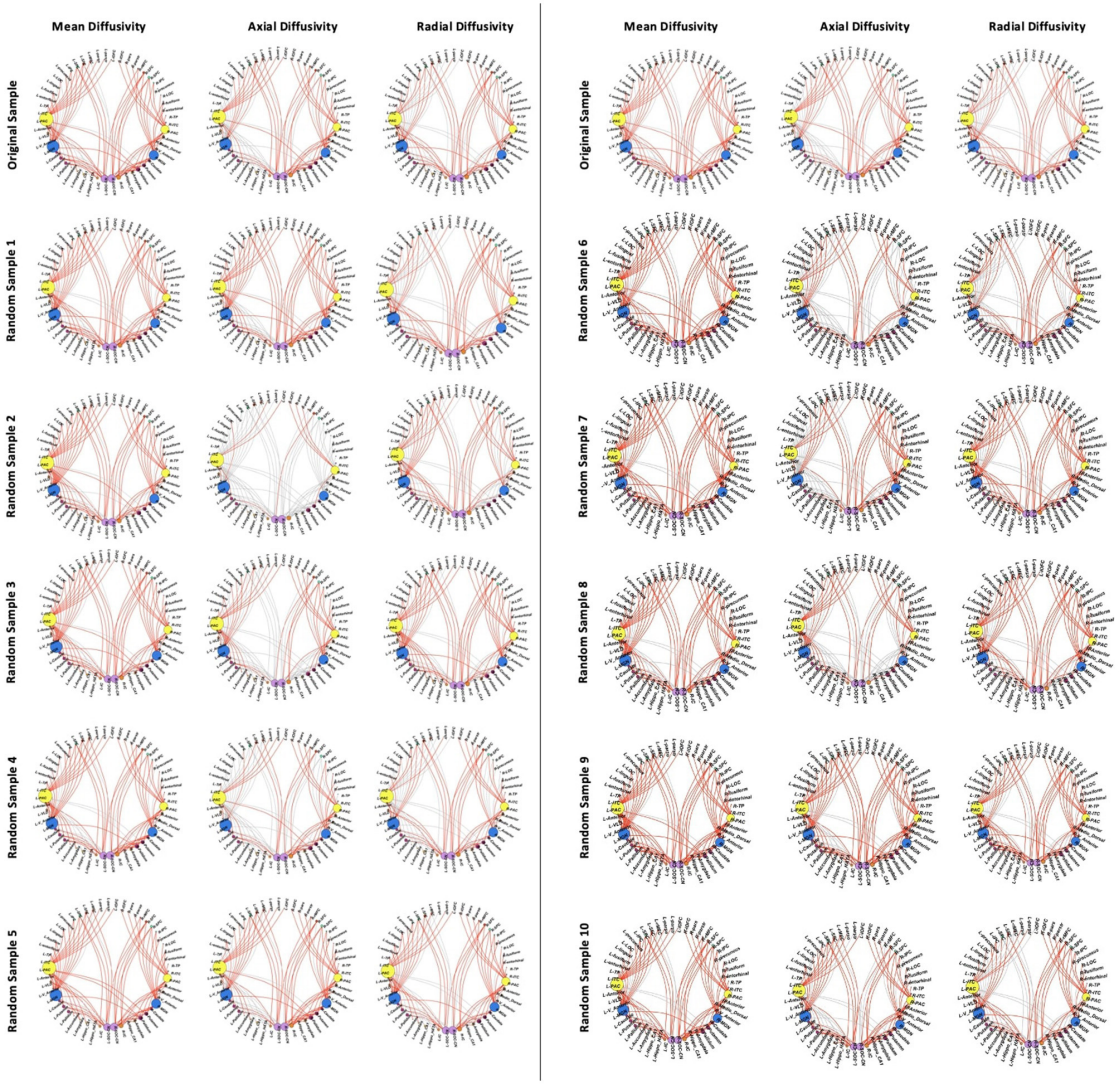
White matter connections showing group differences in the reported sample **(top row)** vs. connections showing differences in 10 samples.

With the final CHUU sample being *N* = 22, we ran 10 comparisons per DTI parameter, using 10 random samples. There were no differences in FA for all 10 group comparisons. The analysis found that the majority of connections with HIV-related differences in MD, AD, and RD persisted. Table 3 summarizes the comparison between each sampling and the main analysis between 49 CPHIVs and 22 CHUUs. We report that the same HIV group differences showed up as significant 89%, 73%, and 82% times, respectively, in the random samples. Figure 7 shows the connections that came up as significant in the main analysis as well as the results from the comparisons between the CHUU group and 10 random samples. Despite variability between each comparison, connections along the auditory pathway were significantly different across all comparisons and parameters.

**Table 3:** Total connections reported as significant in each modality vs. the total number of connections reported as significant in 10 random samples of 22 CPHIVs.

**Sample**	**MD**		**AD**		**RD**	
**Main analysis**	**99**	**%**	**68**	**%**	**77**	**%**
1	91	92	63	93	72	94
2	82	83	19	28	50	65
3	84	85	41	60	60	78
4	86	87	52	76	56	73
5	91	92	65	96	70	91
6	85	86	37	71	52	68
7	92	93	49	72	70	91
8	86	87	43	63	62	81
9	93	94	62	91	69	90
10	92	93	62	91	68	88

The % columns show the percentage of tracts in the random sample results that are the same as the ones found in the main analysis. CPHIVs, children prenatally infected and living with HIV; MD, mean diffusivity; AD, axial diffusivity; RD, radial diffusivity.

Additionally, connections to non-auditory regions, particularly to frontotemporal regions, basal ganglia, and thalamic regions were significantly different across all comparisons (Figure 7). The comparison with the least number of significant connections across parameters (Sample 2 in Table 3) demonstrated higher MD, RD, and AD between auditory regions as well as between the same non-auditory regions. The means and standard deviations of connections across all 10 CPHIV samples are similar to the main analysis (Appendix D).

## Discussion

The current study investigated the structural integrity of the CAS in relation to HIV infection in preadolescence. Our main interest was studying WM within the CAS, and our analysis identified reduced axonal maturation axonal maturation within the auditory pathway. In addition, we identified many connections between auditory and non-auditory regions also with HIV-related reduced WM maturation. We further identified altered nodal profiles in the thalamus, which plays an important role in linking the CAS with other brain functions. Taken together, these results suggest children living with HIV who start treatment early demonstrate delayed WM maturation within the central auditory system, as well as between the auditory system and the rest of the brain.

In CPHIVs, we found 6 inter-auditory WM connections in which MD increases were related to poorer auditory working memory ability. However, only one connection demonstrated a significant HIV group interaction. As a result, we are not able to confidently link these to HIV-related changes in WM at age 11 years in this cohort. Future longitudinal work is needed to understand the functional consequences of the reported alterations in WM in the CAS.

### HIV-related WM disruptions within auditory tracts

Higher MD throughout the auditory pathway suggests a risk of disrupted auditory signaling from the level of the brainstem to the cortex in the presence of HIV. The auditory pathway, including the lateral lemniscus fibers located between the CN/SOC and the IC, as well as the acoustic radiations located between the MGN and PAC, also demonstrate altered structural integrity in children with hearing impairments (Wu et al., 2009; Huang H. et al., 2015), and therefore, our results could be related to auditory function. Even though the current cohort did not have differences in hearing sensitivity tests performed on brainstem response, future work is needed to ascertain whether differences in the CAS relate to other peripheral auditory functions. In addition, longitudinal analyses would provide a better understanding of the nature of the disruptions, if they represent delayed maturation or HIV-related damage.

### HIV-related WM disruptions between auditory and non-auditory areas

Higher MD in CPHIVs compared to CHUUs was persistent in tracts between auditory and non-auditory seeds, which included predominantly temporal and frontal lobe regions. The majority of affected WM demonstrated higher AD and RD in the CPHIV group, with almost no differences in fNT or PV, the putative markers of the density of axons in probabilistic WM connections. These accompanying parameters may provide insight about the underlying neurobiology. Simultaneous increases in MD, AD, and RD, such as is seen here in CPHIVs, have been linked to chronic diseases associated with intensive axonal loss (Lin et al., 2016; Winklewski et al., 2018). Axonal loss is characterized by increased spaces within and between axons, resulting in increased isotropic diffusion that enhances both AD and RD. WM tracts with increased AD and MD may be related to decreased intra-axonal water diffusivity. AD and MD are typically inversely related in healthy tissue (Altaye et al., 2013; Simmonds et al., 2014; Uda et al., 2015) making the underlying neurobiological architecture unclear. A similar pattern has been seen in neurodegenerative diseases that cause a combination of inflammation, neuronal atrophy, and changes in tissue WM microstructure (Sbardella et al., 2013). By comparison, tracts demonstrating increased RD and MD, without changes, in AD may be due to increased restriction of diffusion arising from the axonal loss possibly caused by inflammation (Kim et al., 2007; Xie et al., 2010). Axonal damage and markers of inflammation in a WM voxel have been previously reported in this cohort at age 11 years using proton magnetic resonance spectroscopy (Graham et al., 2020). Because the preadolescent brain is undergoing crucial synaptic pruning and myelination to increase neuronal efficiency (Deoni et al., 2015; Sakai, 2020), the observed WM changes may be a consequence of either delayed development or persistent HIV-related damage.

A number of the WM connections showed lower fNT and higher MD in the CPHIV group compared to the CHUU group, which may be an indication of complications in synaptic pruning in these fibers. These WM connections were predominantly to basal ganglia and thalamic nuclei. The internal capsule, which houses many thalamo-basal ganglia WM fibers, has been previously reported to show altered structural integrity in CPHIV (Sarma et al., 2014; Zhan et al., 2017; Hoare et al., 2018). In addition, we observed reduced nodal strength of the anterior, mediodorsal, and ventral anterior thalamic nuclei. Nodal strength demonstrates the density of the connections to a node (Rubinov and Sporns, 2010), and alterations in the structural nodal topology of the thalamus in the presence of HIV have been reported previously in older individuals (Bell et al., 2018; Aili et al., 2022). These results point to a possible link between our observed WM microstructural alterations and the strength of these thalamic nuclei within the structural network.

### Current study results in relation to this cohort at earlier ages

The results presented are from a cohort followed with neuroimaging since age 5. While previous work has not focused on the auditory system, our results contribute to the narrative of HIV-related changes presented at earlier ages. In particular, the regions we reported on connected to auditory regions have been identified at earlier ages and with other modalities. Affected WM connections to frontal GM areas, particularly to the lOFC, IFG (pars opercularis, pars triangularis, and pars orbitalis) and superior frontal gyrus, have been reported previously in the same cohort at age 7 (Madzime et al., 2021). At the same age, reduced functional connectivity to the IFG was identified (Toich et al., 2018). WM fibers from cortical and subcortical auditory regions to the IFG likely belong to the arcuate/uncinate fasciculus and the internal capsule (Maffei et al., 2015), both of which have been reported at previous ages in the same cohort at 5 and 7 years (Ackermann et al., 2016; Jankiewicz et al., 2017). In addition, Nwosu et al. (2021) also reported lower cortical thickness of frontal and temporo-insular regions at age 7 in these children. These results show similar areas are being reported across different ages and modalities but in the same cohort.

Nodal transitivity of the nucleus accumbens was higher in the CPHIV group compared to the CHUU group. Transitivity shows the connectedness among the nodes that anatomically link with the nucleus accumbens and thus the clustering or nodal segregation of the nodes that link to it. During neurodevelopment, nodal structural segregation in the structural brain network decreases as global integration increases (Bourne et al., 2016). Within the current cohort at age 5, we reported larger nucleus accumbens volumes in CPHIVs (Randall et al., 2017), suggesting earlier disruptions may contribute to the current results. The nucleus accumbens has also been reported to show altered subcortical volumes in other CPHIV cohorts (Yadav et al., 2017). The WM tract between this region and the left PAC additionally showed higher MD, AD, and fNT. MD and AD differences not only point to possible complications in WM myelination, but higher fNT also suggests possible delays in synaptic pruning of the fibers constituting this tract. Indeed, these microstructural alterations may contribute to higher transitivity.

Along with the putamen and caudate nucleus, the nucleus accumbens is an input nucleus in the basal ganglia. We reported several connections with higher MD from auditory structures such as the PAC that also included the putamen and the caudate. Previous work in the cohort has reported alterations to the putamen and the caudate, including putamen volumetric alterations at 5 and 7 years (Randall et al., 2017; Nwosu et al., 2018), as well as altered metabolism at age 9 in a voxel that included the caudate and the putamen (Robertson et al., 2018). There is growing interest in basal ganglia circuitry in relation to hearing and language processing, with evidence forming for an auditory cortico-striatal loop (Geiser et al., 2012; Lim et al., 2014). Our results suggest HIV vulnerabilities to basal ganglia nuclei throughout childhood in this cohort may affect communication from the CAS to the other parts of the cortex.

Identifying HIV-related WM alterations related to the central auditory system helps establish the viral effects on specific hearing-related parts of the brain. However, without links to functional measures of the auditory system, it is difficult to understand the consequences of these changes. Toward this goal, we investigated associations between structural integrity and neurocognitive functions that recruit the CAS. Among CPHIVs we found MD of six WM connections (IFOF, ILF, and internal capsule) was negatively associated with sequential processing (Figure 5). Sequential processing is a test of auditory working memory and is a summation of the subtests Word Order, Number Recall, and Hand Movement. These tasks recruit auditory–visual brain regions with hand movement (Kraus and Slater, 2015; Middlebrooks et al., 2016; Herbet et al., 2018). One connection between the left MGN and left ITC (internal capsule, Figure 5)—demonstrated a significant group interaction suggesting HIV infection may contribute to the variance of sequential processing abilities in this particular connection. Significantly lower scores in this domain were shown at younger ages of 7 and 9 (van Wyhe et al., 2021), and this finding may be related to earlier HIV effects.

However, caution must be taken in drawing a similar conclusion among the other associations. Even though the relationships were only significant in CPHIVs, there is a similarity of slopes across CPHIVs as well as CHUUs. As such, it is not possible to attribute these associations to HIV infection. The implicated WM connections are worth noting for future work in this cohort as it suggests the microstructural integrity of these tracts is related to auditory working memory across CPHIVs and potentially CHUUs.

## Future work

The current results warrant follow-up analyses at later ages. As group differences in sequential processing at earlier ages have been reported, longitudinal analyses that include both earlier and later time points are warranted. These analyses may provide a better understanding of the development of the CAS in the presence of HIV as well as its relationship with sequential processing measures. In addition, linking WM matter changes to functional MRI data may provide additional insight into functional consequences related to both the CAS and other networks. Finally, exploring the current results in relation to hearing outcomes is needed to answer the question of how altered WM may contribute to hearing abilities/deficits in this group of children.

## Conclusion

To our knowledge, this study is the first to investigate the microstructural integrity of the CAS and its interactions with other brain regions in CPHIVs. Our results show reduced WM maturation of the CAS as well as between the CAS and other brain regions. The altered network topology of hub regions that are structurally linked to auditory regions further suggests communication between the CAS and other parts of the brain is disrupted by HIV infection in children on treatment. Furthermore, microstructural alterations were associated with auditory working memory abilities. Taken together, our results suggest a microstructural vulnerability of the CAS WM and its structural links to language, motor, and visual processing regions in the presence of HIV. In addition, they are a first step in investigating the relationship between the CAS and the peripheral auditory system.

## Limitations

Even though manually tracing the auditory ROIs helped isolate WM specifically involved in acoustic processing, the reconstructions were variable among subjects.

The acoustic radiations are challenging to track because they are small, oriented transversally, and located in an area with a high density of crossing fibers. The classic DTI-based tensor model determines one main fiber orientation in each voxel and WM fibers belonging to the acoustic radiations may not necessarily be the largest contributing fibers to the fiber orientation in the voxels (Maffei et al., 2019).

In the structural network, we defined pair-wise connectivity as the fractional number of tracks (fNT)—a fraction obtained by dividing the number of streamlines that make up a WM bundle by the total number of reconstructed WM streamlines in the whole brain. Although this is a normalized proxy of bundle thickness it does not consider the anatomical variation in WM bundles in the brain resulting in some bundles being deemed as extremely small (abnormal) when they indeed, are normally smaller and propagate from small ROIs.

## Data availability statement

The original contributions presented in the study are included in the article/Supplementary material, further inquiries can be directed to the corresponding author.

## Ethics statement

The studies involving humans were approved by the Human Research Ethics Committees of the Universities of Cape Town and Stellenbosch. The studies were conducted in accordance with the local legislation and institutional requirements. Written informed consent for participation in this study was provided by the participants' legal guardians/next of kin.

## Author contributions

JM: Formal analysis, Methodology, Visualization, Writing – original draft. MJ: Conceptualization, Funding acquisition, Methodology, Resources, Supervision, Writing – review & editing. EM: Funding acquisition, Writing – review & editing. PT: Funding acquisition, Writing – review & editing. BL: Writing – review & editing, Funding acquisition. AK: Writing – review & editing, Conceptualization. MH: Supervision, Writing – original draft, Conceptualization, Methodology.
