# Supplementary material for: Reduced white matter maturation in the central auditory system of children living with HIV
Source: Front Neuroimaging. 2024 Mar 6;3:1341607. doi: 10.3389/fnimg.2024.1341607 (PMC10951401; doi:10.3389/fnimg.2024.1341607)
Supplement: Supplementary file 1 [file Table_1.docx]

## Appendices

### Appendix A: Automatically segmented regions of interest.

Table 4: 126 automatically segmented ROIs. R – right, L – left.

| ROI | ROI abbreviation |
| --- | --- |
| R-lateralorbitofrontal | R-lOFC |
| R-parsorbitalis | R-pars |
| R-frontalpole | R-FP |
| R-medialorbitofrontal | R-mOFC |
| R-parstriangularis | R-parstr |
| R-parsopercularis | R-parso |
| R-rostralmiddlefrontal | R-rMFC |
| R-superiorfrontal | R-SFC |
| R-caudalmiddlefrontal | R-cMFC |
| R-precentral | R-precentral |
| R-paracentral | R-paracentral |
| R-rostralanteriorcingulate | R-rACC |
| R-caudalanteriorcingulate | R-cACC |
| R-posteriorcingulate | R-PCC |
| R-isthmuscingulate | R-Isth |
| R-postcentral | R-postcentral |
| R-supramarginal | R-supramarginal |
| R-superiorparietal | R-SPC |
| R-inferiorparietal | R-IPC |
| R-precuneus | R-precuneus |
| R-cuneus | R-cuneus |
| R-pericalcarine | R-pericalc |
| R-lateraloccipital | R-LOC |
| R-lingual | R-lingual |
| R-fusiform | R-fusiform |
| R-parahippocampal | R-paraHippo |
| R-entorhinal | R-entorhinal |
| R-temporalpole | R-TP |
| R-inferiortemporal | R-ITC |
| R-middletemporal | R-MTC |
| R-bankssts | R-bankssts |
| R-superiortemporal | R-STC |
| R-transversetemporal | R-TTC |
| R-insula | R-insula |
| R-Pulvinar | R-Pulvinar |
| R-Anterior | R-Anterior |
| R-Medio_Dorsal | R-Medio_Dorsal |
| R-Ventral_Latero_Dorsal | R-VLD |
| R-Central_Lateral-Lateral_Posterior-Medial_Pulvinar | R-CLLPM_Pulvinar |
| R-Ventral_Anterior | R-Ventral_Anterior |
| R-Ventral_Latero_Ventral | R-VLV |
| R-Caudate | R-Caudate |
| R-Putamen | R-Putamen |
| R-Pallidum | R-Pallidum |
| R-Accumbens_area | R-Accumbens |
| R-Amygdala | R-Amygdala |
| R-Hippocampus | R-Hippo |
| R-Hippocampus_Parasubiculum | R-Hippo_Para |
| R-Hippocampus_Presubiculum | R-Hippo_Pres |
| R-Hippocampus_Subiculum | R-Hippo_Sub |
| R-Hippocampus_CA1 | R-Hippo_CA1 |
| R-Hippocampus_CA3 | R-Hippo_CA3 |
| R-Hippocampus_CA4 | R-Hippo_CA4 |
| R-Hippocampus_GCDG | R-Hippo_GCDG |
| R-Hippocampus_HATA | R-Hippo_HATA |
| R-Hippocampus_Fimbria | R-Hippo_Fimb |
| R-Hippocampus_Molecular_layer_HP | R-Hippo_Mol_layer_HP |
| R-Hippocampus_Hippocampal_fissure | R-Hippo_fissure |
| R-Hippocampus_Tail | R-Hippo_Tail |
| R-VentralDC | R-VDC |
| R-Hypothalamus | R-Hypothalamus |
| L-lateralorbitofrontal | L-lOFC |
| L-parsorbitalis | L-pars |
| L-frontalpole | L-FP |
| L-medialorbitofrontal | L-mOFC |
| L-parstriangularis | L-parstr |
| L-parsopercularis | L-parso |
| L-rostralmiddlefrontal | L-rMFC |
| L-superiorfrontal | L-SFC |
| L-caudalmiddlefrontal | L-cMFC |
| L-precentral | L-precentral |
| L-paracentral | L-paracentral |
| L-rostralanteriorcingulate | L-rACC |
| L-caudalanteriorcingulate | L-cACC |
| L-posteriorcingulate | L-PCC |
| L-isthmuscingulate | L-Isth |
| L-postcentral | L-postcentral |
| L-supramarginal | L-supramarginal |
| L-superiorparietal | L-SPC |
| L-inferiorparietal | L-IPC |
| L-precuneus | L-precuneus |
| L-cuneus | L-cuneus |
| L-pericalcarine | L-pericalc |
| L-lateraloccipital | L-LOC |
| L-lingual | L-lingual |
| L-fusiform | L-fusiform |
| L-parahippocampal | L-paraHippo |
| L-entorhinal | L-entorhinal |
| L-temporalpole | L-TP |
| L-inferiortemporal | L-ITC |
| L-middletemporal | L-MTC |
| L-bankssts | L-bankssts |
| L-superiortemporal | L-STC |
| L-transversetemporal | L-TTC |
| L-insula | L-insula |
| L-Pulvinar | L-Pulvinar |
| L-Anterior | L-Anterior |
| L-Medio_Dorsal | L-Medio_Dorsal |
| L-Ventral_Latero_Dorsal | L-VLD |
| L-Central_Lateral-Lateral_Posterior-Medial_Pulvinar | L-CLLPM_Pulvinar |
| L-Ventral_Anterior | L-Ventral_Anterior |
| L-Ventral_Latero_Ventral | L-VLV |
| L-Caudate | L-Caudate |
| L-Putamen | L-Putamen |
| L-Pallidum | L-Pallidum |
| L-Accumbens_area | L-Accumbens |
| L-Amygdala | L-Amygdala |
| L-Hippocampus | L-Hippo |
| L-Hippocampus_Parasubiculum | L-Hippo_Para |
| L-Hippocampus_Presubiculum | L-Hippo_Pres |
| L-Hippocampus_Subiculum | L-Hippo_Sub |
| L-Hippocampus_CA1 | L-Hippo_CA1 |
| L-Hippocampus_CA3 | L-Hippo_CA3 |
| L-Hippocampus_CA4 | L-Hippo_CA4 |
| L-Hippocampus_GCDG | L-Hippo_GCDG |
| L-Hippocampus_HATA | L-Hippo_HATA |
| L-Hippocampus_Fimbria | L-Hippo_Fimb |
| L-Hippocampus_Molecular_layer_HP | L-Hippo_Mol_layer_HP |
| L-Hippocampus_Hippocampal_fissure | L-Hippo_fissure |
| L-Hippocampus_Tail | L-Hippo_Tail |
| L-VentralDC | L-VDC |
| L-Hypothalamus | L-Hypothalamus |
| Brain_Stem-Midbrain | BS-mid |
| Brain_Stem-Pons | BS-pons |
| Brain_Stem-Medulla | BS-medulla |
| Brain_Stem-SCP | BS-SCP |

### Appendix B: DTI based tractography: CPHIV vs CHUU

Table 5: Standardized Beta (Std β), Standard Error (Std SE) values, p values and FDR corrected q values for the comparison between CHUU and CPHIV. Each row represents the relevant statistics for a WM connection and DTI measure. Thus each WM connection is represented in 6 rows for 6 DTI measures. L – left, R – right.

| **ROI1** | **ROI2** | **DTI measure** | **Std β** | **Std SE** | **p value** | **FDR q value** |
| --- | --- | --- | --- | --- | --- | --- |
| **L CN/SOC** | **L IC** | **AD** | 0.4312 | 0.0789 | <10^-4^ | 0.0001 |
| **L CN/SOC** | **L IC** | **FA** | 0.0352 | 0.0872 | 0.6873 | 0.8932 |
| **L CN/SOC** | **L IC** | **fNT** | 0.1341 | 0.0825 | 0.1063 | 0.2668 |
| **L CN/SOC** | **L IC** | **MD** | 0.3653 | 0.0808 | <10^-4^ | 0.0033 |
| **L CN/SOC** | **L IC** | **PV** | 0.1841 | 0.084 | 0.0301 | 0.2077 |
| **L CN/SOC** | **L IC** | **RD** | 0.2509 | 0.0839 | 0.0033 | 0.0188 |
| **L CN/SOC** | **L MGN** | **AD** | 0.291 | 0.0837 | 0.0007 | 0.0137 |
| **L CN/SOC** | **L MGN** | **FA** | 0.0853 | 0.0881 | 0.3349 | 0.7066 |
| **L CN/SOC** | **L MGN** | **fNT** | 0.1086 | 0.0889 | 0.2244 | 0.4247 |
| **L CN/SOC** | **L MGN** | **MD** | 0.2918 | 0.0837 | 0.0007 | 0.0077 |
| **L CN/SOC** | **L MGN** | **PV** | -0.0385 | -0.0858 | 0.6544 | 0.8946 |
| **L CN/SOC** | **L MGN** | **RD** | 0.2287 | 0.0849 | 0.0079 | 0.0256 |
| **L CN/SOC** | **L PAC** | **AD** | 0.3562 | 0.081 | <10^-4^ | 0.0046 |
| **L CN/SOC** | **L PAC** | **FA** | 0.1391 | 0.0871 | 0.1127 | 0.4128 |
| **L CN/SOC** | **L PAC** | **fNT** | 0.0604 | 0.0862 | 0.4851 | 0.7041 |
| **L CN/SOC** | **L PAC** | **MD** | 0.2803 | 0.0829 | 0.0009 | 0.0077 |
| **L CN/SOC** | **L PAC** | **PV** | 0.0757 | 0.0878 | 0.3903 | 0.7894 |
| **L CN/SOC** | **L PAC** | **RD** | 0.1732 | 0.0849 | 0.0433 | 0.0689 |
| **L CN/SOC** | **R IC** | **AD** | 0.3953 | 0.1146 | 0.001 | 0.0137 |
| **L CN/SOC** | **R IC** | **FA** | 0.1336 | 0.1189 | 0.265 | 0.6344 |
| **L CN/SOC** | **R IC** | **fNT** | 0.212 | 0.1131 | 0.0651 | 0.1929 |
| **L CN/SOC** | **R IC** | **MD** | 0.3799 | 0.114 | 0.0014 | 0.0082 |
| **L CN/SOC** | **R IC** | **PV** | 0.0561 | 0.1248 | 0.6549 | 0.8946 |
| **L CN/SOC** | **R IC** | **RD** | 0.2357 | 0.1172 | 0.0484 | 0.0741 |
| **L CN/SOC** | **R MGN** | **AD** | 0.312 | 0.1226 | 0.0134 | 0.0388 |
| **L CN/SOC** | **R MGN** | **FA** | -0.0821 | -0.1224 | 0.5047 | 0.8401 |
| **L CN/SOC** | **R MGN** | **fNT** | 0.2304 | 0.1084 | 0.0373 | 0.1337 |
| **L CN/SOC** | **R MGN** | **MD** | 0.2354 | 0.1178 | 0.05 | 0.0608 |
| **L CN/SOC** | **R MGN** | **PV** | -0.0157 | -0.1205 | 0.8966 | 0.9544 |
| **L CN/SOC** | **R MGN** | **RD** | 0.2843 | 0.1172 | 0.018 | 0.0382 |
| **L CN/SOC** | **R CN/SOC** | **AD** | 0.2841 | 0.0816 | 0.0007 | 0.0137 |
| **L CN/SOC** | **R CN/SOC** | **FA** | -0.0967 | -0.0854 | 0.2598 | 0.6286 |
| **L CN/SOC** | **R CN/SOC** | **fNT** | 0.1998 | 0.0845 | 0.0195 | 0.0866 |
| **L CN/SOC** | **R CN/SOC** | **MD** | 0.4171 | 0.078 | <10^-4^ | 0.0002 |
| **L CN/SOC** | **R CN/SOC** | **PV** | 0.2172 | 0.0833 | 0.0101 | 0.1511 |
| **L CN/SOC** | **R CN/SOC** | **RD** | 0.378 | 0.0792 | <10^-4^ | 0.0011 |
| **L IC** | **L PAC** | **AD** | 0.2843 | 0.1215 | 0.0224 | 0.0517 |
| **L IC** | **L PAC** | **FA** | -0.0636 | -0.1215 | 0.6022 | 0.8761 |
| **L IC** | **L PAC** | **fNT** | 0.0664 | 0.1186 | 0.5774 | 0.7767 |
| **L IC** | **L PAC** | **MD** | 0.3954 | 0.1129 | 0.0008 | 0.0077 |
| **L IC** | **L PAC** | **PV** | -0.0408 | -0.1245 | 0.7444 | 0.9405 |
| **L IC** | **L PAC** | **RD** | 0.3967 | 0.1115 | 0.0007 | 0.0091 |
| **L IC** | **R IC** | **AD** | 0.1385 | 0.1217 | 0.2594 | 0.3047 |
| **L IC** | **R IC** | **FA** | -0.0086 | -0.118 | 0.942 | 0.9804 |
| **L IC** | **R IC** | **fNT** | -0.2724 | -0.1173 | 0.0232 | 0.0955 |
| **L IC** | **R IC** | **MD** | 0.1915 | 0.1234 | 0.1257 | 0.1361 |
| **L IC** | **R IC** | **PV** | -0.2035 | -0.1248 | 0.1079 | 0.4071 |
| **L IC** | **R IC** | **RD** | 0.2309 | 0.1281 | 0.0763 | 0.1055 |
| **L IC** | **R CN/SOC** | **AD** | 0.2796 | 0.1265 | 0.0309 | 0.0637 |
| **L IC** | **R CN/SOC** | **FA** | -0.1744 | -0.1223 | 0.1584 | 0.5011 |
| **L IC** | **R CN/SOC** | **fNT** | -0.2529 | -0.1157 | 0.0323 | 0.1244 |
| **L IC** | **R CN/SOC** | **MD** | 0.3496 | 0.1162 | 0.0037 | 0.012 |
| **L IC** | **R CN/SOC** | **PV** | -0.0315 | -0.1223 | 0.7977 | 0.9526 |
| **L IC** | **R CN/SOC** | **RD** | 0.3766 | 0.1151 | 0.0017 | 0.0138 |
| **L MGN** | **L IC** | **AD** | 0.2959 | 0.1208 | 0.017 | 0.0444 |
| **L MGN** | **L IC** | **FA** | -0.166 | -0.1241 | 0.1855 | 0.5308 |
| **L MGN** | **L IC** | **fNT** | -0.1427 | -0.1269 | 0.2653 | 0.4701 |
| **L MGN** | **L IC** | **MD** | 0.3928 | 0.1143 | 0.001 | 0.0077 |
| **L MGN** | **L IC** | **PV** | -0.141 | -0.1219 | 0.2518 | 0.6583 |
| **L MGN** | **L IC** | **RD** | 0.4921 | 0.1095 | <10^-4^ | 0.0036 |
| **L MGN** | **L PAC** | **AD** | 0.3629 | 0.1176 | 0.003 | 0.0193 |
| **L MGN** | **L PAC** | **FA** | 0.002 | 0.1256 | 0.9872 | 0.9973 |
| **L MGN** | **L PAC** | **fNT** | 0.1071 | 0.1232 | 0.3876 | 0.5976 |
| **L MGN** | **L PAC** | **MD** | 0.403 | 0.1137 | 0.0007 | 0.0077 |
| **L MGN** | **L PAC** | **PV** | -0.0277 | -0.1131 | 0.8071 | 0.9544 |
| **L MGN** | **L PAC** | **RD** | 0.3771 | 0.1152 | 0.0017 | 0.0138 |
| **L MGN** | **R CN/SOC** | **AD** | 0.3289 | 0.1184 | 0.0071 | 0.0285 |
| **L MGN** | **R CN/SOC** | **FA** | 0.0895 | 0.1245 | 0.4744 | 0.8235 |
| **L MGN** | **R CN/SOC** | **fNT** | 0.2292 | 0.1268 | 0.0756 | 0.2128 |
| **L MGN** | **R CN/SOC** | **MD** | 0.31 | 0.1195 | 0.0117 | 0.0213 |
| **L MGN** | **R CN/SOC** | **PV** | 0.056 | 0.1236 | 0.6518 | 0.8946 |
| **L MGN** | **R CN/SOC** | **RD** | 0.2902 | 0.1193 | 0.0177 | 0.0382 |
| **R CN/SOC** | **R IC** | **AD** | 0.2793 | 0.1202 | 0.0232 | 0.0527 |
| **R CN/SOC** | **R IC** | **FA** | -0.0307 | -0.1225 | 0.8032 | 0.9434 |
| **R CN/SOC** | **R IC** | **fNT** | 0.1134 | 0.1249 | 0.3674 | 0.5772 |
| **R CN/SOC** | **R IC** | **MD** | 0.3867 | 0.1181 | 0.0017 | 0.0089 |
| **R CN/SOC** | **R IC** | **PV** | -0.0553 | -0.1255 | 0.6612 | 0.8947 |
| **R CN/SOC** | **R IC** | **RD** | 0.3297 | 0.1192 | 0.0074 | 0.0245 |
| **R CN/SOC** | **R MGN** | **AD** | 0.3605 | 0.1158 | 0.0027 | 0.0181 |
| **R CN/SOC** | **R MGN** | **FA** | -0.2434 | -0.1216 | 0.0497 | 0.2778 |
| **R CN/SOC** | **R MGN** | **fNT** | -0.1695 | -0.1222 | 0.1703 | 0.3533 |
| **R CN/SOC** | **R MGN** | **MD** | 0.4416 | 0.112 | 0.0002 | 0.0077 |
| **R CN/SOC** | **R MGN** | **PV** | -0.0992 | -0.1247 | 0.4289 | 0.8153 |
| **R CN/SOC** | **R MGN** | **RD** | 0.4726 | 0.1098 | 0.0001 | 0.004 |
| **R CN/SOC** | **R PAC** | **AD** | 0.2114 | 0.1223 | 0.0886 | 0.1253 |
| **R CN/SOC** | **R PAC** | **FA** | 0.1427 | 0.122 | 0.2462 | 0.6193 |
| **R CN/SOC** | **R PAC** | **fNT** | 0.1624 | 0.1191 | 0.1774 | 0.3636 |
| **R CN/SOC** | **R PAC** | **MD** | 0.1942 | 0.1241 | 0.1226 | 0.1336 |
| **R CN/SOC** | **R PAC** | **PV** | -0.2135 | -0.125 | 0.0925 | 0.3746 |
| **R CN/SOC** | **R PAC** | **RD** | 0.0734 | 0.1281 | 0.5687 | 0.6013 |
| **R IC** | **R PAC** | **AD** | 0.2446 | 0.1244 | 0.0536 | 0.0878 |
| **R IC** | **R PAC** | **FA** | -0.0928 | -0.1246 | 0.4593 | 0.8235 |
| **R IC** | **R PAC** | **fNT** | 0.2094 | 0.1162 | 0.0762 | 0.2132 |
| **R IC** | **R PAC** | **MD** | 0.3689 | 0.1163 | 0.0023 | 0.0101 |
| **R IC** | **R PAC** | **PV** | 0.1154 | 0.1251 | 0.3595 | 0.7586 |
| **R IC** | **R PAC** | **RD** | 0.3497 | 0.1179 | 0.0042 | 0.0205 |
| **R MGN** | **R IC** | **AD** | 0.3634 | 0.1166 | 0.0027 | 0.0181 |
| **R MGN** | **R IC** | **FA** | 0.2382 | 0.1224 | 0.056 | 0.2932 |
| **R MGN** | **R IC** | **fNT** | 0.2393 | 0.1344 | 0.0804 | 0.2165 |
| **R MGN** | **R IC** | **MD** | 0.2874 | 0.1189 | 0.0183 | 0.0287 |
| **R MGN** | **R IC** | **PV** | 0.1077 | 0.1246 | 0.3906 | 0.7894 |
| **R MGN** | **R IC** | **RD** | 0.2065 | 0.1213 | 0.0933 | 0.1252 |
| **R MGN** | **R PAC** | **AD** | 0.4617 | 0.1097 | 0.0001 | 0.0068 |
| **R MGN** | **R PAC** | **FA** | -0.1141 | -0.1264 | 0.37 | 0.7459 |
| **R MGN** | **R PAC** | **fNT** | 0.2517 | 0.1195 | 0.0392 | 0.1385 |
| **R MGN** | **R PAC** | **MD** | 0.4829 | 0.1135 | 0.0001 | 0.0061 |
| **R MGN** | **R PAC** | **PV** | 0.2136 | 0.1215 | 0.0834 | 0.3607 |
| **R MGN** | **R PAC** | **RD** | 0.3334 | 0.1182 | 0.0063 | 0.0234 |
| **L CN/SOC** | **L Anterior** | **AD** | 0.2792 | 0.1195 | 0.0225 | 0.0517 |
| **L CN/SOC** | **L Anterior** | **FA** | -0.0294 | -0.1236 | 0.8125 | 0.9442 |
| **L CN/SOC** | **L Anterior** | **fNT** | -0.3539 | -0.1199 | 0.0045 | 0.0347 |
| **L CN/SOC** | **L Anterior** | **MD** | 0.3587 | 0.1172 | 0.0032 | 0.0114 |
| **L CN/SOC** | **L Anterior** | **PV** | -0.4218 | -0.11 | 0.0003 | 0.0323 |
| **L CN/SOC** | **L Anterior** | **RD** | 0.2873 | 0.1197 | 0.0193 | 0.039 |
| **L CN/SOC** | **L Caudate** | **AD** | 0.3213 | 0.1201 | 0.0095 | 0.0311 |
| **L CN/SOC** | **L Caudate** | **FA** | -0.0467 | -0.1248 | 0.7095 | 0.8967 |
| **L CN/SOC** | **L Caudate** | **fNT** | 0.2102 | 0.1146 | 0.0712 | 0.2061 |
| **L CN/SOC** | **L Caudate** | **MD** | 0.3743 | 0.1169 | 0.0021 | 0.0097 |
| **L CN/SOC** | **L Caudate** | **PV** | -0.0605 | -0.1188 | 0.6123 | 0.8687 |
| **L CN/SOC** | **L Caudate** | **RD** | 0.3337 | 0.1183 | 0.0063 | 0.0234 |
| **L CN/SOC** | **L Hippo_CA1** | **AD** | 0.1539 | 0.1218 | 0.2106 | 0.2571 |
| **L CN/SOC** | **L Hippo_CA1** | **FA** | -0.0048 | -0.1247 | 0.9693 | 0.9892 |
| **L CN/SOC** | **L Hippo_CA1** | **fNT** | 0.0192 | 0.125 | 0.8782 | 0.9372 |
| **L CN/SOC** | **L Hippo_CA1** | **MD** | 0.1699 | 0.1226 | 0.1705 | 0.1798 |
| **L CN/SOC** | **L Hippo_CA1** | **PV** | -0.1305 | -0.1214 | 0.2862 | 0.6909 |
| **L CN/SOC** | **L Hippo_CA1** | **RD** | 0.1144 | 0.125 | 0.3632 | 0.3958 |
| **L CN/SOC** | **L Pallidum** | **AD** | 0.3421 | 0.117 | 0.0047 | 0.0221 |
| **L CN/SOC** | **L Pallidum** | **FA** | -0.0882 | -0.124 | 0.4796 | 0.8235 |
| **L CN/SOC** | **L Pallidum** | **fNT** | 0.1134 | 0.1114 | 0.3126 | 0.5188 |
| **L CN/SOC** | **L Pallidum** | **MD** | 0.3976 | 0.114 | 0.0009 | 0.0077 |
| **L CN/SOC** | **L Pallidum** | **PV** | -0.0738 | -0.1116 | 0.511 | 0.8461 |
| **L CN/SOC** | **L Pallidum** | **RD** | 0.3984 | 0.1138 | 0.0008 | 0.0096 |
| **L CN/SOC** | **L Putamen** | **AD** | 0.3947 | 0.1142 | 0.001 | 0.0137 |
| **L CN/SOC** | **L Putamen** | **FA** | 0.0213 | 0.1244 | 0.8645 | 0.963 |
| **L CN/SOC** | **L Putamen** | **fNT** | 0.236 | 0.1092 | 0.0342 | 0.1281 |
| **L CN/SOC** | **L Putamen** | **MD** | 0.4011 | 0.114 | 0.0008 | 0.0077 |
| **L CN/SOC** | **L Putamen** | **PV** | 0.0318 | 0.1182 | 0.7884 | 0.95 |
| **L CN/SOC** | **L Putamen** | **RD** | 0.3403 | 0.1172 | 0.005 | 0.0207 |
| **L CN/SOC** | **L rMFC** | **AD** | 0.3294 | 0.1227 | 0.0093 | 0.0308 |
| **L CN/SOC** | **L rMFC** | **FA** | 0.0564 | 0.128 | 0.6611 | 0.8932 |
| **L CN/SOC** | **L rMFC** | **fNT** | 0.1782 | 0.1255 | 0.1606 | 0.3419 |
| **L CN/SOC** | **L rMFC** | **MD** | 0.1919 | 0.1256 | 0.1315 | 0.1415 |
| **L CN/SOC** | **L rMFC** | **PV** | 0.017 | 0.128 | 0.8946 | 0.9544 |
| **L CN/SOC** | **L rMFC** | **RD** | 0.1082 | 0.1275 | 0.399 | 0.431 |
| **L CN/SOC** | **L SFC** | **AD** | 0.3267 | 0.1169 | 0.0068 | 0.0276 |
| **L CN/SOC** | **L SFC** | **FA** | -0.133 | -0.1229 | 0.2829 | 0.6615 |
| **L CN/SOC** | **L SFC** | **fNT** | 0.0377 | 0.116 | 0.746 | 0.8771 |
| **L CN/SOC** | **L SFC** | **MD** | 0.3892 | 0.1143 | 0.0011 | 0.0078 |
| **L CN/SOC** | **L SFC** | **PV** | 0.0788 | 0.114 | 0.4919 | 0.8446 |
| **L CN/SOC** | **L SFC** | **RD** | 0.3683 | 0.1156 | 0.0022 | 0.0153 |
| **L CN/SOC** | **L SPC** | **AD** | 0.3315 | 0.1172 | 0.0062 | 0.0268 |
| **L CN/SOC** | **L SPC** | **FA** | -0.0753 | -0.1238 | 0.5448 | 0.8635 |
| **L CN/SOC** | **L SPC** | **fNT** | -0.0062 | -0.124 | 0.9606 | 0.975 |
| **L CN/SOC** | **L SPC** | **MD** | 0.3619 | 0.1163 | 0.0027 | 0.0103 |
| **L CN/SOC** | **L SPC** | **PV** | -0.0838 | -0.1223 | 0.4956 | 0.8461 |
| **L CN/SOC** | **L SPC** | **RD** | 0.2918 | 0.1193 | 0.0171 | 0.0373 |
| **L CN/SOC** | **L VLD** | **AD** | 0.0996 | 0.1186 | 0.4037 | 0.4477 |
| **L CN/SOC** | **L VLD** | **FA** | -0.1745 | -0.1145 | 0.1322 | 0.4494 |
| **L CN/SOC** | **L VLD** | **fNT** | -0.1176 | -0.1226 | 0.3412 | 0.5517 |
| **L CN/SOC** | **L VLD** | **MD** | 0.3288 | 0.1173 | 0.0066 | 0.0153 |
| **L CN/SOC** | **L VLD** | **PV** | -0.3083 | -0.123 | 0.0148 | 0.1556 |
| **L CN/SOC** | **L VLD** | **RD** | 0.3702 | 0.1145 | 0.0019 | 0.0148 |
| **L CN/SOC** | **R Pallidum** | **AD** | 0.3463 | 0.1164 | 0.0041 | 0.0209 |
| **L CN/SOC** | **R Pallidum** | **FA** | 0.1465 | 0.1231 | 0.2383 | 0.608 |
| **L CN/SOC** | **R Pallidum** | **fNT** | 0.152 | 0.1194 | 0.2075 | 0.4074 |
| **L CN/SOC** | **R Pallidum** | **MD** | 0.3311 | 0.117 | 0.0061 | 0.0146 |
| **L CN/SOC** | **R Pallidum** | **PV** | 0.083 | 0.119 | 0.4882 | 0.8442 |
| **L CN/SOC** | **R Pallidum** | **RD** | 0.2392 | 0.1205 | 0.0512 | 0.0768 |
| **L CN/SOC** | **R Putamen** | **AD** | 0.3487 | 0.1158 | 0.0037 | 0.0208 |
| **L CN/SOC** | **R Putamen** | **FA** | 0.0738 | 0.1239 | 0.5535 | 0.8635 |
| **L CN/SOC** | **R Putamen** | **fNT** | 0.2071 | 0.1246 | 0.1018 | 0.2573 |
| **L CN/SOC** | **R Putamen** | **MD** | 0.1988 | 0.1207 | 0.1045 | 0.1159 |
| **L CN/SOC** | **R Putamen** | **PV** | 0.1974 | 0.1208 | 0.1069 | 0.4065 |
| **L CN/SOC** | **R Putamen** | **RD** | 0.2729 | 0.1197 | 0.0259 | 0.0479 |
| **L CN/SOC** | **R SFC** | **AD** | 0.3965 | 0.1141 | 0.0009 | 0.0137 |
| **L CN/SOC** | **R SFC** | **FA** | -0.0449 | -0.1228 | 0.7159 | 0.8979 |
| **L CN/SOC** | **R SFC** | **fNT** | -0.029 | -0.134 | 0.8183 | 0.9199 |
| **L CN/SOC** | **R SFC** | **MD** | 0.426 | 0.114 | 0.0004 | 0.0077 |
| **L CN/SOC** | **R SFC** | **PV** | -0.0678 | -0.1213 | 0.5781 | 0.8597 |
| **L CN/SOC** | **R SFC** | **RD** | 0.3711 | 0.117 | 0.0023 | 0.0155 |
| **L IC** | **L Pallidum** | **AD** | 0.2296 | 0.1206 | 0.0614 | 0.0952 |
| **L IC** | **L Pallidum** | **FA** | 0.0271 | 0.1235 | 0.8271 | 0.9513 |
| **L IC** | **L Pallidum** | **fNT** | -0.0625 | -0.1219 | 0.61 | 0.7962 |
| **L IC** | **L Pallidum** | **MD** | 0.2536 | 0.1182 | 0.0356 | 0.047 |
| **L IC** | **L Pallidum** | **PV** | -0.2584 | -0.1199 | 0.0348 | 0.2125 |
| **L IC** | **L Pallidum** | **RD** | 0.1932 | 0.1215 | 0.1167 | 0.1494 |
| **L IC** | **L Putamen** | **AD** | 0.2473 | 0.1139 | 0.0335 | 0.0668 |
| **L IC** | **L Putamen** | **FA** | -0.0187 | -0.1229 | 0.8798 | 0.968 |
| **L IC** | **L Putamen** | **fNT** | -0.0088 | -0.1194 | 0.9413 | 0.9637 |
| **L IC** | **L Putamen** | **MD** | 0.2933 | 0.1136 | 0.0121 | 0.0216 |
| **L IC** | **L Putamen** | **PV** | -0.1486 | -0.1245 | 0.2369 | 0.641 |
| **L IC** | **L Putamen** | **RD** | 0.2462 | 0.1188 | 0.0422 | 0.068 |
| **L IC** | **L SPC** | **AD** | 0.2764 | 0.1162 | 0.0202 | 0.0488 |
| **L IC** | **L SPC** | **FA** | -0.0656 | -0.1236 | 0.5972 | 0.8761 |
| **L IC** | **L SPC** | **fNT** | -0.0703 | -0.1235 | 0.5708 | 0.7717 |
| **L IC** | **L SPC** | **MD** | 0.2992 | 0.1169 | 0.0127 | 0.0224 |
| **L IC** | **L SPC** | **PV** | -0.1309 | -0.1215 | 0.2853 | 0.6909 |
| **L IC** | **L SPC** | **RD** | 0.2578 | 0.1197 | 0.0348 | 0.0597 |
| **L IC** | **L VLD** | **AD** | -0.0895 | -0.1234 | 0.4711 | 0.5123 |
| **L IC** | **L VLD** | **FA** | -0.3315 | -0.1144 | 0.0051 | 0.1639 |
| **L IC** | **L VLD** | **fNT** | -0.2748 | -0.1308 | 0.0401 | 0.1386 |
| **L IC** | **L VLD** | **MD** | 0.2207 | 0.1232 | 0.0778 | 0.0889 |
| **L IC** | **L VLD** | **PV** | -0.2223 | -0.1235 | 0.0769 | 0.3467 |
| **L IC** | **L VLD** | **RD** | 0.4802 | 0.1089 | <10^-4^ | 0.0037 |
| **L MGN** | **L Amygdala** | **AD** | 0.1601 | 0.1218 | 0.1932 | 0.2393 |
| **L MGN** | **L Amygdala** | **FA** | -0.0641 | -0.1218 | 0.6004 | 0.8761 |
| **L MGN** | **L Amygdala** | **fNT** | -0.1619 | -0.1227 | 0.1915 | 0.3846 |
| **L MGN** | **L Amygdala** | **MD** | 0.2185 | 0.12 | 0.0731 | 0.0841 |
| **L MGN** | **L Amygdala** | **PV** | -0.0367 | -0.1211 | 0.7627 | 0.9462 |
| **L MGN** | **L Amygdala** | **RD** | 0.1582 | 0.1209 | 0.1952 | 0.2282 |
| **L MGN** | **L Anterior** | **AD** | 0.1583 | 0.1226 | 0.201 | 0.2466 |
| **L MGN** | **L Anterior** | **FA** | -0.1677 | -0.1228 | 0.1765 | 0.5215 |
| **L MGN** | **L Anterior** | **fNT** | -0.4734 | -0.1073 | <10^-4^ | 0.0032 |
| **L MGN** | **L Anterior** | **MD** | 0.3589 | 0.1159 | 0.0029 | 0.0106 |
| **L MGN** | **L Anterior** | **PV** | -0.2943 | -0.1152 | 0.013 | 0.1529 |
| **L MGN** | **L Anterior** | **RD** | 0.3493 | 0.1168 | 0.0039 | 0.0197 |
| **L MGN** | **L Caudate** | **AD** | 0.2573 | 0.1201 | 0.0358 | 0.0699 |
| **L MGN** | **L Caudate** | **FA** | -0.0472 | -0.1243 | 0.7051 | 0.8967 |
| **L MGN** | **L Caudate** | **fNT** | -0.0274 | -0.1171 | 0.8159 | 0.9192 |
| **L MGN** | **L Caudate** | **MD** | 0.3406 | 0.1162 | 0.0046 | 0.0126 |
| **L MGN** | **L Caudate** | **PV** | 0.0686 | 0.1234 | 0.58 | 0.8597 |
| **L MGN** | **L Caudate** | **RD** | 0.319 | 0.1168 | 0.0081 | 0.0256 |
| **L MGN** | **L entorhinal** | **AD** | 0.293 | 0.1244 | 0.0217 | 0.051 |
| **L MGN** | **L entorhinal** | **FA** | -0.0896 | -0.1222 | 0.4663 | 0.8235 |
| **L MGN** | **L entorhinal** | **fNT** | -0.1835 | -0.1227 | 0.1397 | 0.3162 |
| **L MGN** | **L entorhinal** | **MD** | 0.3902 | 0.1126 | 0.0009 | 0.0077 |
| **L MGN** | **L entorhinal** | **PV** | -0.0426 | -0.1248 | 0.7341 | 0.9381 |
| **L MGN** | **L entorhinal** | **RD** | 0.3221 | 0.1153 | 0.0068 | 0.0241 |
| **L MGN** | **L fusiform** | **AD** | 0.2659 | 0.113 | 0.0216 | 0.051 |
| **L MGN** | **L fusiform** | **FA** | -0.085 | -0.1227 | 0.491 | 0.8326 |
| **L MGN** | **L fusiform** | **fNT** | -0.3499 | -0.12 | 0.0049 | 0.0362 |
| **L MGN** | **L fusiform** | **MD** | 0.3257 | 0.1135 | 0.0055 | 0.0137 |
| **L MGN** | **L fusiform** | **PV** | -0.1099 | -0.12 | 0.3632 | 0.7633 |
| **L MGN** | **L fusiform** | **RD** | 0.2388 | 0.1197 | 0.0501 | 0.0759 |
| **L MGN** | **L Hippo_CA1** | **AD** | -0.002 | -0.1238 | 0.9874 | 0.9893 |
| **L MGN** | **L Hippo_CA1** | **FA** | -0.0521 | -0.1172 | 0.6579 | 0.8932 |
| **L MGN** | **L Hippo_CA1** | **fNT** | -0.218 | -0.125 | 0.0751 | 0.2126 |
| **L MGN** | **L Hippo_CA1** | **MD** | 0.0309 | 0.1227 | 0.8019 | 0.8084 |
| **L MGN** | **L Hippo_CA1** | **PV** | -0.0973 | -0.1214 | 0.4257 | 0.8153 |
| **L MGN** | **L Hippo_CA1** | **RD** | 0.0402 | 0.1203 | 0.7393 | 0.7623 |
| **L MGN** | **L IPC** | **AD** | 0.354 | 0.1126 | 0.0025 | 0.0179 |
| **L MGN** | **L IPC** | **FA** | -0.2148 | -0.1292 | 0.1016 | 0.4082 |
| **L MGN** | **L IPC** | **fNT** | -0.1493 | -0.1245 | 0.2346 | 0.4359 |
| **L MGN** | **L IPC** | **MD** | 0.391 | 0.1133 | 0.001 | 0.0077 |
| **L MGN** | **L IPC** | **PV** | -0.1799 | -0.1197 | 0.1375 | 0.4724 |
| **L MGN** | **L IPC** | **RD** | 0.3557 | 0.1167 | 0.0033 | 0.0188 |
| **L MGN** | **L ITC** | **AD** | 0.179 | 0.1159 | 0.1272 | 0.168 |
| **L MGN** | **L ITC** | **FA** | -0.1574 | -0.1195 | 0.1921 | 0.54 |
| **L MGN** | **L ITC** | **fNT** | -0.0947 | -0.112 | 0.4458 | 0.6646 |
| **L MGN** | **L ITC** | **MD** | 0.2929 | 0.1157 | 0.0137 | 0.0235 |
| **L MGN** | **L ITC** | **PV** | -0.0646 | -0.1185 | 0.5872 | 0.8651 |
| **L MGN** | **L ITC** | **RD** | 0.2555 | 0.1246 | 0.0445 | 0.0703 |
| **L MGN** | **L lingual** | **AD** | 0.2477 | 0.123 | 0.0482 | 0.0823 |
| **L MGN** | **L lingual** | **FA** | 0.0753 | 0.1254 | 0.5504 | 0.8635 |
| **L MGN** | **L lingual** | **fNT** | -0.1694 | -0.1164 | 0.1506 | 0.3284 |
| **L MGN** | **L lingual** | **MD** | 0.22 | 0.1202 | 0.0718 | 0.0828 |
| **L MGN** | **L lingual** | **PV** | 0.1073 | 0.1243 | 0.3911 | 0.7894 |
| **L MGN** | **L lingual** | **RD** | 0.112 | 0.1222 | 0.3628 | 0.3958 |
| **L MGN** | **L LOC** | **AD** | 0.3645 | 0.1136 | 0.0021 | 0.0173 |
| **L MGN** | **L LOC** | **FA** | -0.0813 | -0.1243 | 0.5157 | 0.845 |
| **L MGN** | **L LOC** | **fNT** | -0.0243 | -0.116 | 0.8465 | 0.9372 |
| **L MGN** | **L LOC** | **MD** | 0.2355 | 0.1152 | 0.0449 | 0.0557 |
| **L MGN** | **L LOC** | **PV** | -0.0696 | -0.1182 | 0.5581 | 0.8552 |
| **L MGN** | **L LOC** | **RD** | 0.196 | 0.119 | 0.1043 | 0.1367 |
| **L MGN** | **L Pallidum** | **AD** | 0.2737 | 0.1191 | 0.0247 | 0.0547 |
| **L MGN** | **L Pallidum** | **FA** | -0.0516 | -0.1234 | 0.6771 | 0.8932 |
| **L MGN** | **L Pallidum** | **fNT** | -0.1723 | -0.1172 | 0.1461 | 0.3205 |
| **L MGN** | **L Pallidum** | **MD** | 0.2545 | 0.1177 | 0.0341 | 0.0458 |
| **L MGN** | **L Pallidum** | **PV** | -0.1817 | -0.1129 | 0.1123 | 0.4141 |
| **L MGN** | **L Pallidum** | **RD** | 0.2247 | 0.1174 | 0.0599 | 0.0872 |
| **L MGN** | **L precuneus** | **AD** | 0.312 | 0.1162 | 0.0092 | 0.0308 |
| **L MGN** | **L precuneus** | **FA** | -0.0442 | -0.1238 | 0.722 | 0.9007 |
| **L MGN** | **L precuneus** | **fNT** | 0.2297 | 0.1213 | 0.0628 | 0.1872 |
| **L MGN** | **L precuneus** | **MD** | 0.3515 | 0.1156 | 0.0034 | 0.0119 |
| **L MGN** | **L precuneus** | **PV** | 0.2809 | 0.1215 | 0.024 | 0.1968 |
| **L MGN** | **L precuneus** | **RD** | 0.2646 | 0.1201 | 0.0311 | 0.0548 |
| **L MGN** | **L Putamen** | **AD** | 0.2731 | 0.1172 | 0.0229 | 0.0523 |
| **L MGN** | **L Putamen** | **FA** | -0.0507 | -0.1238 | 0.6834 | 0.8932 |
| **L MGN** | **L Putamen** | **fNT** | -0.0399 | -0.1186 | 0.7375 | 0.8731 |
| **L MGN** | **L Putamen** | **MD** | 0.2849 | 0.1152 | 0.0159 | 0.0258 |
| **L MGN** | **L Putamen** | **PV** | -0.0332 | -0.1169 | 0.7772 | 0.9487 |
| **L MGN** | **L Putamen** | **RD** | 0.2463 | 0.1164 | 0.0381 | 0.0628 |
| **L MGN** | **L rMFC** | **AD** | 0.162 | 0.125 | 0.1994 | 0.2452 |
| **L MGN** | **L rMFC** | **FA** | -0.0565 | -0.1279 | 0.6605 | 0.8932 |
| **L MGN** | **L rMFC** | **fNT** | 0.0745 | 0.1272 | 0.5602 | 0.7652 |
| **L MGN** | **L rMFC** | **MD** | 0.2038 | 0.1256 | 0.1095 | 0.1204 |
| **L MGN** | **L rMFC** | **PV** | -0.1662 | -0.1272 | 0.1962 | 0.5922 |
| **L MGN** | **L rMFC** | **RD** | 0.1589 | 0.1275 | 0.2172 | 0.251 |
| **L MGN** | **L SFC** | **AD** | 0.3304 | 0.1176 | 0.0065 | 0.0272 |
| **L MGN** | **L SFC** | **FA** | -0.0571 | -0.1213 | 0.6391 | 0.8932 |
| **L MGN** | **L SFC** | **fNT** | -0.2024 | -0.1216 | 0.1007 | 0.2559 |
| **L MGN** | **L SFC** | **MD** | 0.3375 | 0.1163 | 0.005 | 0.0132 |
| **L MGN** | **L SFC** | **PV** | -0.0219 | -0.1236 | 0.86 | 0.9544 |
| **L MGN** | **L SFC** | **RD** | 0.2733 | 0.1171 | 0.0226 | 0.0435 |
| **L MGN** | **L SPC** | **AD** | 0.2667 | 0.114 | 0.0223 | 0.0517 |
| **L MGN** | **L SPC** | **FA** | -0.2118 | -0.1208 | 0.0842 | 0.3679 |
| **L MGN** | **L SPC** | **fNT** | -0.1666 | -0.1229 | 0.1798 | 0.364 |
| **L MGN** | **L SPC** | **MD** | 0.3134 | 0.1146 | 0.008 | 0.0174 |
| **L MGN** | **L SPC** | **PV** | -0.0343 | -0.1151 | 0.7667 | 0.9462 |
| **L MGN** | **L SPC** | **RD** | 0.3066 | 0.1167 | 0.0107 | 0.0298 |
| **L MGN** | **L TP** | **AD** | 0.1673 | 0.1207 | 0.1705 | 0.2166 |
| **L MGN** | **L TP** | **FA** | -0.1479 | -0.1207 | 0.2248 | 0.5918 |
| **L MGN** | **L TP** | **fNT** | 0.0224 | 0.120 | 0.8574 | 0.9372 |
| **L MGN** | **L TP** | **MD** | 0.3001 | 0.119 | 0.0141 | 0.0236 |
| **L MGN** | **L TP** | **PV** | -0.0451 | -0.1229 | 0.7148 | 0.9319 |
| **L MGN** | **L TP** | **RD** | 0.2587 | 0.1198 | 0.0344 | 0.0593 |
| **L MGN** | **L Ventral_Anterior** | **AD** | -0.1266 | -0.1227 | 0.3059 | 0.3534 |
| **L MGN** | **L Ventral_Anterior** | **FA** | -0.2899 | -0.1189 | 0.0174 | 0.1809 |
| **L MGN** | **L Ventral_Anterior** | **fNT** | 0.1807 | 0.1219 | 0.1429 | 0.3176 |
| **L MGN** | **L Ventral_Anterior** | **MD** | 0.147 | 0.1227 | 0.2352 | 0.2466 |
| **L MGN** | **L Ventral_Anterior** | **PV** | -0.303 | -0.1147 | 0.0103 | 0.1511 |
| **L MGN** | **L Ventral_Anterior** | **RD** | 0.334 | 0.1177 | 0.006 | 0.023 |
| **L MGN** | **L VLD** | **AD** | -0.0341 | -0.1239 | 0.7842 | 0.8035 |
| **L MGN** | **L VLD** | **FA** | -0.1958 | -0.1203 | 0.1081 | 0.4108 |
| **L MGN** | **L VLD** | **fNT** | -0.0971 | -0.1232 | 0.4332 | 0.6498 |
| **L MGN** | **L VLD** | **MD** | 0.2435 | 0.1214 | 0.0489 | 0.0597 |
| **L MGN** | **L VLD** | **PV** | -0.3436 | -0.1162 | 0.0043 | 0.1056 |
| **L MGN** | **L VLD** | **RD** | 0.2961 | 0.1169 | 0.0136 | 0.0326 |
| **L MGN** | **R Pallidum** | **AD** | 0.2669 | 0.1275 | 0.0405 | 0.0747 |
| **L MGN** | **R Pallidum** | **FA** | -0.0124 | -0.1277 | 0.9229 | 0.9804 |
| **L MGN** | **R Pallidum** | **fNT** | -0.1217 | -0.1281 | 0.3456 | 0.5569 |
| **L MGN** | **R Pallidum** | **MD** | 0.3495 | 0.1248 | 0.0069 | 0.0156 |
| **L MGN** | **R Pallidum** | **PV** | -0.1047 | -0.1258 | 0.4086 | 0.801 |
| **L MGN** | **R Pallidum** | **RD** | 0.3416 | 0.1245 | 0.0081 | 0.0256 |
| **L PAC** | **L Accumbens** | **AD** | 0.3644 | 0.1162 | 0.0026 | 0.0179 |
| **L PAC** | **L Accumbens** | **FA** | 0.1082 | 0.1231 | 0.3824 | 0.7565 |
| **L PAC** | **L Accumbens** | **fNT** | 0.3814 | 0.1249 | 0.0035 | 0.0292 |
| **L PAC** | **L Accumbens** | **MD** | 0.3889 | 0.1134 | 0.0011 | 0.0077 |
| **L PAC** | **L Accumbens** | **PV** | 0.3003 | 0.1143 | 0.0107 | 0.1511 |
| **L PAC** | **L Accumbens** | **RD** | 0.2573 | 0.1185 | 0.0336 | 0.0584 |
| **L PAC** | **L Amygdala** | **AD** | 0.2659 | 0.1195 | 0.0295 | 0.0619 |
| **L PAC** | **L Amygdala** | **FA** | -0.2783 | -0.1207 | 0.0244 | 0.1926 |
| **L PAC** | **L Amygdala** | **fNT** | 0.071 | 0.118 | 0.5497 | 0.76 |
| **L PAC** | **L Amygdala** | **MD** | 0.3115 | 0.1161 | 0.0092 | 0.0185 |
| **L PAC** | **L Amygdala** | **PV** | 0.0158 | 0.1226 | 0.8982 | 0.9544 |
| **L PAC** | **L Amygdala** | **RD** | 0.2824 | 0.1163 | 0.0179 | 0.0382 |
| **L PAC** | **L Anterior** | **AD** | 0.3805 | 0.1147 | 0.0015 | 0.0155 |
| **L PAC** | **L Anterior** | **FA** | -0.2483 | -0.1193 | 0.0412 | 0.2565 |
| **L PAC** | **L Anterior** | **fNT** | -0.1773 | -0.1221 | 0.151 | 0.3284 |
| **L PAC** | **L Anterior** | **MD** | 0.461 | 0.1096 | 0.0001 | 0.0061 |
| **L PAC** | **L Anterior** | **PV** | -0.1184 | -0.1232 | 0.3401 | 0.7301 |
| **L PAC** | **L Anterior** | **RD** | 0.4322 | 0.1113 | 0.0002 | 0.006 |
| **L PAC** | **L Caudate** | **AD** | 0.2566 | 0.1204 | 0.0367 | 0.0709 |
| **L PAC** | **L Caudate** | **FA** | -0.1528 | -0.1229 | 0.2181 | 0.584 |
| **L PAC** | **L Caudate** | **fNT** | 0.1781 | 0.12 | 0.1425 | 0.3176 |
| **L PAC** | **L Caudate** | **MD** | 0.3232 | 0.1179 | 0.0079 | 0.0172 |
| **L PAC** | **L Caudate** | **PV** | 0.0719 | 0.1215 | 0.556 | 0.8552 |
| **L PAC** | **L Caudate** | **RD** | 0.3033 | 0.1188 | 0.0129 | 0.0325 |
| **L PAC** | **L fusiform** | **AD** | 0.2428 | 0.1164 | 0.0408 | 0.0749 |
| **L PAC** | **L fusiform** | **FA** | -0.1379 | -0.1214 | 0.26 | 0.6286 |
| **L PAC** | **L fusiform** | **fNT** | -0.0188 | -0.113 | 0.8788 | 0.9372 |
| **L PAC** | **L fusiform** | **MD** | 0.3018 | 0.113 | 0.0095 | 0.0186 |
| **L PAC** | **L fusiform** | **PV** | 0.0213 | 0.1233 | 0.8631 | 0.9544 |
| **L PAC** | **L fusiform** | **RD** | 0.3342 | 0.1153 | 0.0051 | 0.0207 |
| **L PAC** | **L Hippo_CA1** | **AD** | 0.1104 | 0.1236 | 0.3752 | 0.4199 |
| **L PAC** | **L Hippo_CA1** | **FA** | -0.2511 | -0.1194 | 0.0393 | 0.251 |
| **L PAC** | **L Hippo_CA1** | **fNT** | -0.0867 | -0.1176 | 0.4636 | 0.6811 |
| **L PAC** | **L Hippo_CA1** | **MD** | 0.2523 | 0.1242 | 0.0465 | 0.0575 |
| **L PAC** | **L Hippo_CA1** | **PV** | -0.0898 | -0.1246 | 0.4738 | 0.8412 |
| **L PAC** | **L Hippo_CA1** | **RD** | 0.243 | 0.1233 | 0.0532 | 0.0793 |
| **L PAC** | **L Hippo_HATA** | **AD** | 0.0082 | 0.1225 | 0.9468 | 0.9564 |
| **L PAC** | **L Hippo_HATA** | **FA** | -0.1909 | -0.1243 | 0.1296 | 0.4494 |
| **L PAC** | **L Hippo_HATA** | **fNT** | 0.0422 | 0.123 | 0.7342 | 0.8731 |
| **L PAC** | **L Hippo_HATA** | **MD** | 0.1947 | 0.1223 | 0.1161 | 0.1274 |
| **L PAC** | **L Hippo_HATA** | **PV** | 0.0394 | 0.1255 | 0.7544 | 0.9405 |
| **L PAC** | **L Hippo_HATA** | **RD** | 0.2006 | 0.1252 | 0.114 | 0.1467 |
| **L PAC** | **L IPC** | **AD** | 0.2189 | 0.1185 | 0.0692 | 0.1044 |
| **L PAC** | **L IPC** | **FA** | -0.244 | -0.1207 | 0.0472 | 0.2702 |
| **L PAC** | **L IPC** | **fNT** | 0.0187 | 0.1243 | 0.881 | 0.9374 |
| **L PAC** | **L IPC** | **MD** | 0.3257 | 0.1163 | 0.0067 | 0.0153 |
| **L PAC** | **L IPC** | **PV** | 0.1319 | 0.1179 | 0.2676 | 0.6764 |
| **L PAC** | **L IPC** | **RD** | 0.333 | 0.1171 | 0.0059 | 0.023 |
| **L PAC** | **L ITC** | **AD** | 0.3692 | 0.1096 | 0.0013 | 0.0144 |
| **L PAC** | **L ITC** | **FA** | 0.0115 | 0.1242 | 0.9266 | 0.9804 |
| **L PAC** | **L ITC** | **fNT** | -0.2894 | -0.121 | 0.0197 | 0.0868 |
| **L PAC** | **L ITC** | **MD** | 0.308 | 0.114 | 0.0087 | 0.0179 |
| **L PAC** | **L ITC** | **PV** | -0.0641 | -0.1226 | 0.6027 | 0.8652 |
| **L PAC** | **L ITC** | **RD** | 0.294 | 0.1183 | 0.0156 | 0.0351 |
| **L PAC** | **L LOC** | **AD** | 0.158 | 0.1176 | 0.1838 | 0.23 |
| **L PAC** | **L LOC** | **FA** | -0.262 | -0.1239 | 0.0384 | 0.249 |
| **L PAC** | **L LOC** | **fNT** | 0.1181 | 0.1231 | 0.3409 | 0.5517 |
| **L PAC** | **L LOC** | **MD** | 0.3129 | 0.1164 | 0.0091 | 0.0185 |
| **L PAC** | **L LOC** | **PV** | 0.1575 | 0.1246 | 0.2107 | 0.6104 |
| **L PAC** | **L LOC** | **RD** | 0.3738 | 0.1207 | 0.0029 | 0.0186 |
| **L PAC** | **L lOFC** | **AD** | 0.3752 | 0.114 | 0.0016 | 0.0156 |
| **L PAC** | **L lOFC** | **FA** | 0.112 | 0.1231 | 0.3664 | 0.7459 |
| **L PAC** | **L lOFC** | **fNT** | -0.3761 | -0.1207 | 0.0028 | 0.0251 |
| **L PAC** | **L lOFC** | **MD** | 0.2967 | 0.116 | 0.0128 | 0.0225 |
| **L PAC** | **L lOFC** | **PV** | -0.181 | -0.1204 | 0.1374 | 0.4724 |
| **L PAC** | **L lOFC** | **RD** | 0.2497 | 0.1223 | 0.0452 | 0.071 |
| **L PAC** | **L Pallidum** | **AD** | 0.2472 | 0.1194 | 0.0423 | 0.076 |
| **L PAC** | **L Pallidum** | **FA** | -0.0723 | -0.123 | 0.5587 | 0.8635 |
| **L PAC** | **L Pallidum** | **fNT** | -0.4515 | -0.1115 | 0.0001 | 0.0053 |
| **L PAC** | **L Pallidum** | **MD** | 0.2442 | 0.1189 | 0.0438 | 0.0546 |
| **L PAC** | **L Pallidum** | **PV** | -0.1775 | -0.1164 | 0.1319 | 0.4595 |
| **L PAC** | **L Pallidum** | **RD** | 0.2035 | 0.1199 | 0.0941 | 0.126 |
| **L PAC** | **L parso** | **AD** | 0.3927 | 0.1096 | 0.0006 | 0.0137 |
| **L PAC** | **L parso** | **FA** | 0.0234 | 0.1238 | 0.8505 | 0.9626 |
| **L PAC** | **L parso** | **fNT** | -0.0727 | -0.1218 | 0.5524 | 0.76 |
| **L PAC** | **L parso** | **MD** | 0.3331 | 0.1153 | 0.0052 | 0.0133 |
| **L PAC** | **L parso** | **PV** | -0.0927 | -0.1213 | 0.4475 | 0.8379 |
| **L PAC** | **L parso** | **RD** | 0.2054 | 0.1214 | 0.0954 | 0.1273 |
| **L PAC** | **L parstr** | **AD** | 0.3019 | 0.1166 | 0.0118 | 0.0355 |
| **L PAC** | **L parstr** | **FA** | -0.0996 | -0.1259 | 0.4314 | 0.8047 |
| **L PAC** | **L parstr** | **fNT** | -0.1539 | -0.1244 | 0.2202 | 0.4214 |
| **L PAC** | **L parstr** | **MD** | 0.3558 | 0.1143 | 0.0027 | 0.0103 |
| **L PAC** | **L parstr** | **PV** | -0.1281 | -0.1239 | 0.3049 | 0.7039 |
| **L PAC** | **L parstr** | **RD** | 0.3095 | 0.1182 | 0.0109 | 0.0301 |
| **L PAC** | **L Putamen** | **AD** | 0.3587 | 0.1148 | 0.0026 | 0.0179 |
| **L PAC** | **L Putamen** | **FA** | -0.0716 | -0.1238 | 0.565 | 0.8635 |
| **L PAC** | **L Putamen** | **fNT** | -0.2891 | -0.1188 | 0.0177 | 0.0825 |
| **L PAC** | **L Putamen** | **MD** | 0.3524 | 0.1145 | 0.003 | 0.0109 |
| **L PAC** | **L Putamen** | **PV** | -0.1293 | -0.1181 | 0.2776 | 0.6845 |
| **L PAC** | **L Putamen** | **RD** | 0.284 | 0.1178 | 0.0187 | 0.0388 |
| **L PAC** | **L rMFC** | **AD** | 0.2909 | 0.1191 | 0.0172 | 0.0444 |
| **L PAC** | **L rMFC** | **FA** | -0.0417 | -0.1237 | 0.7372 | 0.9082 |
| **L PAC** | **L rMFC** | **fNT** | -0.0828 | -0.1239 | 0.5063 | 0.7163 |
| **L PAC** | **L rMFC** | **MD** | 0.2986 | 0.1185 | 0.0141 | 0.0237 |
| **L PAC** | **L rMFC** | **PV** | 0.0681 | 0.1232 | 0.5825 | 0.8608 |
| **L PAC** | **L rMFC** | **RD** | 0.2397 | 0.1204 | 0.0505 | 0.076 |
| **L PAC** | **L SFC** | **AD** | 0.3674 | 0.115 | 0.0022 | 0.0173 |
| **L PAC** | **L SFC** | **FA** | -0.0644 | -0.1234 | 0.6035 | 0.8761 |
| **L PAC** | **L SFC** | **fNT** | -0.2656 | -0.1173 | 0.0268 | 0.1061 |
| **L PAC** | **L SFC** | **MD** | 0.343 | 0.1158 | 0.0042 | 0.0121 |
| **L PAC** | **L SFC** | **PV** | -0.0486 | -0.1232 | 0.6947 | 0.9291 |
| **L PAC** | **L SFC** | **RD** | 0.2534 | 0.1194 | 0.0375 | 0.0623 |
| **L PAC** | **L SPC** | **AD** | 0.3289 | 0.1174 | 0.0067 | 0.0275 |
| **L PAC** | **L SPC** | **FA** | -0.1159 | -0.1235 | 0.3516 | 0.7327 |
| **L PAC** | **L SPC** | **fNT** | -0.1679 | -0.1205 | 0.168 | 0.3533 |
| **L PAC** | **L SPC** | **MD** | 0.3473 | 0.1143 | 0.0034 | 0.0119 |
| **L PAC** | **L SPC** | **PV** | 0.0637 | 0.1226 | 0.6049 | 0.8652 |
| **L PAC** | **L SPC** | **RD** | 0.3007 | 0.1174 | 0.0127 | 0.0325 |
| **L PAC** | **L TP** | **AD** | 0.2618 | 0.1196 | 0.0322 | 0.0649 |
| **L PAC** | **L TP** | **FA** | -0.0978 | -0.1234 | 0.4309 | 0.8047 |
| **L PAC** | **L TP** | **fNT** | 0.0187 | 0.1223 | 0.8786 | 0.9372 |
| **L PAC** | **L TP** | **MD** | 0.3337 | 0.1154 | 0.0052 | 0.0133 |
| **L PAC** | **L TP** | **PV** | -0.0305 | -0.1212 | 0.8021 | 0.9537 |
| **L PAC** | **L TP** | **RD** | 0.2936 | 0.1175 | 0.0149 | 0.0338 |
| **L PAC** | **L Ventral_Anterior** | **AD** | 0.3554 | 0.1168 | 0.0034 | 0.0204 |
| **L PAC** | **L Ventral_Anterior** | **FA** | -0.1051 | -0.1225 | 0.394 | 0.7665 |
| **L PAC** | **L Ventral_Anterior** | **fNT** | 0.1858 | 0.1288 | 0.1543 | 0.3325 |
| **L PAC** | **L Ventral_Anterior** | **MD** | 0.382 | 0.1144 | 0.0014 | 0.0082 |
| **L PAC** | **L Ventral_Anterior** | **PV** | -0.0704 | -0.1237 | 0.5711 | 0.8597 |
| **L PAC** | **L Ventral_Anterior** | **RD** | 0.3548 | 0.1157 | 0.0031 | 0.0187 |
| **L PAC** | **L VLD** | **AD** | 0.2921 | 0.1192 | 0.0169 | 0.0444 |
| **L PAC** | **L VLD** | **FA** | 0.0102 | 0.1236 | 0.9346 | 0.9804 |
| **L PAC** | **L VLD** | **fNT** | 0.1256 | 0.1224 | 0.3087 | 0.516 |
| **L PAC** | **L VLD** | **MD** | 0.3459 | 0.1165 | 0.0041 | 0.0121 |
| **L PAC** | **L VLD** | **PV** | -0.12 | -0.1228 | 0.3318 | 0.7229 |
| **L PAC** | **L VLD** | **RD** | 0.2772 | 0.1186 | 0.0224 | 0.0432 |
| **L PAC** | **R Pallidum** | **AD** | 0.3021 | 0.1229 | 0.0167 | 0.0443 |
| **L PAC** | **R Pallidum** | **FA** | 0.0232 | 0.1288 | 0.8576 | 0.963 |
| **L PAC** | **R Pallidum** | **fNT** | -0.0656 | -0.1134 | 0.6123 | 0.7962 |
| **L PAC** | **R Pallidum** | **MD** | 0.2845 | 0.1222 | 0.0231 | 0.0338 |
| **L PAC** | **R Pallidum** | **PV** | 0.0271 | 0.1288 | 0.8338 | 0.9544 |
| **L PAC** | **R Pallidum** | **RD** | 0.1882 | 0.1251 | 0.1375 | 0.1712 |
| **L PAC** | **R Putamen** | **AD** | 0.23 | 0.1227 | 0.0653 | 0.1 |
| **L PAC** | **R Putamen** | **FA** | -0.0193 | -0.1261 | 0.8787 | 0.968 |
| **L PAC** | **R Putamen** | **fNT** | -0.0745 | -0.1262 | 0.5569 | 0.764 |
| **L PAC** | **R Putamen** | **MD** | 0.2444 | 0.1211 | 0.0477 | 0.0586 |
| **L PAC** | **R Putamen** | **PV** | -0.0133 | -0.1267 | 0.9165 | 0.9544 |
| **L PAC** | **R Putamen** | **RD** | 0.172 | 0.1233 | 0.1678 | 0.2023 |
| **R CN/SOC** | **L Caudate** | **AD** | 0.1718 | 0.1238 | 0.1701 | 0.2166 |
| **R CN/SOC** | **L Caudate** | **FA** | -0.0099 | -0.1256 | 0.9375 | 0.9804 |
| **R CN/SOC** | **L Caudate** | **fNT** | 0.1209 | 0.1215 | 0.3289 | 0.5402 |
| **R CN/SOC** | **L Caudate** | **MD** | 0.252 | 0.1218 | 0.0426 | 0.0533 |
| **R CN/SOC** | **L Caudate** | **PV** | -0.0679 | -0.1189 | 0.5702 | 0.8597 |
| **R CN/SOC** | **L Caudate** | **RD** | 0.2593 | 0.1216 | 0.0367 | 0.0617 |
| **R CN/SOC** | **L Pallidum** | **AD** | 0.2621 | 0.12 | 0.0324 | 0.065 |
| **R CN/SOC** | **L Pallidum** | **FA** | -0.0593 | -0.1222 | 0.6291 | 0.8927 |
| **R CN/SOC** | **L Pallidum** | **fNT** | 0.3319 | 0.1194 | 0.0072 | 0.0459 |
| **R CN/SOC** | **L Pallidum** | **MD** | 0.1982 | 0.1221 | 0.1093 | 0.1204 |
| **R CN/SOC** | **L Pallidum** | **PV** | 0.0228 | 0.1184 | 0.8481 | 0.9544 |
| **R CN/SOC** | **L Pallidum** | **RD** | 0.2827 | 0.1185 | 0.0199 | 0.04 |
| **R CN/SOC** | **L Putamen** | **AD** | 0.252 | 0.1202 | 0.0399 | 0.0742 |
| **R CN/SOC** | **L Putamen** | **FA** | 0.0304 | 0.1232 | 0.806 | 0.9441 |
| **R CN/SOC** | **L Putamen** | **fNT** | 0.1842 | 0.1215 | 0.1342 | 0.3105 |
| **R CN/SOC** | **L Putamen** | **MD** | 0.2862 | 0.119 | 0.0189 | 0.0293 |
| **R CN/SOC** | **L Putamen** | **PV** | 0.0585 | 0.1207 | 0.6296 | 0.8783 |
| **R CN/SOC** | **L Putamen** | **RD** | 0.2119 | 0.121 | 0.0844 | 0.1151 |
| **R CN/SOC** | **L SFC** | **AD** | 0.2487 | 0.1206 | 0.0431 | 0.0764 |
| **R CN/SOC** | **L SFC** | **FA** | -0.0806 | -0.1233 | 0.5158 | 0.845 |
| **R CN/SOC** | **L SFC** | **fNT** | 0.1914 | 0.1250 | 0.1177 | 0.2858 |
| **R CN/SOC** | **L SFC** | **MD** | 0.2143 | 0.1238 | 0.0882 | 0.0994 |
| **R CN/SOC** | **L SFC** | **PV** | 0.0945 | 0.1166 | 0.4205 | 0.8148 |
| **R CN/SOC** | **L SFC** | **RD** | 0.2848 | 0.1185 | 0.019 | 0.039 |
| **R CN/SOC** | **R Amygdala** | **AD** | 0.2335 | 0.1213 | 0.0586 | 0.0923 |
| **R CN/SOC** | **R Amygdala** | **FA** | 0.007 | 0.1211 | 0.9542 | 0.9804 |
| **R CN/SOC** | **R Amygdala** | **fNT** | -0.0095 | -0.1134 | 0.9385 | 0.9637 |
| **R CN/SOC** | **R Amygdala** | **MD** | 0.2267 | 0.121 | 0.0655 | 0.0769 |
| **R CN/SOC** | **R Amygdala** | **PV** | 0.128 | 0.124 | 0.3057 | 0.7039 |
| **R CN/SOC** | **R Amygdala** | **RD** | 0.171 | 0.121 | 0.1625 | 0.1968 |
| **R CN/SOC** | **R Anterior** | **AD** | 0.3777 | 0.1155 | 0.0017 | 0.0156 |
| **R CN/SOC** | **R Anterior** | **FA** | -0.0316 | -0.1249 | 0.8012 | 0.9432 |
| **R CN/SOC** | **R Anterior** | **fNT** | -0.4753 | -0.1063 | <10^-4^ | 0.0032 |
| **R CN/SOC** | **R Anterior** | **MD** | 0.3422 | 0.1185 | 0.0053 | 0.0133 |
| **R CN/SOC** | **R Anterior** | **PV** | -0.3978 | -0.1134 | 0.0008 | 0.0514 |
| **R CN/SOC** | **R Anterior** | **RD** | 0.2548 | 0.122 | 0.0407 | 0.066 |
| **R CN/SOC** | **R Caudate** | **AD** | 0.1893 | 0.1232 | 0.1293 | 0.1703 |
| **R CN/SOC** | **R Caudate** | **FA** | -0.1898 | -0.1232 | 0.1285 | 0.4494 |
| **R CN/SOC** | **R Caudate** | **fNT** | -0.0528 | -0.1214 | 0.6651 | 0.8222 |
| **R CN/SOC** | **R Caudate** | **MD** | 0.356 | 0.1177 | 0.0036 | 0.0119 |
| **R CN/SOC** | **R Caudate** | **PV** | -0.2653 | -0.12 | 0.0306 | 0.2077 |
| **R CN/SOC** | **R Caudate** | **RD** | 0.3932 | 0.1159 | 0.0012 | 0.0118 |
| **R CN/SOC** | **R Medio_Dorsal** | **AD** | 0.2967 | 0.1202 | 0.0163 | 0.0436 |
| **R CN/SOC** | **R Medio_Dorsal** | **FA** | 0.0964 | 0.1251 | 0.444 | 0.8159 |
| **R CN/SOC** | **R Medio_Dorsal** | **fNT** | -0.5445 | -0.1213 | <10^-4^ | 0.0018 |
| **R CN/SOC** | **R Medio_Dorsal** | **MD** | 0.2199 | 0.1259 | 0.0858 | 0.0969 |
| **R CN/SOC** | **R Medio_Dorsal** | **PV** | -0.1635 | -0.121 | 0.1813 | 0.5612 |
| **R CN/SOC** | **R Medio_Dorsal** | **RD** | 0.1703 | 0.1248 | 0.1774 | 0.2118 |
| **R CN/SOC** | **R Pallidum** | **AD** | 0.3737 | 0.1153 | 0.0018 | 0.0156 |
| **R CN/SOC** | **R Pallidum** | **FA** | -0.07 | -0.1241 | 0.5746 | 0.8672 |
| **R CN/SOC** | **R Pallidum** | **fNT** | 0.282 | 0.1121 | 0.0143 | 0.071 |
| **R CN/SOC** | **R Pallidum** | **MD** | 0.4025 | 0.1138 | 0.0007 | 0.0077 |
| **R CN/SOC** | **R Pallidum** | **PV** | 0.1957 | 0.1107 | 0.0818 | 0.3607 |
| **R CN/SOC** | **R Pallidum** | **RD** | 0.3724 | 0.1154 | 0.0019 | 0.0148 |
| **R CN/SOC** | **R Putamen** | **AD** | 0.3992 | 0.1138 | 0.0008 | 0.0137 |
| **R CN/SOC** | **R Putamen** | **FA** | 0.0066 | 0.1238 | 0.9575 | 0.9804 |
| **R CN/SOC** | **R Putamen** | **fNT** | 0.3316 | 0.1103 | 0.0037 | 0.0303 |
| **R CN/SOC** | **R Putamen** | **MD** | 0.4118 | 0.1137 | 0.0006 | 0.0077 |
| **R CN/SOC** | **R Putamen** | **PV** | 0.2029 | 0.1231 | 0.1042 | 0.4053 |
| **R CN/SOC** | **R Putamen** | **RD** | 0.3545 | 0.1168 | 0.0034 | 0.0188 |
| **R CN/SOC** | **R rMFC** | **AD** | 0.293 | 0.1202 | 0.0175 | 0.0444 |
| **R CN/SOC** | **R rMFC** | **FA** | -0.3053 | -0.1201 | 0.0135 | 0.1639 |
| **R CN/SOC** | **R rMFC** | **fNT** | -0.1868 | -0.1132 | 0.1251 | 0.2926 |
| **R CN/SOC** | **R rMFC** | **MD** | 0.3544 | 0.1181 | 0.0038 | 0.0121 |
| **R CN/SOC** | **R rMFC** | **PV** | -0.0621 | -0.1251 | 0.6211 | 0.8688 |
| **R CN/SOC** | **R rMFC** | **RD** | 0.3514 | 0.118 | 0.0041 | 0.0204 |
| **R CN/SOC** | **R SFC** | **AD** | 0.2721 | 0.1185 | 0.0248 | 0.0547 |
| **R CN/SOC** | **R SFC** | **FA** | -0.2657 | -0.1181 | 0.0278 | 0.2161 |
| **R CN/SOC** | **R SFC** | **fNT** | -0.0591 | -0.1243 | 0.6358 | 0.8097 |
| **R CN/SOC** | **R SFC** | **MD** | 0.4219 | 0.1133 | 0.0004 | 0.0077 |
| **R CN/SOC** | **R SFC** | **PV** | -0.07 | -0.1198 | 0.5612 | 0.8552 |
| **R CN/SOC** | **R SFC** | **RD** | 0.4345 | 0.1122 | 0.0002 | 0.006 |
| **R CN/SOC** | **R SPC** | **AD** | 0.2893 | 0.118 | 0.0169 | 0.0444 |
| **R CN/SOC** | **R SPC** | **FA** | 0.0473 | 0.1256 | 0.7075 | 0.8967 |
| **R CN/SOC** | **R SPC** | **fNT** | 0.2259 | 0.1234 | 0.0721 | 0.2071 |
| **R CN/SOC** | **R SPC** | **MD** | 0.2923 | 0.1205 | 0.018 | 0.0283 |
| **R CN/SOC** | **R SPC** | **PV** | 0.0417 | 0.1245 | 0.7384 | 0.9381 |
| **R CN/SOC** | **R SPC** | **RD** | 0.2105 | 0.1234 | 0.0926 | 0.1246 |
| **R IC** | **R Anterior** | **AD** | 0.3239 | 0.1179 | 0.0077 | 0.0297 |
| **R IC** | **R Anterior** | **FA** | -0.2872 | -0.118 | 0.0176 | 0.1809 |
| **R IC** | **R Anterior** | **fNT** | -0.4198 | -0.1213 | 0.001 | 0.0135 |
| **R IC** | **R Anterior** | **MD** | 0.4239 | 0.1122 | 0.0003 | 0.0077 |
| **R IC** | **R Anterior** | **PV** | -0.3201 | -0.1099 | 0.0049 | 0.1056 |
| **R IC** | **R Anterior** | **RD** | 0.4264 | 0.1114 | 0.0003 | 0.0061 |
| **R IC** | **R Caudate** | **AD** | 0.3649 | 0.1161 | 0.0025 | 0.0179 |
| **R IC** | **R Caudate** | **FA** | -0.0477 | -0.1249 | 0.704 | 0.8967 |
| **R IC** | **R Caudate** | **fNT** | -0.1257 | -0.1211 | 0.3031 | 0.5151 |
| **R IC** | **R Caudate** | **MD** | 0.4063 | 0.114 | 0.0007 | 0.0077 |
| **R IC** | **R Caudate** | **PV** | -0.245 | -0.1159 | 0.0383 | 0.2164 |
| **R IC** | **R Caudate** | **RD** | 0.327 | 0.1181 | 0.0073 | 0.0245 |
| **R IC** | **R Medio_Dorsal** | **AD** | 0.2955 | 0.1195 | 0.016 | 0.0436 |
| **R IC** | **R Medio_Dorsal** | **FA** | 0.1725 | 0.123 | 0.1656 | 0.5011 |
| **R IC** | **R Medio_Dorsal** | **fNT** | -0.3634 | -0.1065 | 0.0011 | 0.0139 |
| **R IC** | **R Medio_Dorsal** | **MD** | 0.2441 | 0.122 | 0.0496 | 0.0604 |
| **R IC** | **R Medio_Dorsal** | **PV** | -0.157 | -0.116 | 0.1807 | 0.5612 |
| **R IC** | **R Medio_Dorsal** | **RD** | 0.1684 | 0.1244 | 0.1807 | 0.2142 |
| **R IC** | **R Pallidum** | **AD** | 0.3444 | 0.117 | 0.0044 | 0.0215 |
| **R IC** | **R Pallidum** | **FA** | 0.2113 | 0.1209 | 0.0851 | 0.3679 |
| **R IC** | **R Pallidum** | **fNT** | -0.0562 | -0.1211 | 0.6441 | 0.8162 |
| **R IC** | **R Pallidum** | **MD** | 0.2563 | 0.1187 | 0.0344 | 0.0461 |
| **R IC** | **R Pallidum** | **PV** | 0.0058 | 0.1222 | 0.9625 | 0.9751 |
| **R IC** | **R Pallidum** | **RD** | 0.0428 | 0.1223 | 0.7273 | 0.7514 |
| **R IC** | **R Putamen** | **AD** | 0.2565 | 0.1202 | 0.0365 | 0.0708 |
| **R IC** | **R Putamen** | **FA** | -0.0565 | -0.1242 | 0.6505 | 0.8932 |
| **R IC** | **R Putamen** | **fNT** | 0.2273 | 0.127 | 0.0785 | 0.2149 |
| **R IC** | **R Putamen** | **MD** | 0.2676 | 0.1198 | 0.029 | 0.04 |
| **R IC** | **R Putamen** | **PV** | 0.0203 | 0.1217 | 0.868 | 0.9544 |
| **R IC** | **R Putamen** | **RD** | 0.2559 | 0.1206 | 0.0375 | 0.0623 |
| **R IC** | **R SFC** | **AD** | 0.3445 | 0.1176 | 0.0047 | 0.0221 |
| **R IC** | **R SFC** | **FA** | -0.1126 | -0.1244 | 0.3687 | 0.7459 |
| **R IC** | **R SFC** | **fNT** | -0.1698 | -0.1221 | 0.1688 | 0.3533 |
| **R IC** | **R SFC** | **MD** | 0.3611 | 0.117 | 0.003 | 0.0108 |
| **R IC** | **R SFC** | **PV** | -0.1919 | -0.1196 | 0.1133 | 0.415 |
| **R IC** | **R SFC** | **RD** | 0.3032 | 0.1196 | 0.0136 | 0.0326 |
| **R IC** | **R SPC** | **AD** | 0.2652 | 0.1244 | 0.0371 | 0.0711 |
| **R IC** | **R SPC** | **FA** | -0.0786 | -0.1243 | 0.5294 | 0.8588 |
| **R IC** | **R SPC** | **fNT** | -0.0251 | -0.1227 | 0.8385 | 0.9342 |
| **R IC** | **R SPC** | **MD** | 0.3506 | 0.1192 | 0.0046 | 0.0126 |
| **R IC** | **R SPC** | **PV** | -0.0636 | -0.1257 | 0.6144 | 0.8688 |
| **R IC** | **R SPC** | **RD** | 0.347 | 0.1185 | 0.0047 | 0.0207 |
| **R MGN** | **R Amygdala** | **AD** | 0.254 | 0.1205 | 0.0388 | 0.0735 |
| **R MGN** | **R Amygdala** | **FA** | -0.1202 | -0.123 | 0.3317 | 0.7066 |
| **R MGN** | **R Amygdala** | **fNT** | -0.2638 | -0.1192 | 0.0304 | 0.1193 |
| **R MGN** | **R Amygdala** | **MD** | 0.3314 | 0.1174 | 0.0063 | 0.0148 |
| **R MGN** | **R Amygdala** | **PV** | -0.268 | -0.1207 | 0.0299 | 0.2077 |
| **R MGN** | **R Amygdala** | **RD** | 0.2811 | 0.1192 | 0.0213 | 0.0417 |
| **R MGN** | **R Anterior** | **AD** | 0.1985 | 0.1232 | 0.112 | 0.1508 |
| **R MGN** | **R Anterior** | **FA** | -0.1987 | -0.1223 | 0.109 | 0.4108 |
| **R MGN** | **R Anterior** | **fNT** | -0.4605 | -0.1076 | 0.0001 | 0.0039 |
| **R MGN** | **R Anterior** | **MD** | 0.4028 | 0.1155 | 0.0009 | 0.0077 |
| **R MGN** | **R Anterior** | **PV** | -0.342 | -0.1157 | 0.0043 | 0.1056 |
| **R MGN** | **R Anterior** | **RD** | 0.417 | 0.1141 | 0.0005 | 0.008 |
| **R MGN** | **R Caudate** | **AD** | 0.2187 | 0.1192 | 0.0711 | 0.1067 |
| **R MGN** | **R Caudate** | **FA** | -0.2172 | -0.1213 | 0.0779 | 0.3529 |
| **R MGN** | **R Caudate** | **fNT** | -0.3349 | -0.1143 | 0.0046 | 0.0349 |
| **R MGN** | **R Caudate** | **MD** | 0.354 | 0.1145 | 0.0029 | 0.0107 |
| **R MGN** | **R Caudate** | **PV** | -0.2374 | -0.1153 | 0.0433 | 0.2247 |
| **R MGN** | **R Caudate** | **RD** | 0.3707 | 0.1148 | 0.0019 | 0.0148 |
| **R MGN** | **R entorhinal** | **AD** | 0.3539 | 0.1151 | 0.0031 | 0.0196 |
| **R MGN** | **R entorhinal** | **FA** | -0.0158 | -0.1239 | 0.8986 | 0.9747 |
| **R MGN** | **R entorhinal** | **fNT** | -0.1564 | -0.1194 | 0.1947 | 0.3894 |
| **R MGN** | **R entorhinal** | **MD** | 0.4047 | 0.1132 | 0.0007 | 0.0077 |
| **R MGN** | **R entorhinal** | **PV** | 0.0203 | 0.1226 | 0.8688 | 0.9544 |
| **R MGN** | **R entorhinal** | **RD** | 0.3558 | 0.1164 | 0.0032 | 0.0188 |
| **R MGN** | **R fusiform** | **AD** | 0.3026 | 0.1176 | 0.0123 | 0.0367 |
| **R MGN** | **R fusiform** | **FA** | 0.0211 | 0.1245 | 0.8657 | 0.963 |
| **R MGN** | **R fusiform** | **fNT** | 0.0212 | 0.1256 | 0.8647 | 0.9372 |
| **R MGN** | **R fusiform** | **MD** | 0.2631 | 0.1193 | 0.0308 | 0.042 |
| **R MGN** | **R fusiform** | **PV** | 0.1282 | 0.1229 | 0.3005 | 0.7027 |
| **R MGN** | **R fusiform** | **RD** | 0.1866 | 0.1219 | 0.1306 | 0.1638 |
| **R MGN** | **R Hippo_CA1** | **AD** | 0.2682 | 0.1189 | 0.0275 | 0.0589 |
| **R MGN** | **R Hippo_CA1** | **FA** | -0.1419 | -0.1224 | 0.2504 | 0.6215 |
| **R MGN** | **R Hippo_CA1** | **fNT** | 0.2339 | 0.1309 | 0.0794 | 0.2161 |
| **R MGN** | **R Hippo_CA1** | **MD** | 0.2622 | 0.121 | 0.0341 | 0.0458 |
| **R MGN** | **R Hippo_CA1** | **PV** | -0.0299 | -0.1236 | 0.8098 | 0.9544 |
| **R MGN** | **R Hippo_CA1** | **RD** | 0.2318 | 0.1218 | 0.0615 | 0.0891 |
| **R MGN** | **R IPC** | **AD** | 0.3739 | 0.1185 | 0.0024 | 0.0179 |
| **R MGN** | **R IPC** | **FA** | -0.0482 | -0.1262 | 0.7039 | 0.8967 |
| **R MGN** | **R IPC** | **fNT** | -0.0697 | -0.1268 | 0.5844 | 0.7782 |
| **R MGN** | **R IPC** | **MD** | 0.3578 | 0.1185 | 0.0036 | 0.0119 |
| **R MGN** | **R IPC** | **PV** | 0.0878 | 0.1245 | 0.4831 | 0.8412 |
| **R MGN** | **R IPC** | **RD** | 0.2711 | 0.1215 | 0.0291 | 0.0528 |
| **R MGN** | **R ITC** | **AD** | 0.3487 | 0.1177 | 0.0042 | 0.0212 |
| **R MGN** | **R ITC** | **FA** | 0.1857 | 0.1226 | 0.1345 | 0.4524 |
| **R MGN** | **R ITC** | **fNT** | 0.2136 | 0.1213 | 0.0829 | 0.2195 |
| **R MGN** | **R ITC** | **MD** | 0.1069 | 0.1238 | 0.391 | 0.404 |
| **R MGN** | **R ITC** | **PV** | 0.3881 | 0.1152 | 0.0013 | 0.0675 |
| **R MGN** | **R ITC** | **RD** | -0.0301 | -0.1243 | 0.8094 | 0.8226 |
| **R MGN** | **R Medio_Dorsal** | **AD** | 0.1691 | 0.1247 | 0.1798 | 0.2261 |
| **R MGN** | **R Medio_Dorsal** | **FA** | 0.0463 | 0.1262 | 0.7152 | 0.8979 |
| **R MGN** | **R Medio_Dorsal** | **fNT** | -0.3545 | -0.1153 | 0.0031 | 0.0271 |
| **R MGN** | **R Medio_Dorsal** | **MD** | 0.2301 | 0.1229 | 0.0658 | 0.0771 |
| **R MGN** | **R Medio_Dorsal** | **PV** | -0.4159 | -0.1234 | 0.0014 | 0.0675 |
| **R MGN** | **R Medio_Dorsal** | **RD** | 0.1656 | 0.1245 | 0.1881 | 0.2209 |
| **R MGN** | **R Pallidum** | **AD** | 0.3374 | 0.1168 | 0.0052 | 0.0236 |
| **R MGN** | **R Pallidum** | **FA** | 0.175 | 0.1219 | 0.1558 | 0.5009 |
| **R MGN** | **R Pallidum** | **fNT** | -0.2488 | -0.1183 | 0.0392 | 0.1385 |
| **R MGN** | **R Pallidum** | **MD** | 0.2487 | 0.1199 | 0.0418 | 0.0526 |
| **R MGN** | **R Pallidum** | **PV** | 0.0122 | 0.1189 | 0.9187 | 0.9544 |
| **R MGN** | **R Pallidum** | **RD** | 0.0908 | 0.1225 | 0.4614 | 0.491 |
| **R MGN** | **R precuneus** | **AD** | 0.1062 | 0.123 | 0.3911 | 0.4347 |
| **R MGN** | **R precuneus** | **FA** | -0.1922 | -0.1201 | 0.1142 | 0.4151 |
| **R MGN** | **R precuneus** | **fNT** | -0.1368 | -0.1235 | 0.2719 | 0.4751 |
| **R MGN** | **R precuneus** | **MD** | 0.2283 | 0.1209 | 0.0632 | 0.0745 |
| **R MGN** | **R precuneus** | **PV** | -0.1171 | -0.1239 | 0.3482 | 0.7387 |
| **R MGN** | **R precuneus** | **RD** | 0.2657 | 0.1193 | 0.0293 | 0.0528 |
| **R MGN** | **R Putamen** | **AD** | 0.2753 | 0.1197 | 0.0246 | 0.0547 |
| **R MGN** | **R Putamen** | **FA** | -0.0356 | -0.1221 | 0.7713 | 0.9254 |
| **R MGN** | **R Putamen** | **fNT** | 0.2707 | 0.1268 | 0.037 | 0.1336 |
| **R MGN** | **R Putamen** | **MD** | 0.2622 | 0.1184 | 0.0303 | 0.0415 |
| **R MGN** | **R Putamen** | **PV** | 0.0658 | 0.1228 | 0.5938 | 0.8652 |
| **R MGN** | **R Putamen** | **RD** | 0.2647 | 0.1181 | 0.0283 | 0.0518 |
| **R MGN** | **R rMFC** | **AD** | 0.3026 | 0.1204 | 0.0146 | 0.0413 |
| **R MGN** | **R rMFC** | **FA** | -0.1045 | -0.126 | 0.4097 | 0.779 |
| **R MGN** | **R rMFC** | **fNT** | -0.4244 | -0.1201 | 0.0004 | 0.0083 |
| **R MGN** | **R rMFC** | **MD** | 0.3537 | 0.1185 | 0.004 | 0.0121 |
| **R MGN** | **R rMFC** | **PV** | -0.1416 | -0.1234 | 0.2554 | 0.659 |
| **R MGN** | **R rMFC** | **RD** | 0.3298 | 0.1199 | 0.0077 | 0.0252 |
| **R MGN** | **R SFC** | **AD** | 0.2328 | 0.1201 | 0.0569 | 0.0903 |
| **R MGN** | **R SFC** | **FA** | -0.1884 | -0.1205 | 0.1226 | 0.4363 |
| **R MGN** | **R SFC** | **fNT** | -0.1657 | -0.1206 | 0.1741 | 0.3582 |
| **R MGN** | **R SFC** | **MD** | 0.3303 | 0.1164 | 0.006 | 0.0145 |
| **R MGN** | **R SFC** | **PV** | -0.2007 | -0.1163 | 0.0892 | 0.3696 |
| **R MGN** | **R SFC** | **RD** | 0.3229 | 0.1164 | 0.0072 | 0.0244 |
| **R MGN** | **R SPC** | **AD** | 0.2933 | 0.1188 | 0.0161 | 0.0436 |
| **R MGN** | **R SPC** | **FA** | -0.0628 | -0.1257 | 0.6187 | 0.8912 |
| **R MGN** | **R SPC** | **fNT** | -0.1666 | -0.1225 | 0.1785 | 0.364 |
| **R MGN** | **R SPC** | **MD** | 0.3384 | 0.1181 | 0.0056 | 0.0138 |
| **R MGN** | **R SPC** | **PV** | -0.1248 | -0.117 | 0.29 | 0.6909 |
| **R MGN** | **R SPC** | **RD** | 0.2897 | 0.1203 | 0.0188 | 0.0388 |
| **R MGN** | **R Ventral_Anterior** | **AD** | 0.219 | 0.1216 | 0.0762 | 0.1119 |
| **R MGN** | **R Ventral_Anterior** | **FA** | -0.0817 | -0.1232 | 0.5094 | 0.8401 |
| **R MGN** | **R Ventral_Anterior** | **fNT** | -0.1811 | -0.1219 | 0.1422 | 0.3176 |
| **R MGN** | **R Ventral_Anterior** | **MD** | 0.303 | 0.1184 | 0.0128 | 0.0224 |
| **R MGN** | **R Ventral_Anterior** | **PV** | -0.254 | -0.1153 | 0.0312 | 0.2077 |
| **R MGN** | **R Ventral_Anterior** | **RD** | 0.3007 | 0.1177 | 0.0129 | 0.0325 |
| **R PAC** | **R Amygdala** | **AD** | 0.2425 | 0.1164 | 0.0409 | 0.0749 |
| **R PAC** | **R Amygdala** | **FA** | -0.0951 | -0.123 | 0.4421 | 0.8159 |
| **R PAC** | **R Amygdala** | **fNT** | 0.0541 | 0.1212 | 0.6567 | 0.8222 |
| **R PAC** | **R Amygdala** | **MD** | 0.3548 | 0.1139 | 0.0027 | 0.0103 |
| **R PAC** | **R Amygdala** | **PV** | 0.1225 | 0.1221 | 0.3197 | 0.7136 |
| **R PAC** | **R Amygdala** | **RD** | 0.3165 | 0.1173 | 0.0088 | 0.0272 |
| **R PAC** | **R Anterior** | **AD** | 0.3328 | 0.1206 | 0.0076 | 0.0297 |
| **R PAC** | **R Anterior** | **FA** | -0.1666 | -0.1236 | 0.1824 | 0.528 |
| **R PAC** | **R Anterior** | **fNT** | -0.3068 | -0.1157 | 0.0102 | 0.0565 |
| **R PAC** | **R Anterior** | **MD** | 0.3554 | 0.1151 | 0.003 | 0.0108 |
| **R PAC** | **R Anterior** | **PV** | -0.2784 | -0.125 | 0.0298 | 0.2077 |
| **R PAC** | **R Anterior** | **RD** | 0.3393 | 0.1162 | 0.0048 | 0.0207 |
| **R PAC** | **R Caudate** | **AD** | 0.3135 | 0.1184 | 0.0102 | 0.0327 |
| **R PAC** | **R Caudate** | **FA** | -0.3026 | -0.1188 | 0.0132 | 0.1639 |
| **R PAC** | **R Caudate** | **fNT** | 0.129 | 0.122 | 0.2942 | 0.5034 |
| **R PAC** | **R Caudate** | **MD** | 0.4235 | 0.1121 | 0.0003 | 0.0077 |
| **R PAC** | **R Caudate** | **PV** | -0.0892 | -0.1235 | 0.4728 | 0.8412 |
| **R PAC** | **R Caudate** | **RD** | 0.4512 | 0.1116 | 0.0001 | 0.0058 |
| **R PAC** | **R fusiform** | **AD** | 0.2262 | 0.1158 | 0.055 | 0.0884 |
| **R PAC** | **R fusiform** | **FA** | -0.2312 | -0.1221 | 0.0628 | 0.3124 |
| **R PAC** | **R fusiform** | **fNT** | 0.0881 | 0.1246 | 0.4818 | 0.7037 |
| **R PAC** | **R fusiform** | **MD** | 0.4108 | 0.1112 | 0.0005 | 0.0077 |
| **R PAC** | **R fusiform** | **PV** | 0.2453 | 0.1189 | 0.0431 | 0.2247 |
| **R PAC** | **R fusiform** | **RD** | 0.415 | 0.1132 | 0.0005 | 0.008 |
| **R PAC** | **R IPC** | **AD** | 0.3899 | 0.1122 | 0.0009 | 0.0137 |
| **R PAC** | **R IPC** | **FA** | -0.0498 | -0.1235 | 0.6881 | 0.8932 |
| **R PAC** | **R IPC** | **fNT** | -0.0722 | -0.124 | 0.5621 | 0.7652 |
| **R PAC** | **R IPC** | **MD** | 0.3832 | 0.1137 | 0.0012 | 0.0079 |
| **R PAC** | **R IPC** | **PV** | -0.0778 | -0.1194 | 0.5167 | 0.8461 |
| **R PAC** | **R IPC** | **RD** | 0.3383 | 0.1166 | 0.005 | 0.0207 |
| **R PAC** | **R ITC** | **AD** | 0.3676 | 0.104 | 0.0007 | 0.0137 |
| **R PAC** | **R ITC** | **FA** | -0.0676 | -0.1228 | 0.5839 | 0.8727 |
| **R PAC** | **R ITC** | **fNT** | 0.1123 | 0.123 | 0.3644 | 0.577 |
| **R PAC** | **R ITC** | **MD** | 0.3964 | 0.1095 | 0.0006 | 0.0077 |
| **R PAC** | **R ITC** | **PV** | 0.2098 | 0.1193 | 0.0831 | 0.3607 |
| **R PAC** | **R ITC** | **RD** | 0.3384 | 0.1163 | 0.0049 | 0.0207 |
| **R PAC** | **R LOC** | **AD** | 0.3244 | 0.108 | 0.0037 | 0.0208 |
| **R PAC** | **R LOC** | **FA** | -0.0875 | -0.1227 | 0.4783 | 0.8235 |
| **R PAC** | **R LOC** | **fNT** | 0.0267 | 0.1219 | 0.8269 | 0.9234 |
| **R PAC** | **R LOC** | **MD** | 0.3369 | 0.113 | 0.004 | 0.0121 |
| **R PAC** | **R LOC** | **PV** | -0.0068 | -0.123 | 0.9557 | 0.9751 |
| **R PAC** | **R LOC** | **RD** | 0.2747 | 0.119 | 0.024 | 0.0455 |
| **R PAC** | **R lOFC** | **AD** | 0.3647 | 0.1156 | 0.0024 | 0.0179 |
| **R PAC** | **R lOFC** | **FA** | -0.0981 | -0.1233 | 0.4292 | 0.8047 |
| **R PAC** | **R lOFC** | **fNT** | 0.0511 | 0.1243 | 0.6823 | 0.8328 |
| **R PAC** | **R lOFC** | **MD** | 0.4059 | 0.1107 | 0.0005 | 0.0077 |
| **R PAC** | **R lOFC** | **PV** | 0.0432 | 0.1229 | 0.7265 | 0.9379 |
| **R PAC** | **R lOFC** | **RD** | 0.3305 | 0.1147 | 0.0053 | 0.0215 |
| **R PAC** | **R Pallidum** | **AD** | 0.3435 | 0.1148 | 0.0039 | 0.0208 |
| **R PAC** | **R Pallidum** | **FA** | -0.0043 | -0.1241 | 0.9722 | 0.9901 |
| **R PAC** | **R Pallidum** | **fNT** | 0.0157 | 0.1224 | 0.8983 | 0.942 |
| **R PAC** | **R Pallidum** | **MD** | 0.2456 | 0.1143 | 0.0353 | 0.047 |
| **R PAC** | **R Pallidum** | **PV** | 0.0636 | 0.1221 | 0.6046 | 0.8652 |
| **R PAC** | **R Pallidum** | **RD** | 0.1704 | 0.1188 | 0.1562 | 0.1902 |
| **R PAC** | **R pars** | **AD** | 0.4165 | 0.1122 | 0.0004 | 0.0137 |
| **R PAC** | **R pars** | **FA** | -0.0994 | -0.1217 | 0.4173 | 0.7882 |
| **R PAC** | **R pars** | **fNT** | -0.0462 | -0.119 | 0.6993 | 0.8432 |
| **R PAC** | **R pars** | **MD** | 0.3874 | 0.1118 | 0.0009 | 0.0077 |
| **R PAC** | **R pars** | **PV** | 0.0434 | 0.1237 | 0.727 | 0.9379 |
| **R PAC** | **R pars** | **RD** | 0.3106 | 0.1151 | 0.0088 | 0.0272 |
| **R PAC** | **R parstr** | **AD** | 0.4903 | 0.1088 | <10^-4^ | 0.0046 |
| **R PAC** | **R parstr** | **FA** | 0.0929 | 0.1229 | 0.4527 | 0.8227 |
| **R PAC** | **R parstr** | **fNT** | 0.2216 | 0.1202 | 0.0698 | 0.2044 |
| **R PAC** | **R parstr** | **MD** | 0.4307 | 0.1112 | 0.0002 | 0.0077 |
| **R PAC** | **R parstr** | **PV** | 0.1011 | 0.1244 | 0.4196 | 0.8148 |
| **R PAC** | **R parstr** | **RD** | 0.2962 | 0.1169 | 0.0137 | 0.0326 |
| **R PAC** | **R Putamen** | **AD** | 0.369 | 0.109 | 0.0012 | 0.0144 |
| **R PAC** | **R Putamen** | **FA** | -0.0064 | -0.1239 | 0.9588 | 0.9804 |
| **R PAC** | **R Putamen** | **fNT** | 0.0403 | 0.1242 | 0.7468 | 0.8771 |
| **R PAC** | **R Putamen** | **MD** | 0.3524 | 0.1122 | 0.0025 | 0.0101 |
| **R PAC** | **R Putamen** | **PV** | -0.0237 | -0.1229 | 0.848 | 0.9544 |
| **R PAC** | **R Putamen** | **RD** | 0.2696 | 0.118 | 0.0254 | 0.0473 |
| **R PAC** | **R SPC** | **AD** | 0.3147 | 0.1146 | 0.0077 | 0.0297 |
| **R PAC** | **R SPC** | **FA** | -0.1734 | -0.1219 | 0.1595 | 0.5011 |
| **R PAC** | **R SPC** | **fNT** | 0.0662 | 0.1238 | 0.5948 | 0.7899 |
| **R PAC** | **R SPC** | **MD** | 0.3733 | 0.1123 | 0.0014 | 0.0082 |
| **R PAC** | **R SPC** | **PV** | 0.1501 | 0.1208 | 0.2184 | 0.6187 |
| **R PAC** | **R SPC** | **RD** | 0.3466 | 0.1146 | 0.0035 | 0.0191 |
| **R PAC** | **R TP** | **AD** | 0.2725 | 0.1148 | 0.0204 | 0.0492 |
| **R PAC** | **R TP** | **FA** | -0.0875 | -0.1227 | 0.4781 | 0.8235 |
| **R PAC** | **R TP** | **fNT** | -0.4403 | -0.1207 | 0.0006 | 0.0102 |
| **R PAC** | **R TP** | **MD** | 0.3362 | 0.1158 | 0.005 | 0.0132 |
| **R PAC** | **R TP** | **PV** | 0.1036 | 0.1236 | 0.4052 | 0.7993 |
| **R PAC** | **R TP** | **RD** | 0.3031 | 0.1186 | 0.0129 | 0.0325 |

### Appendix C: Structural network: CHUU vs CPHIV

Table 6: Standardized Beta (Std β), Standard Error (Std SE) values, p values and FDR corrected q values for the comparison between CHUU and CPHIV over the nodal graph measures degree, strength, transitivity, nodal efficiency and local efficiency.

|  |  |  | degree | |  |  |  | strength | |  |  |  | transitivity | |  |  |  | nodal efficiency | | |  |  | local efficiency | | |
| --- | --- | --- | --- | --- | --- | --- | --- | --- | --- | --- | --- | --- | --- | --- | --- | --- | --- | --- | --- | --- | --- | --- | --- | --- | --- |
| ROI |  | **Std β** | **Std SE** | **p-value** | **q-value** | | **Std β** | **Std SE** | **p-value** | **q-value** | | **Std β** | **Std SE** | **p-value** | **q-value** | | **Std β** | **Std SE** | **p-value** | **q-value** | | **Std β** | **Std SE** | **p-value** | **q-value** |
| L-SOC-CN |  | 0.13 | 0.12 | 0.28 | 0.72 |  | 0.11 | 0.11 | 0.34 | 0.56 |  | -0.02 | 0.12 | 0.86 | 0.94 |  | -0.07 | 0.12 | 0.54 | 0.98 |  | -0.07 | 0.11 | 0.53 | 0.88 |
| L-MGN |  | 0.03 | 0.12 | 0.81 | 0.90 |  | -0.11 | 0.12 | 0.37 | 0.57 |  | -0.17 | 0.12 | 0.15 | 0.66 |  | 0.04 | 0.11 | 0.69 | 0.98 |  | -0.07 | 0.12 | 0.55 | 0.89 |
| L-IC |  | -0.19 | 0.12 | 0.11 | 0.51 |  | -0.11 | 0.12 | 0.36 | 0.57 |  | -0.08 | 0.12 | 0.50 | 0.84 |  | -0.24 | 0.12 | 0.05 | 0.49 |  | -0.25 | 0.12 | 0.04 | 0.42 |
| L-PAC |  | 0.10 | 0.12 | 0.40 | 0.74 |  | -0.12 | 0.12 | 0.29 | 0.54 |  | -0.23 | 0.12 | 0.05 | 0.43 |  | 0.00 | 0.11 | 0.99 | 1.00 |  | -0.01 | 0.12 | 0.96 | 0.99 |
| R-SOC-CN |  | 0.11 | 0.12 | 0.37 | 0.74 |  | 0.15 | 0.11 | 0.17 | 0.37 |  | 0.10 | 0.12 | 0.44 | 0.84 |  | -0.26 | 0.11 | 0.02 | 0.49 |  | 0.05 | 0.11 | 0.70 | 0.92 |
| R-MGN |  | -0.02 | 0.12 | 0.90 | 0.95 |  | -0.08 | 0.12 | 0.52 | 0.75 |  | 0.07 | 0.12 | 0.58 | 0.84 |  | -0.04 | 0.12 | 0.74 | 0.98 |  | -0.04 | 0.12 | 0.73 | 0.92 |
| R-IC |  | -0.14 | 0.12 | 0.27 | 0.72 |  | -0.16 | 0.11 | 0.16 | 0.37 |  | 0.03 | 0.12 | 0.78 | 0.94 |  | -0.20 | 0.12 | 0.11 | 0.73 |  | -0.09 | 0.12 | 0.47 | 0.82 |
| R-PAC |  | 0.17 | 0.12 | 0.18 | 0.63 |  | 0.17 | 0.12 | 0.15 | 0.37 |  | -0.12 | 0.12 | 0.32 | 0.76 |  | -0.02 | 0.12 | 0.84 | 1.00 |  | 0.01 | 0.12 | 0.96 | 0.99 |
| R-lOFC |  | -0.11 | 0.12 | 0.38 | 0.74 |  | -0.27 | 0.12 | 0.03 | 0.13 |  | 0.00 | 0.12 | 0.97 | 0.98 |  | 0.14 | 0.12 | 0.23 | 0.98 |  | -0.05 | 0.12 | 0.66 | 0.91 |
| R-pars |  | 0.10 | 0.12 | 0.41 | 0.74 |  | 0.05 | 0.12 | 0.69 | 0.83 |  | -0.12 | 0.12 | 0.32 | 0.76 |  | -0.11 | 0.12 | 0.37 | 0.98 |  | 0.11 | 0.12 | 0.37 | 0.82 |
| R-FP |  | -0.11 | 0.12 | 0.37 | 0.74 |  | -0.11 | 0.12 | 0.37 | 0.57 |  | -0.18 | 0.11 | 0.13 | 0.60 |  | -0.25 | 0.12 | 0.04 | 0.49 |  | -0.22 | 0.12 | 0.06 | 0.42 |
| R-mOFC |  | 0.09 | 0.12 | 0.45 | 0.78 |  | -0.07 | 0.12 | 0.57 | 0.78 |  | -0.22 | 0.12 | 0.06 | 0.45 |  | 0.19 | 0.11 | 0.08 | 0.73 |  | -0.15 | 0.12 | 0.22 | 0.65 |
| R-parstr |  | 0.09 | 0.12 | 0.46 | 0.79 |  | 0.00 | 0.12 | 0.97 | 0.99 |  | -0.04 | 0.12 | 0.77 | 0.94 |  | -0.02 | 0.12 | 0.88 | 1.00 |  | 0.17 | 0.12 | 0.17 | 0.62 |
| R-rMFC |  | -0.22 | 0.12 | 0.07 | 0.39 |  | -0.30 | 0.12 | 0.02 | 0.11 |  | -0.02 | 0.12 | 0.85 | 0.94 |  | 0.12 | 0.12 | 0.33 | 0.98 |  | 0.03 | 0.11 | 0.81 | 0.97 |
| R-SFC |  | -0.17 | 0.12 | 0.18 | 0.63 |  | -0.27 | 0.13 | 0.03 | 0.17 |  | -0.11 | 0.12 | 0.36 | 0.77 |  | -0.12 | 0.12 | 0.31 | 0.98 |  | -0.28 | 0.11 | 0.02 | 0.33 |
| R-cMFC |  | 0.16 | 0.12 | 0.19 | 0.63 |  | -0.20 | 0.12 | 0.10 | 0.29 |  | -0.34 | 0.12 | 0.01 | 0.28 |  | 0.19 | 0.11 | 0.10 | 0.73 |  | 0.17 | 0.12 | 0.15 | 0.62 |
| R-paracentral |  | -0.26 | 0.12 | 0.03 | 0.24 |  | -0.03 | 0.12 | 0.80 | 0.89 |  | -0.04 | 0.12 | 0.73 | 0.94 |  | -0.03 | 0.12 | 0.77 | 0.98 |  | -0.23 | 0.12 | 0.06 | 0.42 |
| R-rACC |  | -0.10 | 0.12 | 0.41 | 0.74 |  | -0.16 | 0.12 | 0.18 | 0.38 |  | -0.04 | 0.12 | 0.75 | 0.94 |  | -0.07 | 0.12 | 0.57 | 0.98 |  | -0.04 | 0.12 | 0.72 | 0.92 |
| R-cACC |  | -0.31 | 0.12 | 0.01 | 0.12 |  | -0.16 | 0.11 | 0.17 | 0.37 |  | 0.12 | 0.12 | 0.33 | 0.76 |  | -0.18 | 0.12 | 0.13 | 0.73 |  | -0.20 | 0.11 | 0.08 | 0.43 |
| R-PCC |  | -0.38 | 0.11 | 0.00 | 0.03 |  | 0.00 | 0.12 | 1.00 | 1.00 |  | 0.21 | 0.12 | 0.08 | 0.48 |  | -0.27 | 0.12 | 0.03 | 0.49 |  | -0.16 | 0.12 | 0.16 | 0.62 |
| R-SPC |  | 0.10 | 0.12 | 0.41 | 0.74 |  | -0.06 | 0.12 | 0.60 | 0.78 |  | -0.16 | 0.12 | 0.19 | 0.71 |  | 0.00 | 0.12 | 0.97 | 1.00 |  | -0.05 | 0.12 | 0.66 | 0.91 |
| R-IPC |  | -0.04 | 0.12 | 0.78 | 0.88 |  | -0.07 | 0.12 | 0.55 | 0.76 |  | 0.01 | 0.12 | 0.95 | 0.97 |  | -0.02 | 0.12 | 0.89 | 1.00 |  | -0.09 | 0.12 | 0.46 | 0.82 |
| R-precuneus |  | -0.06 | 0.12 | 0.65 | 0.86 |  | -0.31 | 0.12 | 0.02 | 0.11 |  | -0.14 | 0.12 | 0.25 | 0.76 |  | -0.01 | 0.12 | 0.96 | 1.00 |  | -0.29 | 0.11 | 0.01 | 0.26 |
| R-cuneus |  | 0.26 | 0.12 | 0.03 | 0.24 |  | -0.05 | 0.12 | 0.68 | 0.83 |  | 0.19 | 0.12 | 0.12 | 0.60 |  | 0.06 | 0.12 | 0.61 | 0.98 |  | 0.10 | 0.12 | 0.41 | 0.82 |
| R-pericalc |  | 0.10 | 0.12 | 0.41 | 0.74 |  | -0.30 | 0.12 | 0.02 | 0.11 |  | 0.07 | 0.12 | 0.56 | 0.84 |  | 0.00 | 0.12 | 0.98 | 1.00 |  | -0.03 | 0.12 | 0.80 | 0.97 |
| R-LOC |  | -0.04 | 0.12 | 0.75 | 0.88 |  | -0.20 | 0.11 | 0.08 | 0.24 |  | 0.11 | 0.12 | 0.35 | 0.77 |  | -0.09 | 0.11 | 0.42 | 0.98 |  | -0.05 | 0.12 | 0.67 | 0.91 |
| R-fusiform |  | 0.08 | 0.12 | 0.53 | 0.81 |  | -0.13 | 0.12 | 0.26 | 0.51 |  | -0.03 | 0.12 | 0.79 | 0.94 |  | -0.08 | 0.12 | 0.49 | 0.98 |  | -0.01 | 0.12 | 0.94 | 0.99 |
| R-entorhinal |  | 0.00 | 0.12 | 0.99 | 0.99 |  | -0.02 | 0.12 | 0.86 | 0.91 |  | 0.11 | 0.12 | 0.39 | 0.79 |  | 0.04 | 0.12 | 0.73 | 0.98 |  | 0.18 | 0.12 | 0.14 | 0.62 |
| R-TP |  | 0.06 | 0.12 | 0.66 | 0.86 |  | -0.23 | 0.12 | 0.06 | 0.21 |  | -0.16 | 0.12 | 0.18 | 0.71 |  | 0.05 | 0.12 | 0.67 | 0.98 |  | 0.09 | 0.12 | 0.46 | 0.82 |
| R-ITC |  | 0.17 | 0.12 | 0.17 | 0.63 |  | -0.07 | 0.12 | 0.54 | 0.76 |  | -0.07 | 0.12 | 0.56 | 0.84 |  | -0.02 | 0.12 | 0.89 | 1.00 |  | 0.10 | 0.12 | 0.42 | 0.82 |
| R-Anterior |  | -0.46 | 0.10 | 0.00 | 0.00 |  | -0.43 | 0.11 | 0.00 | 0.00 |  | 0.13 | 0.12 | 0.30 | 0.76 |  | -0.05 | 0.12 | 0.68 | 0.98 |  | -0.24 | 0.11 | 0.03 | 0.42 |
| R-Medio_Dorsal |  | -0.35 | 0.12 | 0.00 | 0.05 |  | -0.40 | 0.11 | 0.00 | 0.01 |  | 0.07 | 0.12 | 0.56 | 0.84 |  | -0.03 | 0.11 | 0.76 | 0.98 |  | -0.37 | 0.11 | 0.00 | 0.10 |
| R-V_Anterior |  | -0.30 | 0.12 | 0.01 | 0.12 |  | -0.44 | 0.11 | 0.00 | 0.00 |  | 0.02 | 0.12 | 0.87 | 0.94 |  | -0.25 | 0.12 | 0.04 | 0.49 |  | -0.15 | 0.11 | 0.19 | 0.62 |
| R-Caudate |  | -0.39 | 0.11 | 0.00 | 0.03 |  | -0.21 | 0.12 | 0.08 | 0.24 |  | 0.06 | 0.12 | 0.63 | 0.84 |  | -0.14 | 0.11 | 0.24 | 0.98 |  | -0.14 | 0.11 | 0.20 | 0.63 |
| R-Pallidum |  | -0.08 | 0.12 | 0.54 | 0.82 |  | 0.02 | 0.12 | 0.89 | 0.93 |  | 0.13 | 0.12 | 0.30 | 0.76 |  | -0.06 | 0.12 | 0.61 | 0.98 |  | 0.00 | 0.11 | 0.98 | 0.99 |
| R-Accumbens |  | -0.02 | 0.12 | 0.90 | 0.95 |  | -0.27 | 0.11 | 0.02 | 0.11 |  | -0.27 | 0.12 | 0.02 | 0.35 |  | 0.01 | 0.12 | 0.90 | 1.00 |  | -0.10 | 0.12 | 0.41 | 0.82 |
| R-Amygdala |  | 0.13 | 0.12 | 0.31 | 0.72 |  | -0.21 | 0.12 | 0.08 | 0.24 |  | -0.08 | 0.12 | 0.52 | 0.84 |  | -0.04 | 0.11 | 0.71 | 0.98 |  | 0.00 | 0.12 | 0.98 | 0.99 |
| R-Hippo_HATA |  | 0.07 | 0.12 | 0.58 | 0.85 |  | 0.22 | 0.11 | 0.06 | 0.21 |  | -0.06 | 0.12 | 0.61 | 0.84 |  | -0.04 | 0.12 | 0.72 | 0.98 |  | 0.07 | 0.12 | 0.57 | 0.89 |
| L-lOFC |  | -0.14 | 0.12 | 0.26 | 0.72 |  | -0.48 | 0.11 | 0.00 | 0.00 |  | -0.25 | 0.12 | 0.04 | 0.43 |  | 0.03 | 0.11 | 0.78 | 0.98 |  | -0.10 | 0.12 | 0.38 | 0.82 |
| L-pars |  | -0.13 | 0.12 | 0.30 | 0.72 |  | -0.27 | 0.11 | 0.02 | 0.11 |  | 0.00 | 0.12 | 0.98 | 0.98 |  | 0.06 | 0.12 | 0.62 | 0.98 |  | 0.00 | 0.12 | 0.99 | 0.99 |
| L-FP |  | -0.16 | 0.12 | 0.19 | 0.63 |  | -0.29 | 0.12 | 0.02 | 0.11 |  | -0.07 | 0.12 | 0.57 | 0.84 |  | 0.00 | 0.12 | 0.99 | 1.00 |  | -0.10 | 0.12 | 0.40 | 0.82 |
| L-mOFC |  | -0.14 | 0.12 | 0.25 | 0.72 |  | -0.14 | 0.12 | 0.25 | 0.48 |  | -0.29 | 0.12 | 0.02 | 0.35 |  | -0.23 | 0.11 | 0.04 | 0.49 |  | -0.20 | 0.11 | 0.08 | 0.43 |
| L-parstr |  | -0.08 | 0.12 | 0.52 | 0.81 |  | -0.25 | 0.12 | 0.04 | 0.17 |  | 0.01 | 0.12 | 0.92 | 0.96 |  | 0.03 | 0.12 | 0.79 | 0.98 |  | 0.10 | 0.12 | 0.43 | 0.82 |
| L-rMFC |  | 0.04 | 0.12 | 0.73 | 0.88 |  | -0.28 | 0.11 | 0.02 | 0.11 |  | -0.23 | 0.12 | 0.05 | 0.43 |  | 0.08 | 0.12 | 0.50 | 0.98 |  | 0.16 | 0.12 | 0.19 | 0.62 |
| L-SFC |  | 0.05 | 0.12 | 0.66 | 0.86 |  | -0.12 | 0.12 | 0.32 | 0.55 |  | -0.16 | 0.12 | 0.20 | 0.71 |  | -0.03 | 0.12 | 0.76 | 0.98 |  | -0.07 | 0.11 | 0.50 | 0.84 |
| L-cMFC |  | -0.09 | 0.12 | 0.48 | 0.80 |  | 0.05 | 0.12 | 0.70 | 0.83 |  | 0.08 | 0.12 | 0.50 | 0.84 |  | 0.03 | 0.12 | 0.81 | 0.99 |  | -0.06 | 0.12 | 0.62 | 0.91 |
| L-paracentral |  | 0.04 | 0.12 | 0.77 | 0.88 |  | 0.20 | 0.12 | 0.11 | 0.30 |  | -0.16 | 0.12 | 0.21 | 0.71 |  | -0.09 | 0.12 | 0.46 | 0.98 |  | -0.09 | 0.12 | 0.45 | 0.82 |
| L-rACC |  | -0.16 | 0.12 | 0.20 | 0.63 |  | -0.13 | 0.12 | 0.29 | 0.54 |  | -0.21 | 0.12 | 0.09 | 0.48 |  | -0.10 | 0.12 | 0.43 | 0.98 |  | -0.11 | 0.12 | 0.36 | 0.82 |
| L-cACC |  | -0.25 | 0.12 | 0.04 | 0.24 |  | -0.07 | 0.12 | 0.59 | 0.78 |  | 0.04 | 0.12 | 0.73 | 0.94 |  | 0.04 | 0.12 | 0.72 | 0.98 |  | -0.21 | 0.12 | 0.08 | 0.43 |
| L-PCC |  | -0.34 | 0.11 | 0.00 | 0.05 |  | -0.05 | 0.12 | 0.67 | 0.83 |  | 0.21 | 0.12 | 0.08 | 0.48 |  | -0.14 | 0.12 | 0.24 | 0.98 |  | -0.22 | 0.12 | 0.07 | 0.43 |
| L-SPC |  | 0.03 | 0.12 | 0.78 | 0.88 |  | -0.12 | 0.12 | 0.32 | 0.55 |  | -0.06 | 0.12 | 0.61 | 0.84 |  | -0.04 | 0.12 | 0.73 | 0.98 |  | 0.02 | 0.11 | 0.87 | 0.99 |
| L-IPC |  | -0.03 | 0.12 | 0.83 | 0.90 |  | 0.01 | 0.12 | 0.96 | 0.99 |  | 0.16 | 0.12 | 0.20 | 0.71 |  | 0.17 | 0.11 | 0.13 | 0.73 |  | -0.04 | 0.12 | 0.76 | 0.94 |
| L-precuneus |  | 0.06 | 0.12 | 0.63 | 0.86 |  | 0.15 | 0.12 | 0.23 | 0.48 |  | -0.28 | 0.12 | 0.02 | 0.35 |  | -0.10 | 0.12 | 0.40 | 0.98 |  | -0.36 | 0.12 | 0.00 | 0.16 |
| L-cuneus |  | 0.00 | 0.12 | 0.98 | 0.99 |  | 0.10 | 0.12 | 0.39 | 0.59 |  | -0.12 | 0.12 | 0.32 | 0.76 |  | -0.01 | 0.12 | 0.93 | 1.00 |  | -0.07 | 0.12 | 0.58 | 0.89 |
| L-pericalc |  | -0.06 | 0.12 | 0.64 | 0.86 |  | -0.05 | 0.12 | 0.71 | 0.83 |  | 0.09 | 0.12 | 0.46 | 0.84 |  | 0.06 | 0.12 | 0.61 | 0.98 |  | 0.01 | 0.12 | 0.92 | 0.99 |
| L-LOC |  | 0.01 | 0.12 | 0.97 | 0.99 |  | -0.25 | 0.12 | 0.04 | 0.17 |  | 0.02 | 0.12 | 0.87 | 0.94 |  | -0.13 | 0.12 | 0.25 | 0.98 |  | 0.06 | 0.11 | 0.59 | 0.90 |
| L-fusiform |  | -0.14 | 0.11 | 0.22 | 0.68 |  | -0.17 | 0.12 | 0.16 | 0.37 |  | 0.12 | 0.12 | 0.33 | 0.76 |  | -0.09 | 0.11 | 0.41 | 0.98 |  | -0.10 | 0.11 | 0.36 | 0.82 |
| L-entorhinal |  | 0.01 | 0.12 | 0.94 | 0.98 |  | 0.06 | 0.12 | 0.60 | 0.78 |  | 0.09 | 0.12 | 0.48 | 0.84 |  | -0.04 | 0.12 | 0.74 | 0.98 |  | -0.09 | 0.12 | 0.47 | 0.82 |
| L-TP |  | -0.04 | 0.12 | 0.73 | 0.88 |  | -0.17 | 0.12 | 0.16 | 0.37 |  | -0.08 | 0.12 | 0.50 | 0.84 |  | -0.10 | 0.12 | 0.38 | 0.98 |  | -0.12 | 0.11 | 0.27 | 0.73 |
| L-ITC |  | -0.05 | 0.12 | 0.67 | 0.87 |  | -0.03 | 0.12 | 0.80 | 0.89 |  | -0.03 | 0.12 | 0.81 | 0.94 |  | -0.11 | 0.12 | 0.35 | 0.98 |  | -0.06 | 0.12 | 0.61 | 0.90 |
| L-Anterior |  | -0.21 | 0.12 | 0.09 | 0.45 |  | -0.47 | 0.11 | 0.00 | 0.00 |  | -0.14 | 0.12 | 0.25 | 0.76 |  | 0.17 | 0.12 | 0.15 | 0.78 |  | -0.15 | 0.11 | 0.18 | 0.62 |
| L-VLD |  | -0.16 | 0.12 | 0.18 | 0.63 |  | -0.02 | 0.12 | 0.84 | 0.91 |  | 0.06 | 0.12 | 0.61 | 0.84 |  | 0.12 | 0.12 | 0.32 | 0.98 |  | -0.13 | 0.11 | 0.23 | 0.66 |
| L-V_Anterior |  | -0.22 | 0.12 | 0.08 | 0.41 |  | -0.30 | 0.12 | 0.01 | 0.11 |  | 0.08 | 0.12 | 0.51 | 0.84 |  | 0.00 | 0.12 | 1.00 | 1.00 |  | -0.16 | 0.11 | 0.16 | 0.62 |
| L-Caudate |  | -0.26 | 0.12 | 0.03 | 0.23 |  | -0.21 | 0.11 | 0.07 | 0.24 |  | 0.11 | 0.12 | 0.38 | 0.79 |  | -0.04 | 0.12 | 0.73 | 0.98 |  | -0.21 | 0.11 | 0.06 | 0.42 |
| L-Putamen |  | 0.07 | 0.12 | 0.59 | 0.86 |  | -0.08 | 0.12 | 0.47 | 0.69 |  | -0.10 | 0.12 | 0.42 | 0.82 |  | -0.12 | 0.12 | 0.32 | 0.98 |  | -0.04 | 0.11 | 0.71 | 0.92 |
| L-Accumbens |  | 0.05 | 0.12 | 0.69 | 0.88 |  | 0.14 | 0.12 | 0.23 | 0.48 |  | 0.41 | 0.11 | 0.00 | 0.04 |  | -0.05 | 0.10 | 0.63 | 0.98 |  | 0.00 | 0.12 | 0.99 | 0.99 |
| L-Amygdala |  | 0.08 | 0.12 | 0.50 | 0.81 |  | -0.04 | 0.12 | 0.73 | 0.83 |  | -0.03 | 0.12 | 0.80 | 0.94 |  | -0.24 | 0.12 | 0.04 | 0.49 |  | 0.00 | 0.11 | 0.97 | 0.99 |
| L-Hippo_CA1 |  | 0.13 | 0.12 | 0.29 | 0.72 |  | 0.12 | 0.12 | 0.32 | 0.55 |  | 0.02 | 0.12 | 0.88 | 0.94 |  | -0.12 | 0.11 | 0.30 | 0.98 |  | 0.22 | 0.11 | 0.06 | 0.42 |
| L-Hippo_CA3 |  | 0.11 | 0.12 | 0.38 | 0.74 |  | 0.02 | 0.12 | 0.84 | 0.91 |  | 0.02 | 0.12 | 0.87 | 0.94 |  | -0.19 | 0.12 | 0.12 | 0.73 |  | 0.03 | 0.12 | 0.84 | 0.97 |
| L-Hippo_CA4 |  | 0.10 | 0.12 | 0.42 | 0.74 |  | 0.04 | 0.12 | 0.72 | 0.83 |  | -0.27 | 0.12 | 0.03 | 0.39 |  | 0.03 | 0.12 | 0.78 | 0.98 |  | 0.12 | 0.12 | 0.32 | 0.82 |
| L-Hippo_HATA |  | 0.04 | 0.12 | 0.77 | 0.88 |  | 0.23 | 0.12 | 0.05 | 0.21 |  | 0.12 | 0.12 | 0.33 | 0.76 |  | -0.02 | 0.11 | 0.87 | 1.00 |  | 0.03 | 0.12 | 0.82 | 0.97 |

### Appendix D: DTI based tractography: CPHIV Means and Standard Deviations.

Table 7: Means and Standard deviations (SD) of 10 random groups of 22 CPHIV. Each row represents a WM connection and the DTI parameters being summarized.

|  |  |  | **Sample 1** | | **Sample 2** | | **Sample 3** | | **Sample 4** | | **Sample 5** | | **Sample 6** | | **Sample 7** | | **Sample 8** | | **Sample 9** | | **Sample 10** | |
| --- | --- | --- | --- | --- | --- | --- | --- | --- | --- | --- | --- | --- | --- | --- | --- | --- | --- | --- | --- | --- | --- | --- |
| **from** | **to** | **par** | **mean** | **SD** | **mean** | **SD** | **mean** | **SD** | **mean** | **SD** | **mean** | **SD** | **mean** | **SD** | **mean** | **SD** | **mean** | **SD** | **mean** | **SD** | **mean** | **SD** |
| **L-VLD** | **L-SOC-CN** | **MD** | 0,7150 | 0,0254 | 0,7133 | 0,0243 | 0,7090 | 0,0316 | 0,7026 | 0,0246 | 0,7170 | 0,0227 | 0,7154 | 0,0278 | 0,7092 | 0,0324 | 0,7043 | 0,0243 | 0,7157 | 0,0282 | 0,7144 | 0,0228 |
| **L-VLD** | **L-SOC-CN** | **RD** | 0,5392 | 0,0260 | 0,5410 | 0,0215 | 0,5335 | 0,0307 | 0,5320 | 0,0230 | 0,5383 | 0,0250 | 0,5393 | 0,0301 | 0,5351 | 0,0321 | 0,5316 | 0,0187 | 0,5380 | 0,0315 | 0,5409 | 0,0276 |
| **L-VLD** | **L-SOC-CN** | **AD** | 1,0667 | 0,0749 | 1,0579 | 0,0731 | 1,0600 | 0,0751 | 1,0437 | 0,0612 | 1,0746 | 0,0686 | 1,0677 | 0,0734 | 1,0574 | 0,0870 | 1,0496 | 0,0862 | 1,0711 | 0,0693 | 1,0615 | 0,0423 |
| **L-rMFC** | **L-SOC-CN** | **MD** | 0,7266 | 0,0248 | 0,7286 | 0,0241 | 0,7249 | 0,0296 | 0,7250 | 0,0202 | 0,7299 | 0,0250 | 0,7267 | 0,0267 | 0,7243 | 0,0206 | 0,7272 | 0,0186 | 0,7247 | 0,0278 | 0,7272 | 0,0251 |
| **L-rMFC** | **L-SOC-CN** | **RD** | 0,5510 | 0,0276 | 0,5526 | 0,0262 | 0,5497 | 0,0330 | 0,5486 | 0,0215 | 0,5528 | 0,0308 | 0,5478 | 0,0316 | 0,5495 | 0,0239 | 0,5539 | 0,0209 | 0,5486 | 0,0323 | 0,5500 | 0,0285 |
| **L-rMFC** | **L-SOC-CN** | **AD** | 1,0824 | 0,0217 | 1,0806 | 0,0295 | 1,0797 | 0,0295 | 1,0820 | 0,0211 | 1,0841 | 0,0265 | 1,0844 | 0,0244 | 1,0738 | 0,0268 | 1,0739 | 0,0249 | 1,0811 | 0,0272 | 1,0817 | 0,0234 |
| **R-Putamen** | **L-SOC-CN** | **MD** | 0,6835 | 0,0199 | 0,6756 | 0,0237 | 0,6752 | 0,0240 | 0,6821 | 0,0220 | 0,6769 | 0,0215 | 0,6781 | 0,0193 | 0,6783 | 0,0225 | 0,6777 | 0,0217 | 0,6828 | 0,0200 | 0,6810 | 0,0225 |
| **R-Putamen** | **L-SOC-CN** | **RD** | 0,4876 | 0,0156 | 0,4815 | 0,0221 | 0,4832 | 0,0204 | 0,4869 | 0,0223 | 0,4829 | 0,0190 | 0,4846 | 0,0175 | 0,4845 | 0,0215 | 0,4828 | 0,0194 | 0,4884 | 0,0187 | 0,4851 | 0,0211 |
| **R-Putamen** | **L-SOC-CN** | **AD** | 1,0753 | 0,0401 | 1,0638 | 0,0335 | 1,0592 | 0,0379 | 1,0725 | 0,0360 | 1,0649 | 0,0380 | 1,0651 | 0,0299 | 1,0658 | 0,0362 | 1,0677 | 0,0386 | 1,0718 | 0,0335 | 1,0726 | 0,0372 |
| **L-Anterior** | **L-SOC-CN** | **MD** | 0,7092 | 0,0234 | 0,7022 | 0,0242 | 0,7028 | 0,0237 | 0,7123 | 0,0223 | 0,7080 | 0,0202 | 0,7081 | 0,0269 | 0,7145 | 0,0246 | 0,7024 | 0,0240 | 0,7113 | 0,0243 | 0,7146 | 0,0279 |
| **L-Anterior** | **L-SOC-CN** | **RD** | 0,5342 | 0,0223 | 0,5238 | 0,0251 | 0,5231 | 0,0268 | 0,5347 | 0,0291 | 0,5273 | 0,0234 | 0,5295 | 0,0307 | 0,5371 | 0,0271 | 0,5303 | 0,0264 | 0,5311 | 0,0268 | 0,5364 | 0,0291 |
| **L-Anterior** | **L-SOC-CN** | **AD** | 1,0590 | 0,0443 | 1,0591 | 0,0514 | 1,0621 | 0,0570 | 1,0676 | 0,0469 | 1,0696 | 0,0506 | 1,0653 | 0,0468 | 1,0695 | 0,0419 | 1,0467 | 0,0361 | 1,0718 | 0,0462 | 1,0710 | 0,0487 |
| **L-SFC** | **L-SOC-CN** | **MD** | 0,7133 | 0,0164 | 0,7132 | 0,0221 | 0,7132 | 0,0222 | 0,7124 | 0,0166 | 0,7153 | 0,0220 | 0,7168 | 0,0208 | 0,7158 | 0,0184 | 0,7124 | 0,0165 | 0,7163 | 0,0207 | 0,7169 | 0,0210 |
| **L-SFC** | **L-SOC-CN** | **RD** | 0,5220 | 0,0175 | 0,5225 | 0,0212 | 0,5242 | 0,0212 | 0,5227 | 0,0169 | 0,5262 | 0,0225 | 0,5273 | 0,0209 | 0,5292 | 0,0188 | 0,5230 | 0,0188 | 0,5260 | 0,0230 | 0,5267 | 0,0218 |
| **L-SFC** | **L-SOC-CN** | **AD** | 1,0961 | 0,0236 | 1,0945 | 0,0316 | 1,0913 | 0,0325 | 1,0916 | 0,0278 | 1,0936 | 0,0294 | 1,0958 | 0,0269 | 1,0891 | 0,0241 | 1,0913 | 0,0221 | 1,0969 | 0,0231 | 1,0972 | 0,0266 |
| **L-Putamen** | **L-SOC-CN** | **MD** | 0,6942 | 0,0148 | 0,6917 | 0,0169 | 0,6890 | 0,0188 | 0,6929 | 0,0174 | 0,6912 | 0,0191 | 0,6929 | 0,0141 | 0,6929 | 0,0191 | 0,6910 | 0,0177 | 0,6957 | 0,0181 | 0,6987 | 0,0175 |
| **L-Putamen** | **L-SOC-CN** | **RD** | 0,4988 | 0,0144 | 0,4966 | 0,0167 | 0,4956 | 0,0190 | 0,4988 | 0,0150 | 0,4965 | 0,0172 | 0,4977 | 0,0155 | 0,4994 | 0,0172 | 0,4957 | 0,0177 | 0,5005 | 0,0197 | 0,5019 | 0,0177 |
| **L-Putamen** | **L-SOC-CN** | **AD** | 1,0850 | 0,0253 | 1,0820 | 0,0257 | 1,0759 | 0,0276 | 1,0811 | 0,0303 | 1,0806 | 0,0305 | 1,0833 | 0,0211 | 1,0797 | 0,0306 | 1,0817 | 0,0268 | 1,0862 | 0,0232 | 1,0923 | 0,0240 |
| **L-Pallidum** | **L-SOC-CN** | **MD** | 0,6922 | 0,0162 | 0,6870 | 0,0156 | 0,6856 | 0,0174 | 0,6894 | 0,0175 | 0,6900 | 0,0187 | 0,6885 | 0,0108 | 0,6894 | 0,0180 | 0,6887 | 0,0171 | 0,6921 | 0,0181 | 0,6939 | 0,0185 |
| **L-Pallidum** | **L-SOC-CN** | **RD** | 0,5051 | 0,0131 | 0,5000 | 0,0141 | 0,5001 | 0,0148 | 0,5024 | 0,0134 | 0,5019 | 0,0161 | 0,5015 | 0,0115 | 0,5023 | 0,0154 | 0,5009 | 0,0143 | 0,5036 | 0,0171 | 0,5042 | 0,0175 |
| **L-Pallidum** | **L-SOC-CN** | **AD** | 1,0664 | 0,0291 | 1,0609 | 0,0239 | 1,0566 | 0,0269 | 1,0632 | 0,0320 | 1,0662 | 0,0321 | 1,0624 | 0,0166 | 1,0636 | 0,0311 | 1,0645 | 0,0282 | 1,0692 | 0,0251 | 1,0734 | 0,0275 |
| **L-SPC** | **L-SOC-CN** | **MD** | 0,7313 | 0,0200 | 0,7294 | 0,0254 | 0,7286 | 0,0294 | 0,7276 | 0,0205 | 0,7347 | 0,0261 | 0,7308 | 0,0225 | 0,7270 | 0,0229 | 0,7299 | 0,0199 | 0,7291 | 0,0303 | 0,7332 | 0,0290 |
| **L-SPC** | **L-SOC-CN** | **RD** | 0,5294 | 0,0267 | 0,5308 | 0,0297 | 0,5320 | 0,0338 | 0,5282 | 0,0264 | 0,5341 | 0,0313 | 0,5310 | 0,0276 | 0,5269 | 0,0284 | 0,5313 | 0,0238 | 0,5273 | 0,0329 | 0,5298 | 0,0346 |
| **L-SPC** | **L-SOC-CN** | **AD** | 1,1353 | 0,0346 | 1,1264 | 0,0320 | 1,1220 | 0,0361 | 1,1264 | 0,0349 | 1,1358 | 0,0300 | 1,1303 | 0,0261 | 1,1274 | 0,0274 | 1,1271 | 0,0287 | 1,1329 | 0,0375 | 1,1401 | 0,0330 |
| **R-Pallidum** | **L-SOC-CN** | **MD** | 0,6851 | 0,0212 | 0,6759 | 0,0241 | 0,6754 | 0,0249 | 0,6812 | 0,0217 | 0,6774 | 0,0225 | 0,6783 | 0,0201 | 0,6796 | 0,0215 | 0,6775 | 0,0220 | 0,6849 | 0,0202 | 0,6828 | 0,0237 |
| **R-Pallidum** | **L-SOC-CN** | **RD** | 0,4942 | 0,0161 | 0,4867 | 0,0223 | 0,4879 | 0,0211 | 0,4904 | 0,0204 | 0,4881 | 0,0188 | 0,4894 | 0,0185 | 0,4902 | 0,0198 | 0,4865 | 0,0181 | 0,4945 | 0,0182 | 0,4911 | 0,0224 |
| **R-Pallidum** | **L-SOC-CN** | **AD** | 1,0668 | 0,0426 | 1,0543 | 0,0344 | 1,0504 | 0,0399 | 1,0629 | 0,0368 | 1,0560 | 0,0389 | 1,0562 | 0,0309 | 1,0584 | 0,0344 | 1,0595 | 0,0398 | 1,0656 | 0,0333 | 1,0662 | 0,0375 |
| **L-Hippo_CA1** | **L-SOC-CN** | **MD** | 0,6687 | 0,0292 | 0,6562 | 0,0282 | 0,6664 | 0,0341 | 0,6594 | 0,0263 | 0,6661 | 0,0259 | 0,6692 | 0,0333 | 0,6601 | 0,0320 | 0,6595 | 0,0370 | 0,6699 | 0,0319 | 0,6698 | 0,0218 |
| **L-Hippo_CA1** | **L-SOC-CN** | **RD** | 0,4699 | 0,0324 | 0,4510 | 0,0237 | 0,4625 | 0,0351 | 0,4558 | 0,0243 | 0,4659 | 0,0304 | 0,4650 | 0,0348 | 0,4583 | 0,0336 | 0,4558 | 0,0296 | 0,4646 | 0,0337 | 0,4607 | 0,0221 |
| **L-Hippo_CA1** | **L-SOC-CN** | **AD** | 1,0663 | 0,0671 | 1,0666 | 0,0468 | 1,0741 | 0,0707 | 1,0668 | 0,0570 | 1,0665 | 0,0615 | 1,0776 | 0,0665 | 1,0636 | 0,0673 | 1,0669 | 0,0685 | 1,0805 | 0,0691 | 1,0880 | 0,0475 |
| **L-Caudate** | **L-SOC-CN** | **MD** | 0,6961 | 0,0228 | 0,6965 | 0,0203 | 0,6926 | 0,0211 | 0,6975 | 0,0171 | 0,6948 | 0,0211 | 0,6960 | 0,0185 | 0,6955 | 0,0201 | 0,6938 | 0,0166 | 0,6959 | 0,0221 | 0,7005 | 0,0215 |
| **L-Caudate** | **L-SOC-CN** | **RD** | 0,5211 | 0,0237 | 0,5206 | 0,0222 | 0,5164 | 0,0226 | 0,5200 | 0,0185 | 0,5209 | 0,0228 | 0,5192 | 0,0208 | 0,5237 | 0,0221 | 0,5203 | 0,0196 | 0,5237 | 0,0244 | 0,5254 | 0,0255 |
| **L-Caudate** | **L-SOC-CN** | **AD** | 1,0462 | 0,0280 | 1,0483 | 0,0296 | 1,0451 | 0,0334 | 1,0527 | 0,0259 | 1,0477 | 0,0239 | 1,0496 | 0,0266 | 1,0435 | 0,0267 | 1,0407 | 0,0260 | 1,0403 | 0,0321 | 1,0506 | 0,0256 |
| **R-SFC** | **L-SOC-CN** | **MD** | 0,7196 | 0,0254 | 0,7153 | 0,0259 | 0,7153 | 0,0261 | 0,7145 | 0,0244 | 0,7162 | 0,0261 | 0,7176 | 0,0225 | 0,7140 | 0,0263 | 0,7156 | 0,0254 | 0,7183 | 0,0247 | 0,7134 | 0,0241 |
| **R-SFC** | **L-SOC-CN** | **RD** | 0,5183 | 0,0221 | 0,5165 | 0,0237 | 0,5182 | 0,0237 | 0,5157 | 0,0235 | 0,5185 | 0,0239 | 0,5193 | 0,0231 | 0,5182 | 0,0251 | 0,5170 | 0,0216 | 0,5194 | 0,0239 | 0,5144 | 0,0232 |
| **R-SFC** | **L-SOC-CN** | **AD** | 1,1221 | 0,0377 | 1,1129 | 0,0369 | 1,1094 | 0,0399 | 1,1121 | 0,0357 | 1,1116 | 0,0384 | 1,1141 | 0,0290 | 1,1055 | 0,0373 | 1,1129 | 0,0412 | 1,1163 | 0,0342 | 1,1114 | 0,0340 |
| **L-Pallidum** | **L-MGN** | **MD** | 0,6996 | 0,0184 | 0,7067 | 0,0270 | 0,7039 | 0,0286 | 0,7006 | 0,0254 | 0,6999 | 0,0302 | 0,7050 | 0,0254 | 0,7003 | 0,0311 | 0,7041 | 0,0247 | 0,7091 | 0,0230 | 0,7039 | 0,0204 |
| **L-Pallidum** | **L-MGN** | **RD** | 0,5024 | 0,0176 | 0,5114 | 0,0242 | 0,5091 | 0,0266 | 0,5059 | 0,0241 | 0,5043 | 0,0247 | 0,5083 | 0,0224 | 0,5040 | 0,0252 | 0,5060 | 0,0236 | 0,5104 | 0,0245 | 0,5053 | 0,0208 |
| **L-Pallidum** | **L-MGN** | **AD** | 1,0940 | 0,0347 | 1,0972 | 0,0381 | 1,0933 | 0,0396 | 1,0901 | 0,0394 | 1,0977 | 0,0428 | 1,0985 | 0,0380 | 1,0996 | 0,0454 | 1,1003 | 0,0394 | 1,1066 | 0,0326 | 1,1010 | 0,0358 |
| **L-rMFC** | **L-MGN** | **MD** | 0,7389 | 0,0251 | 0,7486 | 0,0277 | 0,7439 | 0,0325 | 0,7409 | 0,0191 | 0,7432 | 0,0310 | 0,7406 | 0,0313 | 0,7394 | 0,0269 | 0,7469 | 0,0198 | 0,7429 | 0,0319 | 0,7429 | 0,0245 |
| **L-rMFC** | **L-MGN** | **RD** | 0,5637 | 0,0299 | 0,5714 | 0,0286 | 0,5658 | 0,0395 | 0,5655 | 0,0237 | 0,5695 | 0,0338 | 0,5599 | 0,0341 | 0,5622 | 0,0311 | 0,5704 | 0,0248 | 0,5651 | 0,0378 | 0,5646 | 0,0313 |
| **L-rMFC** | **L-MGN** | **AD** | 1,0893 | 0,0365 | 1,1029 | 0,0417 | 1,1000 | 0,0481 | 1,0918 | 0,0379 | 1,0906 | 0,0451 | 1,1020 | 0,0469 | 1,0940 | 0,0479 | 1,1000 | 0,0403 | 1,0985 | 0,0488 | 1,0995 | 0,0419 |
| **L-SFC** | **L-MGN** | **MD** | 0,7213 | 0,0187 | 0,7260 | 0,0242 | 0,7260 | 0,0262 | 0,7240 | 0,0198 | 0,7270 | 0,0263 | 0,7270 | 0,0246 | 0,7286 | 0,0226 | 0,7265 | 0,0187 | 0,7272 | 0,0246 | 0,7282 | 0,0243 |
| **L-SFC** | **L-MGN** | **RD** | 0,5295 | 0,0225 | 0,5364 | 0,0217 | 0,5344 | 0,0247 | 0,5350 | 0,0219 | 0,5373 | 0,0281 | 0,5376 | 0,0219 | 0,5383 | 0,0243 | 0,5382 | 0,0238 | 0,5328 | 0,0284 | 0,5381 | 0,0270 |
| **L-SFC** | **L-MGN** | **AD** | 1,1049 | 0,0261 | 1,1050 | 0,0389 | 1,1091 | 0,0375 | 1,1021 | 0,0368 | 1,1063 | 0,0357 | 1,1058 | 0,0376 | 1,1093 | 0,0339 | 1,1031 | 0,0257 | 1,1160 | 0,0295 | 1,1085 | 0,0325 |
| **L-Caudate** | **L-MGN** | **MD** | 0,7023 | 0,0204 | 0,7073 | 0,0212 | 0,7073 | 0,0188 | 0,7083 | 0,0200 | 0,7027 | 0,0262 | 0,7018 | 0,0185 | 0,7032 | 0,0266 | 0,7084 | 0,0218 | 0,7082 | 0,0231 | 0,7109 | 0,0207 |
| **L-Caudate** | **L-MGN** | **RD** | 0,5430 | 0,0198 | 0,5455 | 0,0188 | 0,5421 | 0,0211 | 0,5420 | 0,0196 | 0,5453 | 0,0234 | 0,5412 | 0,0162 | 0,5427 | 0,0231 | 0,5453 | 0,0194 | 0,5461 | 0,0242 | 0,5493 | 0,0225 |
| **L-Caudate** | **L-MGN** | **AD** | 1,0210 | 0,0440 | 1,0308 | 0,0476 | 1,0377 | 0,0440 | 1,0410 | 0,0410 | 1,0176 | 0,0566 | 1,0229 | 0,0453 | 1,0241 | 0,0595 | 1,0346 | 0,0501 | 1,0323 | 0,0549 | 1,0340 | 0,0456 |
| **L-ITC** | **L-MGN** | **MD** | 0,7696 | 0,0214 | 0,7700 | 0,0269 | 0,7734 | 0,0271 | 0,7663 | 0,0251 | 0,7752 | 0,0271 | 0,7694 | 0,0256 | 0,7729 | 0,0254 | 0,7691 | 0,0252 | 0,7720 | 0,0291 | 0,7710 | 0,0305 |
| **L-ITC** | **L-MGN** | **RD** | 0,5705 | 0,0333 | 0,5746 | 0,0297 | 0,5844 | 0,0202 | 0,5692 | 0,0309 | 0,5786 | 0,0330 | 0,5698 | 0,0265 | 0,5772 | 0,0272 | 0,5704 | 0,0304 | 0,5760 | 0,0290 | 0,5767 | 0,0310 |
| **L-ITC** | **L-MGN** | **AD** | 1,1677 | 0,0322 | 1,1537 | 0,0428 | 1,1538 | 0,0459 | 1,1529 | 0,0384 | 1,1617 | 0,0467 | 1,1611 | 0,0433 | 1,1575 | 0,0433 | 1,1590 | 0,0355 | 1,1649 | 0,0366 | 1,1606 | 0,0347 |
| **L-SPC** | **L-MGN** | **MD** | 0,7513 | 0,0186 | 0,7515 | 0,0259 | 0,7503 | 0,0280 | 0,7454 | 0,0197 | 0,7567 | 0,0254 | 0,7508 | 0,0228 | 0,7522 | 0,0227 | 0,7531 | 0,0166 | 0,7543 | 0,0255 | 0,7550 | 0,0264 |
| **L-SPC** | **L-MGN** | **RD** | 0,5622 | 0,0184 | 0,5646 | 0,0251 | 0,5621 | 0,0293 | 0,5588 | 0,0197 | 0,5673 | 0,0249 | 0,5595 | 0,0236 | 0,5636 | 0,0230 | 0,5665 | 0,0174 | 0,5626 | 0,0263 | 0,5649 | 0,0263 |
| **L-SPC** | **L-MGN** | **AD** | 1,1297 | 0,0313 | 1,1253 | 0,0323 | 1,1265 | 0,0355 | 1,1186 | 0,0259 | 1,1357 | 0,0338 | 1,1335 | 0,0295 | 1,1294 | 0,0323 | 1,1263 | 0,0228 | 1,1376 | 0,0324 | 1,1351 | 0,0319 |
| **L-Putamen** | **L-MGN** | **MD** | 0,7220 | 0,0183 | 0,7275 | 0,0276 | 0,7253 | 0,0285 | 0,7213 | 0,0246 | 0,7213 | 0,0296 | 0,7269 | 0,0245 | 0,7215 | 0,0316 | 0,7248 | 0,0261 | 0,7300 | 0,0221 | 0,7246 | 0,0238 |
| **L-Putamen** | **L-MGN** | **RD** | 0,5237 | 0,0170 | 0,5297 | 0,0258 | 0,5295 | 0,0264 | 0,5250 | 0,0268 | 0,5260 | 0,0264 | 0,5285 | 0,0230 | 0,5268 | 0,0274 | 0,5271 | 0,0250 | 0,5312 | 0,0236 | 0,5275 | 0,0249 |
| **L-Putamen** | **L-MGN** | **AD** | 1,1186 | 0,0363 | 1,1231 | 0,0375 | 1,1170 | 0,0392 | 1,1140 | 0,0336 | 1,1121 | 0,0469 | 1,1236 | 0,0377 | 1,1110 | 0,0491 | 1,1201 | 0,0397 | 1,1278 | 0,0340 | 1,1186 | 0,0377 |
| **L-Anterior** | **L-MGN** | **MD** | 0,7366 | 0,0330 | 0,7424 | 0,0370 | 0,7454 | 0,0348 | 0,7407 | 0,0301 | 0,7418 | 0,0387 | 0,7415 | 0,0363 | 0,7451 | 0,0413 | 0,7383 | 0,0346 | 0,7476 | 0,0332 | 0,7463 | 0,0324 |
| **L-Anterior** | **L-MGN** | **RD** | 0,5757 | 0,0341 | 0,5798 | 0,0388 | 0,5832 | 0,0385 | 0,5798 | 0,0338 | 0,5865 | 0,0363 | 0,5815 | 0,0378 | 0,5819 | 0,0424 | 0,5795 | 0,0354 | 0,5801 | 0,0404 | 0,5830 | 0,0390 |
| **L-Anterior** | **L-MGN** | **AD** | 1,0583 | 0,0655 | 1,0675 | 0,0543 | 1,0698 | 0,0622 | 1,0625 | 0,0607 | 1,0523 | 0,0748 | 1,0617 | 0,0604 | 1,0715 | 0,0797 | 1,0558 | 0,0755 | 1,0826 | 0,0539 | 1,0728 | 0,0600 |
| **L-LOC** | **L-MGN** | **MD** | 0,7706 | 0,0188 | 0,7715 | 0,0242 | 0,7709 | 0,0255 | 0,7620 | 0,0206 | 0,7704 | 0,0237 | 0,7683 | 0,0229 | 0,7657 | 0,0250 | 0,7698 | 0,0198 | 0,7700 | 0,0235 | 0,7688 | 0,0224 |
| **L-LOC** | **L-MGN** | **RD** | 0,5536 | 0,0210 | 0,5599 | 0,0247 | 0,5588 | 0,0288 | 0,5528 | 0,0225 | 0,5589 | 0,0248 | 0,5552 | 0,0244 | 0,5547 | 0,0264 | 0,5577 | 0,0212 | 0,5531 | 0,0274 | 0,5529 | 0,0281 |
| **L-LOC** | **L-MGN** | **AD** | 1,2046 | 0,0307 | 1,1947 | 0,0335 | 1,1951 | 0,0365 | 1,1805 | 0,0295 | 1,1934 | 0,0376 | 1,1946 | 0,0349 | 1,1879 | 0,0421 | 1,1939 | 0,0303 | 1,2038 | 0,0315 | 1,2008 | 0,0331 |
| **L-TP** | **L-MGN** | **MD** | 0,8005 | 0,0226 | 0,8007 | 0,0316 | 0,8037 | 0,0343 | 0,7992 | 0,0271 | 0,8111 | 0,0299 | 0,8000 | 0,0301 | 0,8081 | 0,0285 | 0,8059 | 0,0307 | 0,8040 | 0,0332 | 0,8045 | 0,0328 |
| **L-TP** | **L-MGN** | **RD** | 0,6263 | 0,0365 | 0,6267 | 0,0366 | 0,6319 | 0,0410 | 0,6268 | 0,0364 | 0,6388 | 0,0389 | 0,6201 | 0,0395 | 0,6314 | 0,0316 | 0,6315 | 0,0363 | 0,6256 | 0,0463 | 0,6310 | 0,0421 |
| **L-TP** | **L-MGN** | **AD** | 1,1491 | 0,0416 | 1,1487 | 0,0485 | 1,1474 | 0,0457 | 1,1439 | 0,0450 | 1,1557 | 0,0508 | 1,1597 | 0,0463 | 1,1615 | 0,0453 | 1,1546 | 0,0497 | 1,1607 | 0,0429 | 1,1515 | 0,0429 |
| **L-VLD** | **L-MGN** | **MD** | 0,7095 | 0,0267 | 0,7140 | 0,0280 | 0,7167 | 0,0285 | 0,7055 | 0,0217 | 0,7161 | 0,0302 | 0,7123 | 0,0229 | 0,7158 | 0,0265 | 0,7086 | 0,0223 | 0,7186 | 0,0278 | 0,7196 | 0,0246 |
| **L-VLD** | **L-MGN** | **RD** | 0,5656 | 0,0244 | 0,5682 | 0,0229 | 0,5631 | 0,0294 | 0,5578 | 0,0256 | 0,5705 | 0,0272 | 0,5659 | 0,0208 | 0,5703 | 0,0224 | 0,5630 | 0,0219 | 0,5684 | 0,0312 | 0,5696 | 0,0300 |
| **L-VLD** | **L-MGN** | **AD** | 0,9973 | 0,0585 | 1,0054 | 0,0660 | 1,0240 | 0,0608 | 1,0010 | 0,0593 | 1,0074 | 0,0742 | 1,0051 | 0,0632 | 1,0070 | 0,0800 | 0,9999 | 0,0614 | 1,0188 | 0,0678 | 1,0198 | 0,0606 |
| **L-lingual** | **L-MGN** | **MD** | 0,7714 | 0,0269 | 0,7775 | 0,0275 | 0,7730 | 0,0289 | 0,7686 | 0,0261 | 0,7701 | 0,0291 | 0,7736 | 0,0294 | 0,7710 | 0,0286 | 0,7748 | 0,0259 | 0,7762 | 0,0247 | 0,7736 | 0,0260 |
| **L-lingual** | **L-MGN** | **RD** | 0,5603 | 0,0290 | 0,5712 | 0,0302 | 0,5712 | 0,0307 | 0,5614 | 0,0287 | 0,5603 | 0,0294 | 0,5662 | 0,0312 | 0,5613 | 0,0275 | 0,5672 | 0,0303 | 0,5664 | 0,0286 | 0,5651 | 0,0293 |
| **L-lingual** | **L-MGN** | **AD** | 1,1934 | 0,0510 | 1,1903 | 0,0427 | 1,1767 | 0,0474 | 1,1830 | 0,0501 | 1,1897 | 0,0462 | 1,1885 | 0,0591 | 1,1904 | 0,0543 | 1,1901 | 0,0345 | 1,1958 | 0,0480 | 1,1905 | 0,0513 |
| **L-fusiform** | **L-MGN** | **MD** | 0,7754 | 0,0181 | 0,7768 | 0,0300 | 0,7779 | 0,0306 | 0,7748 | 0,0291 | 0,7796 | 0,0268 | 0,7759 | 0,0270 | 0,7766 | 0,0280 | 0,7781 | 0,0289 | 0,7763 | 0,0289 | 0,7762 | 0,0293 |
| **L-fusiform** | **L-MGN** | **RD** | 0,5754 | 0,0328 | 0,5781 | 0,0390 | 0,5835 | 0,0381 | 0,5825 | 0,0355 | 0,5859 | 0,0395 | 0,5778 | 0,0352 | 0,5822 | 0,0363 | 0,5825 | 0,0385 | 0,5761 | 0,0372 | 0,5770 | 0,0380 |
| **L-fusiform** | **L-MGN** | **AD** | 1,1754 | 0,0418 | 1,1741 | 0,0405 | 1,1669 | 0,0417 | 1,1595 | 0,0333 | 1,1671 | 0,0500 | 1,1721 | 0,0407 | 1,1654 | 0,0409 | 1,1693 | 0,0373 | 1,1765 | 0,0324 | 1,1746 | 0,0373 |
| **L-SOC-CN** | **L-MGN** | **MD** | 0,6771 | 0,0243 | 0,6696 | 0,0246 | 0,6673 | 0,0259 | 0,6787 | 0,0274 | 0,6783 | 0,0240 | 0,6704 | 0,0228 | 0,6802 | 0,0249 | 0,6792 | 0,0250 | 0,6792 | 0,0244 | 0,6834 | 0,0241 |
| **L-SOC-CN** | **L-MGN** | **RD** | 0,4924 | 0,0214 | 0,4867 | 0,0288 | 0,4855 | 0,0253 | 0,4908 | 0,0243 | 0,4957 | 0,0241 | 0,4878 | 0,0281 | 0,4967 | 0,0276 | 0,4926 | 0,0243 | 0,4976 | 0,0240 | 0,4984 | 0,0219 |
| **L-SOC-CN** | **L-MGN** | **AD** | 1,0520 | 0,0508 | 1,0375 | 0,0345 | 1,0363 | 0,0427 | 1,0592 | 0,0503 | 1,0474 | 0,0410 | 1,0371 | 0,0338 | 1,0507 | 0,0371 | 1,0529 | 0,0471 | 1,0492 | 0,0444 | 1,0562 | 0,0464 |
| **L-entorhinal** | **L-MGN** | **MD** | 0,7807 | 0,0285 | 0,7735 | 0,0403 | 0,7709 | 0,0397 | 0,7814 | 0,0295 | 0,7785 | 0,0390 | 0,7796 | 0,0329 | 0,7834 | 0,0329 | 0,7810 | 0,0374 | 0,7745 | 0,0385 | 0,7772 | 0,0384 |
| **L-entorhinal** | **L-MGN** | **RD** | 0,6122 | 0,0368 | 0,6034 | 0,0451 | 0,6009 | 0,0463 | 0,6200 | 0,0299 | 0,6118 | 0,0458 | 0,6075 | 0,0443 | 0,6183 | 0,0324 | 0,6143 | 0,0375 | 0,6011 | 0,0540 | 0,6047 | 0,0519 |
| **L-entorhinal** | **L-MGN** | **AD** | 1,1178 | 0,0509 | 1,1208 | 0,0644 | 1,1178 | 0,0659 | 1,1044 | 0,0488 | 1,1190 | 0,0765 | 1,1239 | 0,0650 | 1,1137 | 0,0655 | 1,1217 | 0,0570 | 1,1288 | 0,0649 | 1,1221 | 0,0598 |
| **L-Hippo_CA1** | **L-MGN** | **MD** | 0,7575 | 0,0405 | 0,7663 | 0,0539 | 0,7556 | 0,0420 | 0,7714 | 0,0537 | 0,7591 | 0,0403 | 0,7549 | 0,0302 | 0,7700 | 0,0512 | 0,7752 | 0,0540 | 0,7766 | 0,0679 | 0,7655 | 0,0405 |
| **L-Hippo_CA1** | **L-MGN** | **RD** | 0,5725 | 0,0524 | 0,5747 | 0,0654 | 0,5645 | 0,0557 | 0,5816 | 0,0614 | 0,5638 | 0,0530 | 0,5623 | 0,0470 | 0,5802 | 0,0574 | 0,5891 | 0,0610 | 0,5827 | 0,0788 | 0,5686 | 0,0564 |
| **L-Hippo_CA1** | **L-MGN** | **AD** | 1,1276 | 0,0535 | 1,1495 | 0,0572 | 1,1380 | 0,0573 | 1,1510 | 0,0600 | 1,1497 | 0,0535 | 1,1401 | 0,0461 | 1,1497 | 0,0600 | 1,1473 | 0,0519 | 1,1643 | 0,0726 | 1,1592 | 0,0423 |
| **L-Amygdala** | **L-MGN** | **MD** | 0,7747 | 0,0243 | 0,7778 | 0,0264 | 0,7778 | 0,0303 | 0,7744 | 0,0232 | 0,7817 | 0,0241 | 0,7741 | 0,0222 | 0,7811 | 0,0223 | 0,7788 | 0,0227 | 0,7759 | 0,0278 | 0,7773 | 0,0273 |
| **L-Amygdala** | **L-MGN** | **RD** | 0,5952 | 0,0403 | 0,5922 | 0,0366 | 0,5986 | 0,0411 | 0,5911 | 0,0337 | 0,5998 | 0,0389 | 0,5886 | 0,0369 | 0,5979 | 0,0341 | 0,5976 | 0,0340 | 0,5903 | 0,0422 | 0,5915 | 0,0416 |
| **L-Amygdala** | **L-MGN** | **AD** | 1,1337 | 0,0382 | 1,1490 | 0,0339 | 1,1360 | 0,0378 | 1,1411 | 0,0275 | 1,1455 | 0,0433 | 1,1451 | 0,0415 | 1,1475 | 0,0390 | 1,1411 | 0,0409 | 1,1471 | 0,0412 | 1,1490 | 0,0407 |
| **L-precuneus** | **L-MGN** | **MD** | 0,7617 | 0,0264 | 0,7613 | 0,0286 | 0,7607 | 0,0264 | 0,7530 | 0,0228 | 0,7613 | 0,0264 | 0,7631 | 0,0272 | 0,7609 | 0,0274 | 0,7586 | 0,0230 | 0,7633 | 0,0252 | 0,7634 | 0,0233 |
| **L-precuneus** | **L-MGN** | **RD** | 0,5750 | 0,0226 | 0,5738 | 0,0288 | 0,5752 | 0,0260 | 0,5717 | 0,0235 | 0,5759 | 0,0243 | 0,5744 | 0,0254 | 0,5773 | 0,0258 | 0,5733 | 0,0250 | 0,5771 | 0,0244 | 0,5764 | 0,0232 |
| **L-precuneus** | **L-MGN** | **AD** | 1,1298 | 0,0422 | 1,1311 | 0,0377 | 1,1267 | 0,0382 | 1,1155 | 0,0337 | 1,1320 | 0,0383 | 1,1359 | 0,0370 | 1,1282 | 0,0378 | 1,1239 | 0,0307 | 1,1357 | 0,0352 | 1,1324 | 0,0339 |
| **L-IPC** | **L-MGN** | **MD** | 0,7684 | 0,0244 | 0,7696 | 0,0291 | 0,7698 | 0,0316 | 0,7656 | 0,0280 | 0,7734 | 0,0291 | 0,7673 | 0,0253 | 0,7677 | 0,0261 | 0,7731 | 0,0247 | 0,7737 | 0,0257 | 0,7710 | 0,0280 |
| **L-IPC** | **L-MGN** | **RD** | 0,5726 | 0,0278 | 0,5768 | 0,0302 | 0,5772 | 0,0330 | 0,5723 | 0,0334 | 0,5773 | 0,0313 | 0,5725 | 0,0259 | 0,5720 | 0,0283 | 0,5767 | 0,0295 | 0,5771 | 0,0277 | 0,5732 | 0,0318 |
| **L-IPC** | **L-MGN** | **AD** | 1,1598 | 0,0273 | 1,1551 | 0,0346 | 1,1550 | 0,0344 | 1,1522 | 0,0255 | 1,1655 | 0,0344 | 1,1569 | 0,0308 | 1,1592 | 0,0343 | 1,1660 | 0,0275 | 1,1669 | 0,0271 | 1,1666 | 0,0284 |
| **R-Pallidum** | **L-MGN** | **MD** | 0,6988 | 0,0269 | 0,6887 | 0,0301 | 0,6971 | 0,0268 | 0,6944 | 0,0299 | 0,6960 | 0,0289 | 0,7017 | 0,0295 | 0,6990 | 0,0349 | 0,6872 | 0,0310 | 0,7015 | 0,0283 | 0,6962 | 0,0282 |
| **R-Pallidum** | **L-MGN** | **RD** | 0,4873 | 0,0250 | 0,4737 | 0,0299 | 0,4857 | 0,0222 | 0,4794 | 0,0292 | 0,4833 | 0,0211 | 0,4855 | 0,0243 | 0,4859 | 0,0291 | 0,4782 | 0,0315 | 0,4898 | 0,0237 | 0,4845 | 0,0187 |
| **R-Pallidum** | **L-MGN** | **AD** | 1,1316 | 0,0621 | 1,1147 | 0,0651 | 1,1164 | 0,0651 | 1,1284 | 0,0766 | 1,1259 | 0,0637 | 1,1299 | 0,0793 | 1,1298 | 0,0756 | 1,1136 | 0,0620 | 1,1340 | 0,0644 | 1,1277 | 0,0714 |
| **L-Ventral_Anterior** | **L-MGN** | **MD** | 0,7246 | 0,0253 | 0,7243 | 0,0291 | 0,7259 | 0,0296 | 0,7254 | 0,0255 | 0,7284 | 0,0297 | 0,7238 | 0,0247 | 0,7321 | 0,0252 | 0,7261 | 0,0245 | 0,7308 | 0,0262 | 0,7324 | 0,0238 |
| **L-Ventral_Anterior** | **L-MGN** | **RD** | 0,5889 | 0,0231 | 0,5835 | 0,0289 | 0,5797 | 0,0348 | 0,5810 | 0,0310 | 0,5915 | 0,0291 | 0,5853 | 0,0279 | 0,5907 | 0,0262 | 0,5877 | 0,0285 | 0,5883 | 0,0315 | 0,5879 | 0,0307 |
| **L-Ventral_Anterior** | **L-MGN** | **AD** | 0,9959 | 0,0837 | 1,0059 | 0,0800 | 1,0183 | 0,0837 | 1,0142 | 0,0662 | 1,0023 | 0,0861 | 1,0008 | 0,0838 | 1,0147 | 0,0886 | 1,0028 | 0,0827 | 1,0160 | 0,0907 | 1,0213 | 0,0752 |
| **L-MGN** | **L-IC** | **MD** | 0,7258 | 0,0128 | 0,7204 | 0,0210 | 0,7187 | 0,0201 | 0,7224 | 0,0208 | 0,7202 | 0,0181 | 0,7232 | 0,0182 | 0,7188 | 0,0218 | 0,7202 | 0,0185 | 0,7248 | 0,0154 | 0,7268 | 0,0192 |
| **L-MGN** | **L-IC** | **RD** | 0,5184 | 0,0112 | 0,5132 | 0,0195 | 0,5141 | 0,0179 | 0,5153 | 0,0178 | 0,5160 | 0,0148 | 0,5162 | 0,0181 | 0,5157 | 0,0184 | 0,5125 | 0,0157 | 0,5168 | 0,0149 | 0,5193 | 0,0195 |
| **L-MGN** | **L-IC** | **AD** | 1,1408 | 0,0319 | 1,1348 | 0,0295 | 1,1280 | 0,0344 | 1,1366 | 0,0341 | 1,1339 | 0,0350 | 1,1372 | 0,0316 | 1,1301 | 0,0360 | 1,1406 | 0,0330 | 1,1408 | 0,0274 | 1,1475 | 0,0264 |
| **L-Putamen** | **L-IC** | **MD** | 0,7133 | 0,0274 | 0,7137 | 0,0370 | 0,7142 | 0,0465 | 0,7162 | 0,0397 | 0,7068 | 0,0383 | 0,7192 | 0,0378 | 0,7130 | 0,0455 | 0,7132 | 0,0370 | 0,7162 | 0,0328 | 0,7186 | 0,0300 |
| **L-Putamen** | **L-IC** | **RD** | 0,5298 | 0,0314 | 0,5238 | 0,0343 | 0,5249 | 0,0384 | 0,5289 | 0,0372 | 0,5238 | 0,0351 | 0,5290 | 0,0365 | 0,5324 | 0,0393 | 0,5282 | 0,0340 | 0,5286 | 0,0294 | 0,5302 | 0,0304 |
| **L-Putamen** | **L-IC** | **AD** | 1,0803 | 0,0407 | 1,0936 | 0,0638 | 1,0928 | 0,0721 | 1,0908 | 0,0645 | 1,0729 | 0,0576 | 1,0995 | 0,0602 | 1,0743 | 0,0669 | 1,0832 | 0,0645 | 1,0914 | 0,0559 | 1,0956 | 0,0540 |
| **L-SPC** | **L-IC** | **MD** | 0,7508 | 0,0243 | 0,7475 | 0,0316 | 0,7500 | 0,0329 | 0,7411 | 0,0247 | 0,7555 | 0,0312 | 0,7447 | 0,0299 | 0,7500 | 0,0251 | 0,7545 | 0,0196 | 0,7516 | 0,0315 | 0,7533 | 0,0306 |
| **L-SPC** | **L-IC** | **RD** | 0,5594 | 0,0263 | 0,5659 | 0,0299 | 0,5651 | 0,0325 | 0,5542 | 0,0266 | 0,5670 | 0,0302 | 0,5598 | 0,0295 | 0,5622 | 0,0268 | 0,5658 | 0,0251 | 0,5633 | 0,0299 | 0,5628 | 0,0325 |
| **L-SPC** | **L-IC** | **AD** | 1,1337 | 0,0435 | 1,1108 | 0,0401 | 1,1198 | 0,0445 | 1,1148 | 0,0430 | 1,1326 | 0,0462 | 1,1145 | 0,0411 | 1,1255 | 0,0377 | 1,1318 | 0,0341 | 1,1280 | 0,0443 | 1,1342 | 0,0431 |
| **L-SOC-CN** | **L-IC** | **MD** | 0,7182 | 0,0202 | 0,7123 | 0,0230 | 0,7116 | 0,0242 | 0,7147 | 0,0235 | 0,7210 | 0,0188 | 0,7168 | 0,0196 | 0,7170 | 0,0205 | 0,7113 | 0,0216 | 0,7171 | 0,0228 | 0,7197 | 0,0211 |
| **L-SOC-CN** | **L-IC** | **RD** | 0,5376 | 0,0227 | 0,5325 | 0,0229 | 0,5354 | 0,0249 | 0,5343 | 0,0239 | 0,5406 | 0,0218 | 0,5377 | 0,0240 | 0,5369 | 0,0219 | 0,5317 | 0,0229 | 0,5376 | 0,0221 | 0,5396 | 0,0215 |
| **L-SOC-CN** | **L-IC** | **AD** | 1,0795 | 0,0284 | 1,0737 | 0,0316 | 1,0653 | 0,0333 | 1,0755 | 0,0294 | 1,0819 | 0,0285 | 1,0748 | 0,0255 | 1,0773 | 0,0297 | 1,0723 | 0,0295 | 1,0779 | 0,0323 | 1,0799 | 0,0283 |
| **L-VLD** | **L-IC** | **MD** | 0,6826 | 0,0166 | 0,6811 | 0,0261 | 0,6836 | 0,0241 | 0,6800 | 0,0200 | 0,6889 | 0,0230 | 0,6832 | 0,0270 | 0,6809 | 0,0145 | 0,6810 | 0,0198 | 0,6880 | 0,0219 | 0,6894 | 0,0231 |
| **L-VLD** | **L-IC** | **RD** | 0,5456 | 0,0177 | 0,5449 | 0,0198 | 0,5425 | 0,0234 | 0,5425 | 0,0157 | 0,5476 | 0,0199 | 0,5444 | 0,0217 | 0,5444 | 0,0178 | 0,5448 | 0,0185 | 0,5447 | 0,0236 | 0,5484 | 0,0223 |
| **L-VLD** | **L-IC** | **AD** | 0,9565 | 0,0500 | 0,9536 | 0,0511 | 0,9658 | 0,0560 | 0,9551 | 0,0554 | 0,9716 | 0,0576 | 0,9609 | 0,0547 | 0,9539 | 0,0450 | 0,9533 | 0,0519 | 0,9747 | 0,0549 | 0,9712 | 0,0611 |
| **L-Pallidum** | **L-IC** | **MD** | 0,6963 | 0,0234 | 0,6941 | 0,0330 | 0,6952 | 0,0356 | 0,6918 | 0,0285 | 0,6905 | 0,0313 | 0,6949 | 0,0248 | 0,6882 | 0,0312 | 0,6974 | 0,0329 | 0,7017 | 0,0322 | 0,6986 | 0,0242 |
| **L-Pallidum** | **L-IC** | **RD** | 0,5185 | 0,0272 | 0,5130 | 0,0261 | 0,5132 | 0,0293 | 0,5123 | 0,0267 | 0,5087 | 0,0257 | 0,5132 | 0,0229 | 0,5087 | 0,0260 | 0,5138 | 0,0314 | 0,5183 | 0,0278 | 0,5113 | 0,0277 |
| **L-Pallidum** | **L-IC** | **AD** | 1,0519 | 0,0431 | 1,0489 | 0,0579 | 1,0522 | 0,0549 | 1,0510 | 0,0565 | 1,0542 | 0,0604 | 1,0583 | 0,0550 | 1,0474 | 0,0613 | 1,0581 | 0,0487 | 1,0618 | 0,0534 | 1,0731 | 0,0477 |
| **L-parstr** | **L-PAC** | **MD** | 0,7630 | 0,0202 | 0,7638 | 0,0276 | 0,7658 | 0,0278 | 0,7618 | 0,0241 | 0,7651 | 0,0267 | 0,7626 | 0,0268 | 0,7618 | 0,0243 | 0,7679 | 0,0185 | 0,7690 | 0,0247 | 0,7670 | 0,0243 |
| **L-parstr** | **L-PAC** | **RD** | 0,5779 | 0,0227 | 0,5773 | 0,0314 | 0,5799 | 0,0311 | 0,5755 | 0,0264 | 0,5803 | 0,0310 | 0,5756 | 0,0293 | 0,5792 | 0,0266 | 0,5811 | 0,0222 | 0,5866 | 0,0263 | 0,5836 | 0,0255 |
| **L-parstr** | **L-PAC** | **AD** | 1,1333 | 0,0263 | 1,1368 | 0,0303 | 1,1378 | 0,0334 | 1,1344 | 0,0325 | 1,1348 | 0,0277 | 1,1365 | 0,0321 | 1,1270 | 0,0276 | 1,1417 | 0,0259 | 1,1339 | 0,0292 | 1,1339 | 0,0313 |
| **L-TP** | **L-PAC** | **MD** | 0,8096 | 0,0270 | 0,8111 | 0,0317 | 0,8168 | 0,0309 | 0,8065 | 0,0271 | 0,8146 | 0,0316 | 0,8098 | 0,0296 | 0,8132 | 0,0307 | 0,8147 | 0,0309 | 0,8154 | 0,0309 | 0,8106 | 0,0307 |
| **L-TP** | **L-PAC** | **RD** | 0,6153 | 0,0304 | 0,6183 | 0,0308 | 0,6239 | 0,0319 | 0,6131 | 0,0304 | 0,6236 | 0,0319 | 0,6144 | 0,0318 | 0,6212 | 0,0287 | 0,6189 | 0,0332 | 0,6206 | 0,0354 | 0,6180 | 0,0347 |
| **L-TP** | **L-PAC** | **AD** | 1,1936 | 0,0328 | 1,1920 | 0,0382 | 1,1980 | 0,0384 | 1,1933 | 0,0353 | 1,1869 | 0,0355 | 1,1963 | 0,0345 | 1,1876 | 0,0367 | 1,1976 | 0,0342 | 1,2008 | 0,0340 | 1,1911 | 0,0318 |
| **L-Accumbens** | **L-PAC** | **MD** | 0,7866 | 0,0237 | 0,7868 | 0,0347 | 0,7907 | 0,0349 | 0,7828 | 0,0289 | 0,7931 | 0,0328 | 0,7838 | 0,0310 | 0,7855 | 0,0315 | 0,7905 | 0,0276 | 0,7947 | 0,0311 | 0,7882 | 0,0285 |
| **L-Accumbens** | **L-PAC** | **RD** | 0,5828 | 0,0247 | 0,5819 | 0,0306 | 0,5851 | 0,0337 | 0,5807 | 0,0297 | 0,5911 | 0,0335 | 0,5770 | 0,0276 | 0,5857 | 0,0272 | 0,5817 | 0,0286 | 0,5912 | 0,0353 | 0,5835 | 0,0296 |
| **L-Accumbens** | **L-PAC** | **AD** | 1,1941 | 0,0396 | 1,1965 | 0,0587 | 1,2018 | 0,0540 | 1,1872 | 0,0431 | 1,1972 | 0,0529 | 1,1974 | 0,0567 | 1,1851 | 0,0557 | 1,2082 | 0,0381 | 1,2016 | 0,0474 | 1,1975 | 0,0461 |
| **L-Amygdala** | **L-PAC** | **MD** | 0,7835 | 0,0211 | 0,7844 | 0,0320 | 0,7876 | 0,0308 | 0,7796 | 0,0266 | 0,7862 | 0,0268 | 0,7808 | 0,0257 | 0,7834 | 0,0287 | 0,7866 | 0,0239 | 0,7890 | 0,0255 | 0,7845 | 0,0248 |
| **L-Amygdala** | **L-PAC** | **RD** | 0,5730 | 0,0250 | 0,5749 | 0,0299 | 0,5777 | 0,0310 | 0,5713 | 0,0295 | 0,5765 | 0,0282 | 0,5697 | 0,0280 | 0,5773 | 0,0269 | 0,5775 | 0,0246 | 0,5778 | 0,0273 | 0,5776 | 0,0256 |
| **L-Amygdala** | **L-PAC** | **AD** | 1,2046 | 0,0300 | 1,2034 | 0,0394 | 1,2075 | 0,0393 | 1,1960 | 0,0279 | 1,2057 | 0,0359 | 1,2029 | 0,0326 | 1,1958 | 0,0428 | 1,2047 | 0,0328 | 1,2112 | 0,0352 | 1,1984 | 0,0337 |
| **L-LOC** | **L-PAC** | **MD** | 0,7890 | 0,0209 | 0,7875 | 0,0270 | 0,7888 | 0,0275 | 0,7798 | 0,0244 | 0,7855 | 0,0256 | 0,7844 | 0,0246 | 0,7845 | 0,0255 | 0,7903 | 0,0223 | 0,7853 | 0,0257 | 0,7871 | 0,0258 |
| **L-LOC** | **L-PAC** | **RD** | 0,5740 | 0,0236 | 0,5741 | 0,0276 | 0,5791 | 0,0244 | 0,5699 | 0,0270 | 0,5760 | 0,0275 | 0,5694 | 0,0253 | 0,5752 | 0,0248 | 0,5796 | 0,0232 | 0,5744 | 0,0231 | 0,5771 | 0,0225 |
| **L-LOC** | **L-PAC** | **AD** | 1,2192 | 0,0326 | 1,2142 | 0,0438 | 1,2192 | 0,0387 | 1,1998 | 0,0356 | 1,2046 | 0,0397 | 1,2143 | 0,0400 | 1,2032 | 0,0433 | 1,2116 | 0,0399 | 1,2175 | 0,0378 | 1,2179 | 0,0393 |
| **L-lOFC** | **L-PAC** | **MD** | 0,7754 | 0,0235 | 0,7789 | 0,0313 | 0,7810 | 0,0301 | 0,7760 | 0,0265 | 0,7815 | 0,0307 | 0,7747 | 0,0282 | 0,7789 | 0,0287 | 0,7820 | 0,0233 | 0,7823 | 0,0271 | 0,7806 | 0,0283 |
| **L-lOFC** | **L-PAC** | **RD** | 0,5751 | 0,0287 | 0,5779 | 0,0353 | 0,5812 | 0,0316 | 0,5774 | 0,0333 | 0,5866 | 0,0342 | 0,5716 | 0,0304 | 0,5824 | 0,0332 | 0,5811 | 0,0298 | 0,5848 | 0,0314 | 0,5811 | 0,0342 |
| **L-lOFC** | **L-PAC** | **AD** | 1,1759 | 0,0374 | 1,1809 | 0,0423 | 1,1805 | 0,0449 | 1,1733 | 0,0294 | 1,1713 | 0,0413 | 1,1807 | 0,0422 | 1,1719 | 0,0362 | 1,1839 | 0,0375 | 1,1772 | 0,0330 | 1,1797 | 0,0425 |
| **L-parso** | **L-PAC** | **MD** | 0,7637 | 0,0216 | 0,7650 | 0,0299 | 0,7667 | 0,0293 | 0,7619 | 0,0267 | 0,7668 | 0,0286 | 0,7653 | 0,0274 | 0,7649 | 0,0274 | 0,7665 | 0,0232 | 0,7687 | 0,0282 | 0,7706 | 0,0275 |
| **L-parso** | **L-PAC** | **RD** | 0,5800 | 0,0241 | 0,5836 | 0,0324 | 0,5844 | 0,0335 | 0,5813 | 0,0280 | 0,5831 | 0,0293 | 0,5861 | 0,0308 | 0,5844 | 0,0298 | 0,5829 | 0,0257 | 0,5875 | 0,0303 | 0,5888 | 0,0322 |
| **L-parso** | **L-PAC** | **AD** | 1,1312 | 0,0301 | 1,1277 | 0,0318 | 1,1314 | 0,0319 | 1,1231 | 0,0434 | 1,1343 | 0,0384 | 1,1238 | 0,0359 | 1,1258 | 0,0424 | 1,1337 | 0,0358 | 1,1311 | 0,0328 | 1,1342 | 0,0398 |
| **L-Hippo_HATA** | **L-PAC** | **MD** | 0,7459 | 0,0298 | 0,7597 | 0,0225 | 0,7503 | 0,0304 | 0,7520 | 0,0244 | 0,7459 | 0,0322 | 0,7453 | 0,0274 | 0,7481 | 0,0248 | 0,7517 | 0,0247 | 0,7529 | 0,0289 | 0,7543 | 0,0267 |
| **L-Hippo_HATA** | **L-PAC** | **RD** | 0,5111 | 0,0401 | 0,5248 | 0,0273 | 0,5180 | 0,0372 | 0,5109 | 0,0340 | 0,5075 | 0,0426 | 0,5101 | 0,0402 | 0,5076 | 0,0366 | 0,5189 | 0,0298 | 0,5204 | 0,0380 | 0,5180 | 0,0336 |
| **L-Hippo_HATA** | **L-PAC** | **AD** | 1,2040 | 0,0486 | 1,2296 | 0,0362 | 1,2150 | 0,0559 | 1,2225 | 0,0345 | 1,2228 | 0,0269 | 1,2159 | 0,0490 | 1,2292 | 0,0350 | 1,2063 | 0,0516 | 1,2179 | 0,0536 | 1,2158 | 0,0386 |
| **L-Putamen** | **L-PAC** | **MD** | 0,7599 | 0,0199 | 0,7610 | 0,0276 | 0,7614 | 0,0292 | 0,7577 | 0,0236 | 0,7613 | 0,0253 | 0,7584 | 0,0236 | 0,7576 | 0,0258 | 0,7649 | 0,0214 | 0,7629 | 0,0248 | 0,7620 | 0,0248 |
| **L-Putamen** | **L-PAC** | **RD** | 0,5674 | 0,0224 | 0,5709 | 0,0280 | 0,5716 | 0,0312 | 0,5689 | 0,0251 | 0,5709 | 0,0284 | 0,5676 | 0,0254 | 0,5705 | 0,0269 | 0,5727 | 0,0242 | 0,5737 | 0,0273 | 0,5716 | 0,0272 |
| **L-Putamen** | **L-PAC** | **AD** | 1,1448 | 0,0305 | 1,1412 | 0,0324 | 1,1409 | 0,0323 | 1,1352 | 0,0314 | 1,1420 | 0,0299 | 1,1400 | 0,0294 | 1,1317 | 0,0336 | 1,1493 | 0,0281 | 1,1412 | 0,0299 | 1,1429 | 0,0340 |
| **R-Pallidum** | **L-PAC** | **MD** | 0,7267 | 0,0314 | 0,7451 | 0,0569 | 0,7384 | 0,0593 | 0,7374 | 0,0651 | 0,7423 | 0,0615 | 0,7415 | 0,0613 | 0,7307 | 0,0369 | 0,7200 | 0,0349 | 0,7289 | 0,0296 | 0,7256 | 0,0346 |
| **R-Pallidum** | **L-PAC** | **RD** | 0,4909 | 0,0378 | 0,5102 | 0,0691 | 0,5123 | 0,0689 | 0,4959 | 0,0723 | 0,5135 | 0,0709 | 0,5077 | 0,0694 | 0,4979 | 0,0402 | 0,4888 | 0,0399 | 0,4989 | 0,0351 | 0,4884 | 0,0356 |
| **R-Pallidum** | **L-PAC** | **AD** | 1,1981 | 0,0539 | 1,2148 | 0,0649 | 1,1907 | 0,0633 | 1,2203 | 0,0857 | 1,1999 | 0,0724 | 1,2091 | 0,0737 | 1,1964 | 0,0724 | 1,1823 | 0,0555 | 1,1891 | 0,0461 | 1,1999 | 0,0725 |
| **L-SOC-CN** | **L-PAC** | **MD** | 0,7186 | 0,0218 | 0,7105 | 0,0225 | 0,7095 | 0,0244 | 0,7156 | 0,0243 | 0,7209 | 0,0220 | 0,7151 | 0,0199 | 0,7176 | 0,0205 | 0,7136 | 0,0231 | 0,7177 | 0,0263 | 0,7201 | 0,0221 |
| **L-SOC-CN** | **L-PAC** | **RD** | 0,5614 | 0,0288 | 0,5538 | 0,0254 | 0,5575 | 0,0265 | 0,5560 | 0,0276 | 0,5630 | 0,0268 | 0,5578 | 0,0273 | 0,5589 | 0,0222 | 0,5571 | 0,0255 | 0,5600 | 0,0267 | 0,5638 | 0,0249 |
| **L-SOC-CN** | **L-PAC** | **AD** | 1,0331 | 0,0384 | 1,0240 | 0,0393 | 1,0135 | 0,0404 | 1,0349 | 0,0399 | 1,0390 | 0,0409 | 1,0297 | 0,0342 | 1,0375 | 0,0402 | 1,0267 | 0,0460 | 1,0331 | 0,0442 | 1,0325 | 0,0421 |
| **L-IPC** | **L-PAC** | **MD** | 0,7929 | 0,0253 | 0,7928 | 0,0319 | 0,7953 | 0,0312 | 0,7877 | 0,0297 | 0,7967 | 0,0311 | 0,7916 | 0,0260 | 0,7914 | 0,0305 | 0,7962 | 0,0277 | 0,7972 | 0,0295 | 0,7947 | 0,0305 |
| **L-IPC** | **L-PAC** | **RD** | 0,5986 | 0,0267 | 0,6020 | 0,0329 | 0,6044 | 0,0331 | 0,5985 | 0,0323 | 0,6051 | 0,0305 | 0,6004 | 0,0278 | 0,6003 | 0,0300 | 0,6035 | 0,0296 | 0,6037 | 0,0330 | 0,5991 | 0,0352 |
| **L-IPC** | **L-PAC** | **AD** | 1,1816 | 0,0304 | 1,1744 | 0,0407 | 1,1772 | 0,0379 | 1,1659 | 0,0319 | 1,1800 | 0,0404 | 1,1740 | 0,0341 | 1,1737 | 0,0419 | 1,1816 | 0,0340 | 1,1842 | 0,0312 | 1,1858 | 0,0310 |
| **L-SFC** | **L-PAC** | **MD** | 0,7531 | 0,0258 | 0,7612 | 0,0268 | 0,7591 | 0,0292 | 0,7554 | 0,0254 | 0,7542 | 0,0329 | 0,7584 | 0,0262 | 0,7579 | 0,0284 | 0,7594 | 0,0262 | 0,7611 | 0,0283 | 0,7590 | 0,0284 |
| **L-SFC** | **L-PAC** | **RD** | 0,5643 | 0,0366 | 0,5740 | 0,0299 | 0,5723 | 0,0329 | 0,5659 | 0,0316 | 0,5642 | 0,0413 | 0,5729 | 0,0311 | 0,5714 | 0,0374 | 0,5706 | 0,0357 | 0,5737 | 0,0338 | 0,5699 | 0,0392 |
| **L-SFC** | **L-PAC** | **AD** | 1,1309 | 0,0207 | 1,1354 | 0,0349 | 1,1326 | 0,0322 | 1,1346 | 0,0309 | 1,1343 | 0,0291 | 1,1293 | 0,0286 | 1,1308 | 0,0259 | 1,1371 | 0,0227 | 1,1360 | 0,0291 | 1,1373 | 0,0237 |
| **L-Ventral_Anterior** | **L-PAC** | **MD** | 0,7813 | 0,0223 | 0,7854 | 0,0270 | 0,7865 | 0,0269 | 0,7789 | 0,0169 | 0,7884 | 0,0257 | 0,7819 | 0,0252 | 0,7861 | 0,0218 | 0,7844 | 0,0214 | 0,7861 | 0,0286 | 0,7823 | 0,0271 |
| **L-Ventral_Anterior** | **L-PAC** | **RD** | 0,5968 | 0,0244 | 0,5994 | 0,0252 | 0,5985 | 0,0314 | 0,5919 | 0,0184 | 0,6038 | 0,0276 | 0,5977 | 0,0267 | 0,6013 | 0,0210 | 0,5972 | 0,0230 | 0,6047 | 0,0310 | 0,5957 | 0,0319 |
| **L-Ventral_Anterior** | **L-PAC** | **AD** | 1,1503 | 0,0368 | 1,1575 | 0,0383 | 1,1625 | 0,0364 | 1,1530 | 0,0269 | 1,1575 | 0,0406 | 1,1503 | 0,0407 | 1,1558 | 0,0383 | 1,1587 | 0,0355 | 1,1489 | 0,0418 | 1,1555 | 0,0334 |
| **R-Putamen** | **L-PAC** | **MD** | 0,7530 | 0,0389 | 0,7558 | 0,0364 | 0,7560 | 0,0379 | 0,7639 | 0,0410 | 0,7624 | 0,0412 | 0,7544 | 0,0425 | 0,7611 | 0,0387 | 0,7582 | 0,0437 | 0,7582 | 0,0389 | 0,7606 | 0,0434 |
| **R-Putamen** | **L-PAC** | **RD** | 0,5130 | 0,0451 | 0,5189 | 0,0525 | 0,5326 | 0,0512 | 0,5257 | 0,0550 | 0,5310 | 0,0437 | 0,5222 | 0,0589 | 0,5282 | 0,0493 | 0,5235 | 0,0545 | 0,5259 | 0,0441 | 0,5218 | 0,0459 |
| **R-Putamen** | **L-PAC** | **AD** | 1,2328 | 0,0596 | 1,2295 | 0,0469 | 1,2026 | 0,0534 | 1,2403 | 0,0626 | 1,2252 | 0,0674 | 1,2187 | 0,0562 | 1,2270 | 0,0623 | 1,2276 | 0,0608 | 1,2230 | 0,0638 | 1,2380 | 0,0730 |
| **L-rMFC** | **L-PAC** | **MD** | 0,7703 | 0,0226 | 0,7751 | 0,0285 | 0,7756 | 0,0295 | 0,7715 | 0,0212 | 0,7779 | 0,0276 | 0,7709 | 0,0259 | 0,7753 | 0,0258 | 0,7788 | 0,0217 | 0,7770 | 0,0266 | 0,7755 | 0,0254 |
| **L-rMFC** | **L-PAC** | **RD** | 0,5818 | 0,0249 | 0,5863 | 0,0310 | 0,5877 | 0,0317 | 0,5835 | 0,0249 | 0,5898 | 0,0309 | 0,5814 | 0,0297 | 0,5886 | 0,0284 | 0,5864 | 0,0261 | 0,5910 | 0,0294 | 0,5860 | 0,0304 |
| **L-rMFC** | **L-PAC** | **AD** | 1,1472 | 0,0260 | 1,1525 | 0,0323 | 1,1513 | 0,0317 | 1,1475 | 0,0247 | 1,1541 | 0,0313 | 1,1500 | 0,0293 | 1,1486 | 0,0318 | 1,1634 | 0,0269 | 1,1492 | 0,0278 | 1,1546 | 0,0298 |
| **L-Caudate** | **L-PAC** | **MD** | 0,7643 | 0,0192 | 0,7682 | 0,0286 | 0,7715 | 0,0283 | 0,7660 | 0,0235 | 0,7707 | 0,0272 | 0,7644 | 0,0267 | 0,7689 | 0,0232 | 0,7728 | 0,0221 | 0,7720 | 0,0273 | 0,7726 | 0,0274 |
| **L-Caudate** | **L-PAC** | **RD** | 0,5790 | 0,0178 | 0,5811 | 0,0275 | 0,5821 | 0,0300 | 0,5780 | 0,0235 | 0,5864 | 0,0252 | 0,5770 | 0,0253 | 0,5837 | 0,0205 | 0,5829 | 0,0209 | 0,5867 | 0,0277 | 0,5833 | 0,0305 |
| **L-Caudate** | **L-PAC** | **AD** | 1,1350 | 0,0328 | 1,1425 | 0,0417 | 1,1501 | 0,0361 | 1,1420 | 0,0350 | 1,1393 | 0,0415 | 1,1392 | 0,0431 | 1,1393 | 0,0374 | 1,1526 | 0,0374 | 1,1425 | 0,0367 | 1,1514 | 0,0365 |
| **L-ITC** | **L-PAC** | **MD** | 0,7894 | 0,0253 | 0,7891 | 0,0310 | 0,7927 | 0,0324 | 0,7824 | 0,0276 | 0,7908 | 0,0297 | 0,7888 | 0,0287 | 0,7888 | 0,0272 | 0,7907 | 0,0254 | 0,7949 | 0,0278 | 0,7912 | 0,0300 |
| **L-ITC** | **L-PAC** | **RD** | 0,5881 | 0,0290 | 0,5880 | 0,0348 | 0,5960 | 0,0311 | 0,5835 | 0,0327 | 0,5924 | 0,0341 | 0,5881 | 0,0315 | 0,5924 | 0,0287 | 0,5911 | 0,0273 | 0,5998 | 0,0243 | 0,5951 | 0,0300 |
| **L-ITC** | **L-PAC** | **AD** | 1,1920 | 0,0316 | 1,1913 | 0,0371 | 1,1945 | 0,0380 | 1,1800 | 0,0373 | 1,1876 | 0,0361 | 1,1902 | 0,0364 | 1,1815 | 0,0347 | 1,1898 | 0,0355 | 1,1937 | 0,0301 | 1,1918 | 0,0342 |
| **L-SPC** | **L-PAC** | **MD** | 0,7778 | 0,0207 | 0,7770 | 0,0286 | 0,7786 | 0,0300 | 0,7728 | 0,0253 | 0,7810 | 0,0280 | 0,7779 | 0,0264 | 0,7778 | 0,0271 | 0,7798 | 0,0206 | 0,7788 | 0,0267 | 0,7796 | 0,0264 |
| **L-SPC** | **L-PAC** | **RD** | 0,5822 | 0,0227 | 0,5851 | 0,0301 | 0,5854 | 0,0394 | 0,5802 | 0,0255 | 0,5871 | 0,0284 | 0,5851 | 0,0271 | 0,5858 | 0,0280 | 0,5841 | 0,0239 | 0,5816 | 0,0374 | 0,5810 | 0,0371 |
| **L-SPC** | **L-PAC** | **AD** | 1,1691 | 0,0262 | 1,1646 | 0,0329 | 1,1691 | 0,0303 | 1,1617 | 0,0326 | 1,1731 | 0,0336 | 1,1676 | 0,0289 | 1,1657 | 0,0335 | 1,1711 | 0,0298 | 1,1734 | 0,0310 | 1,1768 | 0,0309 |
| **L-IC** | **L-PAC** | **MD** | 0,7135 | 0,0163 | 0,7095 | 0,0175 | 0,7096 | 0,0172 | 0,7122 | 0,0186 | 0,7137 | 0,0182 | 0,7102 | 0,0126 | 0,7110 | 0,0188 | 0,7114 | 0,0181 | 0,7151 | 0,0194 | 0,7181 | 0,0157 |
| **L-IC** | **L-PAC** | **RD** | 0,5565 | 0,0196 | 0,5500 | 0,0193 | 0,5537 | 0,0219 | 0,5509 | 0,0180 | 0,5548 | 0,0206 | 0,5498 | 0,0173 | 0,5515 | 0,0172 | 0,5508 | 0,0207 | 0,5596 | 0,0171 | 0,5581 | 0,0162 |
| **L-IC** | **L-PAC** | **AD** | 1,0274 | 0,0306 | 1,0285 | 0,0344 | 1,0280 | 0,0313 | 1,0346 | 0,0410 | 1,0354 | 0,0336 | 1,0309 | 0,0302 | 1,0337 | 0,0356 | 1,0324 | 0,0319 | 1,0332 | 0,0348 | 1,0452 | 0,0311 |
| **L-Hippo_CA1** | **L-PAC** | **MD** | 0,7782 | 0,0250 | 0,7786 | 0,0360 | 0,7849 | 0,0384 | 0,7755 | 0,0278 | 0,7830 | 0,0293 | 0,7827 | 0,0300 | 0,7813 | 0,0245 | 0,7761 | 0,0253 | 0,7799 | 0,0329 | 0,7775 | 0,0316 |
| **L-Hippo_CA1** | **L-PAC** | **RD** | 0,5441 | 0,0390 | 0,5531 | 0,0431 | 0,5545 | 0,0513 | 0,5464 | 0,0446 | 0,5454 | 0,0433 | 0,5513 | 0,0483 | 0,5565 | 0,0299 | 0,5487 | 0,0308 | 0,5495 | 0,0365 | 0,5501 | 0,0364 |
| **L-Hippo_CA1** | **L-PAC** | **AD** | 1,2340 | 0,0371 | 1,2412 | 0,0594 | 1,2425 | 0,0598 | 1,2457 | 0,0471 | 1,2539 | 0,0496 | 1,2426 | 0,0550 | 1,2423 | 0,0532 | 1,2391 | 0,0472 | 1,2475 | 0,0552 | 1,2405 | 0,0543 |
| **L-Anterior** | **L-PAC** | **MD** | 0,7751 | 0,0203 | 0,7757 | 0,0295 | 0,7755 | 0,0305 | 0,7716 | 0,0232 | 0,7763 | 0,0282 | 0,7726 | 0,0271 | 0,7747 | 0,0255 | 0,7790 | 0,0223 | 0,7810 | 0,0249 | 0,7776 | 0,0240 |
| **L-Anterior** | **L-PAC** | **RD** | 0,5853 | 0,0228 | 0,5851 | 0,0293 | 0,5848 | 0,0314 | 0,5784 | 0,0281 | 0,5867 | 0,0320 | 0,5811 | 0,0298 | 0,5850 | 0,0269 | 0,5865 | 0,0241 | 0,5917 | 0,0297 | 0,5876 | 0,0293 |
| **L-Anterior** | **L-PAC** | **AD** | 1,1547 | 0,0266 | 1,1568 | 0,0371 | 1,1569 | 0,0337 | 1,1581 | 0,0247 | 1,1556 | 0,0328 | 1,1555 | 0,0335 | 1,1541 | 0,0365 | 1,1642 | 0,0314 | 1,1598 | 0,0287 | 1,1577 | 0,0281 |
| **L-Pallidum** | **L-PAC** | **MD** | 0,7448 | 0,0256 | 0,7532 | 0,0327 | 0,7513 | 0,0336 | 0,7454 | 0,0307 | 0,7449 | 0,0320 | 0,7486 | 0,0299 | 0,7441 | 0,0332 | 0,7504 | 0,0302 | 0,7503 | 0,0300 | 0,7472 | 0,0309 |
| **L-Pallidum** | **L-PAC** | **RD** | 0,5381 | 0,0287 | 0,5513 | 0,0292 | 0,5456 | 0,0333 | 0,5414 | 0,0301 | 0,5376 | 0,0342 | 0,5431 | 0,0296 | 0,5410 | 0,0310 | 0,5421 | 0,0299 | 0,5452 | 0,0295 | 0,5419 | 0,0309 |
| **L-Pallidum** | **L-PAC** | **AD** | 1,1582 | 0,0315 | 1,1569 | 0,0458 | 1,1627 | 0,0438 | 1,1532 | 0,0379 | 1,1595 | 0,0419 | 1,1596 | 0,0405 | 1,1505 | 0,0486 | 1,1670 | 0,0389 | 1,1606 | 0,0388 | 1,1579 | 0,0401 |
| **L-fusiform** | **L-PAC** | **MD** | 0,7929 | 0,0277 | 0,7926 | 0,0310 | 0,7947 | 0,0349 | 0,7846 | 0,0268 | 0,7916 | 0,0326 | 0,7904 | 0,0311 | 0,7906 | 0,0282 | 0,7934 | 0,0246 | 0,7963 | 0,0272 | 0,7940 | 0,0254 |
| **L-fusiform** | **L-PAC** | **RD** | 0,5833 | 0,0295 | 0,5820 | 0,0350 | 0,5869 | 0,0357 | 0,5782 | 0,0324 | 0,5820 | 0,0335 | 0,5824 | 0,0313 | 0,5827 | 0,0296 | 0,5877 | 0,0294 | 0,5885 | 0,0301 | 0,5841 | 0,0303 |
| **L-fusiform** | **L-PAC** | **AD** | 1,2121 | 0,0411 | 1,2136 | 0,0395 | 1,2104 | 0,0457 | 1,1975 | 0,0312 | 1,2106 | 0,0447 | 1,2065 | 0,0456 | 1,2063 | 0,0370 | 1,2046 | 0,0308 | 1,2121 | 0,0353 | 1,2137 | 0,0364 |
| **L-VLD** | **L-PAC** | **MD** | 0,7787 | 0,0280 | 0,7957 | 0,0366 | 0,7926 | 0,0379 | 0,7862 | 0,0339 | 0,7962 | 0,0373 | 0,7901 | 0,0383 | 0,7949 | 0,0339 | 0,7906 | 0,0346 | 0,7897 | 0,0416 | 0,7887 | 0,0386 |
| **L-VLD** | **L-PAC** | **RD** | 0,5902 | 0,0296 | 0,6053 | 0,0273 | 0,5983 | 0,0385 | 0,5961 | 0,0300 | 0,6080 | 0,0299 | 0,5998 | 0,0339 | 0,6092 | 0,0259 | 0,5977 | 0,0335 | 0,5997 | 0,0395 | 0,5989 | 0,0355 |
| **L-VLD** | **L-PAC** | **AD** | 1,1558 | 0,0480 | 1,1766 | 0,0656 | 1,1813 | 0,0565 | 1,1663 | 0,0576 | 1,1728 | 0,0655 | 1,1707 | 0,0655 | 1,1664 | 0,0629 | 1,1764 | 0,0570 | 1,1698 | 0,0671 | 1,1682 | 0,0628 |
| **L-MGN** | **L-PAC** | **MD** | 0,7111 | 0,0229 | 0,6999 | 0,0226 | 0,6962 | 0,0222 | 0,7058 | 0,0259 | 0,7060 | 0,0234 | 0,7002 | 0,0168 | 0,7029 | 0,0207 | 0,7071 | 0,0196 | 0,7077 | 0,0256 | 0,7118 | 0,0225 |
| **L-MGN** | **L-PAC** | **RD** | 0,5300 | 0,0193 | 0,5190 | 0,0213 | 0,5171 | 0,0210 | 0,5225 | 0,0226 | 0,5247 | 0,0223 | 0,5197 | 0,0187 | 0,5222 | 0,0207 | 0,5258 | 0,0164 | 0,5261 | 0,0210 | 0,5294 | 0,0197 |
| **L-MGN** | **L-PAC** | **AD** | 1,0691 | 0,0352 | 1,0617 | 0,0285 | 1,0545 | 0,0328 | 1,0640 | 0,0286 | 1,0686 | 0,0319 | 1,0612 | 0,0209 | 1,0643 | 0,0256 | 1,0654 | 0,0254 | 1,0668 | 0,0353 | 1,0726 | 0,0292 |
| **L-Caudate** | **R-SOC-CN** | **MD** | 0,6902 | 0,0262 | 0,6900 | 0,0266 | 0,6858 | 0,0306 | 0,6892 | 0,0204 | 0,6863 | 0,0275 | 0,6863 | 0,0244 | 0,6866 | 0,0281 | 0,6854 | 0,0275 | 0,6859 | 0,0296 | 0,6825 | 0,0262 |
| **L-Caudate** | **R-SOC-CN** | **RD** | 0,5009 | 0,0220 | 0,5008 | 0,0184 | 0,4957 | 0,0235 | 0,5022 | 0,0203 | 0,4982 | 0,0169 | 0,4964 | 0,0189 | 0,4994 | 0,0190 | 0,5007 | 0,0219 | 0,4992 | 0,0229 | 0,5000 | 0,0232 |
| **L-Caudate** | **R-SOC-CN** | **AD** | 1,0688 | 0,0706 | 1,0686 | 0,0735 | 1,0661 | 0,0776 | 1,0632 | 0,0395 | 1,0625 | 0,0798 | 1,0662 | 0,0713 | 1,0609 | 0,0813 | 1,0549 | 0,0675 | 1,0592 | 0,0787 | 1,0475 | 0,0575 |
| **R-rMFC** | **R-SOC-CN** | **MD** | 0,7355 | 0,0242 | 0,7368 | 0,0235 | 0,7352 | 0,0266 | 0,7324 | 0,0190 | 0,7378 | 0,0277 | 0,7373 | 0,0245 | 0,7351 | 0,0232 | 0,7337 | 0,0205 | 0,7376 | 0,0253 | 0,7366 | 0,0224 |
| **R-rMFC** | **R-SOC-CN** | **RD** | 0,5624 | 0,0222 | 0,5624 | 0,0236 | 0,5633 | 0,0250 | 0,5576 | 0,0215 | 0,5638 | 0,0286 | 0,5633 | 0,0226 | 0,5624 | 0,0209 | 0,5625 | 0,0207 | 0,5651 | 0,0263 | 0,5652 | 0,0211 |
| **R-rMFC** | **R-SOC-CN** | **AD** | 1,0817 | 0,0337 | 1,0857 | 0,0306 | 1,0789 | 0,0374 | 1,0820 | 0,0248 | 1,0858 | 0,0333 | 1,0851 | 0,0328 | 1,0805 | 0,0329 | 1,0761 | 0,0283 | 1,0826 | 0,0345 | 1,0794 | 0,0288 |
| **L-SOC-CN** | **R-SOC-CN** | **MD** | 0,7406 | 0,0213 | 0,7423 | 0,0233 | 0,7372 | 0,0247 | 0,7429 | 0,0192 | 0,7481 | 0,0198 | 0,7348 | 0,0203 | 0,7438 | 0,0198 | 0,7411 | 0,0197 | 0,7432 | 0,0213 | 0,7441 | 0,0252 |
| **L-SOC-CN** | **R-SOC-CN** | **RD** | 0,5489 | 0,0220 | 0,5493 | 0,0249 | 0,5457 | 0,0270 | 0,5494 | 0,0220 | 0,5563 | 0,0240 | 0,5420 | 0,0216 | 0,5514 | 0,0226 | 0,5507 | 0,0221 | 0,5489 | 0,0254 | 0,5515 | 0,0266 |
| **L-SOC-CN** | **R-SOC-CN** | **AD** | 1,1239 | 0,0317 | 1,1284 | 0,0304 | 1,1168 | 0,0338 | 1,1298 | 0,0247 | 1,1318 | 0,0263 | 1,1204 | 0,0304 | 1,1285 | 0,0261 | 1,1218 | 0,0268 | 1,1281 | 0,0289 | 1,1254 | 0,0322 |
| **R-Pallidum** | **R-SOC-CN** | **MD** | 0,6987 | 0,0176 | 0,6951 | 0,0214 | 0,6942 | 0,0208 | 0,6984 | 0,0186 | 0,7000 | 0,0176 | 0,6976 | 0,0167 | 0,6979 | 0,0169 | 0,6914 | 0,0183 | 0,6996 | 0,0198 | 0,7005 | 0,0209 |
| **R-Pallidum** | **R-SOC-CN** | **RD** | 0,5147 | 0,0146 | 0,5118 | 0,0193 | 0,5129 | 0,0173 | 0,5144 | 0,0169 | 0,5161 | 0,0151 | 0,5146 | 0,0148 | 0,5137 | 0,0155 | 0,5088 | 0,0155 | 0,5155 | 0,0178 | 0,5166 | 0,0182 |
| **R-Pallidum** | **R-SOC-CN** | **AD** | 1,0669 | 0,0284 | 1,0616 | 0,0333 | 1,0567 | 0,0340 | 1,0663 | 0,0257 | 1,0679 | 0,0283 | 1,0635 | 0,0260 | 1,0663 | 0,0258 | 1,0566 | 0,0310 | 1,0677 | 0,0303 | 1,0684 | 0,0297 |
| **R-Amygdala** | **R-SOC-CN** | **MD** | 0,7264 | 0,0276 | 0,7188 | 0,0338 | 0,7231 | 0,0269 | 0,7271 | 0,0283 | 0,7317 | 0,0190 | 0,7217 | 0,0290 | 0,7266 | 0,0289 | 0,7166 | 0,0326 | 0,7228 | 0,0327 | 0,7225 | 0,0324 |
| **R-Amygdala** | **R-SOC-CN** | **RD** | 0,5398 | 0,0319 | 0,5315 | 0,0385 | 0,5409 | 0,0307 | 0,5433 | 0,0313 | 0,5462 | 0,0217 | 0,5333 | 0,0333 | 0,5402 | 0,0302 | 0,5341 | 0,0387 | 0,5364 | 0,0372 | 0,5361 | 0,0372 |
| **R-Amygdala** | **R-SOC-CN** | **AD** | 1,0994 | 0,0319 | 1,0935 | 0,0364 | 1,0873 | 0,0308 | 1,0946 | 0,0355 | 1,1026 | 0,0303 | 1,0984 | 0,0355 | 1,0994 | 0,0397 | 1,0816 | 0,0295 | 1,0957 | 0,0349 | 1,0952 | 0,0336 |
| **R-SPC** | **R-SOC-CN** | **MD** | 0,7393 | 0,0178 | 0,7338 | 0,0243 | 0,7395 | 0,0232 | 0,7333 | 0,0230 | 0,7385 | 0,0224 | 0,7353 | 0,0225 | 0,7355 | 0,0238 | 0,7366 | 0,0218 | 0,7405 | 0,0202 | 0,7402 | 0,0206 |
| **R-SPC** | **R-SOC-CN** | **RD** | 0,5436 | 0,0178 | 0,5379 | 0,0252 | 0,5444 | 0,0238 | 0,5384 | 0,0220 | 0,5425 | 0,0205 | 0,5389 | 0,0228 | 0,5399 | 0,0223 | 0,5410 | 0,0236 | 0,5435 | 0,0205 | 0,5423 | 0,0230 |
| **R-SPC** | **R-SOC-CN** | **AD** | 1,1307 | 0,0252 | 1,1256 | 0,0299 | 1,1297 | 0,0307 | 1,1232 | 0,0290 | 1,1305 | 0,0321 | 1,1283 | 0,0294 | 1,1267 | 0,0306 | 1,1278 | 0,0248 | 1,1346 | 0,0285 | 1,1360 | 0,0244 |
| **R-Anterior** | **R-SOC-CN** | **MD** | 0,7328 | 0,0593 | 0,7221 | 0,0302 | 0,7158 | 0,0400 | 0,7236 | 0,0413 | 0,7405 | 0,0581 | 0,7391 | 0,0606 | 0,7299 | 0,0373 | 0,7169 | 0,0321 | 0,7324 | 0,0646 | 0,7253 | 0,0455 |
| **R-Anterior** | **R-SOC-CN** | **RD** | 0,5588 | 0,0675 | 0,5448 | 0,0344 | 0,5387 | 0,0516 | 0,5500 | 0,0436 | 0,5641 | 0,0695 | 0,5641 | 0,0689 | 0,5555 | 0,0408 | 0,5473 | 0,0371 | 0,5579 | 0,0791 | 0,5493 | 0,0600 |
| **R-Anterior** | **R-SOC-CN** | **AD** | 1,0809 | 0,0578 | 1,0767 | 0,0500 | 1,0701 | 0,0523 | 1,0707 | 0,0518 | 1,0934 | 0,0561 | 1,0892 | 0,0537 | 1,0788 | 0,0435 | 1,0562 | 0,0404 | 1,0814 | 0,0605 | 1,0774 | 0,0485 |
| **R-Putamen** | **R-SOC-CN** | **MD** | 0,7034 | 0,0169 | 0,6995 | 0,0218 | 0,6981 | 0,0208 | 0,7037 | 0,0180 | 0,7056 | 0,0153 | 0,7024 | 0,0166 | 0,7020 | 0,0144 | 0,6957 | 0,0177 | 0,7026 | 0,0197 | 0,7051 | 0,0196 |
| **R-Putamen** | **R-SOC-CN** | **RD** | 0,5122 | 0,0166 | 0,5081 | 0,0212 | 0,5090 | 0,0192 | 0,5132 | 0,0167 | 0,5135 | 0,0163 | 0,5112 | 0,0176 | 0,5102 | 0,0160 | 0,5053 | 0,0171 | 0,5110 | 0,0194 | 0,5142 | 0,0171 |
| **R-Putamen** | **R-SOC-CN** | **AD** | 1,0859 | 0,0288 | 1,0824 | 0,0333 | 1,0761 | 0,0340 | 1,0848 | 0,0245 | 1,0899 | 0,0261 | 1,0847 | 0,0265 | 1,0856 | 0,0251 | 1,0765 | 0,0309 | 1,0860 | 0,0311 | 1,0871 | 0,0290 |
| **L-MGN** | **R-SOC-CN** | **MD** | 0,7137 | 0,0287 | 0,7085 | 0,0289 | 0,7039 | 0,0273 | 0,7173 | 0,0243 | 0,7147 | 0,0229 | 0,7088 | 0,0229 | 0,7126 | 0,0208 | 0,7107 | 0,0241 | 0,7123 | 0,0275 | 0,7197 | 0,0257 |
| **L-MGN** | **R-SOC-CN** | **RD** | 0,5348 | 0,0319 | 0,5340 | 0,0301 | 0,5319 | 0,0299 | 0,5339 | 0,0240 | 0,5382 | 0,0276 | 0,5318 | 0,0282 | 0,5367 | 0,0255 | 0,5315 | 0,0249 | 0,5347 | 0,0297 | 0,5428 | 0,0261 |
| **L-MGN** | **R-SOC-CN** | **AD** | 1,0778 | 0,0422 | 1,0638 | 0,0413 | 1,0541 | 0,0418 | 1,0841 | 0,0389 | 1,0734 | 0,0351 | 1,0691 | 0,0344 | 1,0705 | 0,0296 | 1,0691 | 0,0376 | 1,0736 | 0,0461 | 1,0790 | 0,0403 |
| **L-IC** | **R-SOC-CN** | **MD** | 0,7453 | 0,0164 | 0,7491 | 0,0248 | 0,7455 | 0,0256 | 0,7482 | 0,0195 | 0,7465 | 0,0253 | 0,7464 | 0,0212 | 0,7418 | 0,0247 | 0,7488 | 0,0206 | 0,7479 | 0,0224 | 0,7497 | 0,0253 |
| **L-IC** | **R-SOC-CN** | **RD** | 0,5502 | 0,0175 | 0,5535 | 0,0210 | 0,5523 | 0,0218 | 0,5517 | 0,0225 | 0,5513 | 0,0212 | 0,5515 | 0,0167 | 0,5484 | 0,0194 | 0,5519 | 0,0176 | 0,5505 | 0,0225 | 0,5529 | 0,0236 |
| **L-IC** | **R-SOC-CN** | **AD** | 1,1402 | 0,0209 | 1,1407 | 0,0264 | 1,1356 | 0,0266 | 1,1414 | 0,0240 | 1,1358 | 0,0293 | 1,1362 | 0,0294 | 1,1311 | 0,0330 | 1,1420 | 0,0247 | 1,1386 | 0,0279 | 1,1342 | 0,0284 |
| **R-Caudate** | **R-SOC-CN** | **MD** | 0,7107 | 0,0199 | 0,7058 | 0,0242 | 0,7049 | 0,0218 | 0,7062 | 0,0206 | 0,7080 | 0,0217 | 0,7082 | 0,0222 | 0,7077 | 0,0231 | 0,7024 | 0,0188 | 0,7077 | 0,0173 | 0,7096 | 0,0219 |
| **R-Caudate** | **R-SOC-CN** | **RD** | 0,5300 | 0,0205 | 0,5283 | 0,0217 | 0,5315 | 0,0201 | 0,5274 | 0,0226 | 0,5289 | 0,0212 | 0,5308 | 0,0208 | 0,5301 | 0,0194 | 0,5245 | 0,0199 | 0,5282 | 0,0193 | 0,5294 | 0,0229 |
| **R-Caudate** | **R-SOC-CN** | **AD** | 1,0722 | 0,0304 | 1,0607 | 0,0389 | 1,0518 | 0,0340 | 1,0639 | 0,0325 | 1,0663 | 0,0444 | 1,0631 | 0,0333 | 1,0627 | 0,0451 | 1,0582 | 0,0347 | 1,0667 | 0,0348 | 1,0699 | 0,0354 |
| **R-Medio_Dorsal** | **R-SOC-CN** | **MD** | 0,7153 | 0,0350 | 0,7185 | 0,0330 | 0,7163 | 0,0419 | 0,7206 | 0,0383 | 0,7279 | 0,0356 | 0,7258 | 0,0328 | 0,7220 | 0,0358 | 0,7204 | 0,0394 | 0,7281 | 0,0441 | 0,7290 | 0,0442 |
| **R-Medio_Dorsal** | **R-SOC-CN** | **RD** | 0,5473 | 0,0491 | 0,5487 | 0,0484 | 0,5453 | 0,0387 | 0,5515 | 0,0473 | 0,5471 | 0,0440 | 0,5623 | 0,0548 | 0,5608 | 0,0571 | 0,5498 | 0,0421 | 0,5624 | 0,0553 | 0,5674 | 0,0615 |
| **R-Medio_Dorsal** | **R-SOC-CN** | **AD** | 1,0769 | 0,0754 | 1,0820 | 0,0683 | 1,0583 | 0,0672 | 1,0791 | 0,0694 | 1,0895 | 0,0442 | 1,0974 | 0,0679 | 1,0900 | 0,0724 | 1,0616 | 0,0527 | 1,0835 | 0,0780 | 1,0959 | 0,0775 |
| **R-SFC** | **R-SOC-CN** | **MD** | 0,7180 | 0,0198 | 0,7171 | 0,0212 | 0,7162 | 0,0216 | 0,7147 | 0,0191 | 0,7205 | 0,0197 | 0,7171 | 0,0187 | 0,7186 | 0,0178 | 0,7147 | 0,0182 | 0,7199 | 0,0207 | 0,7181 | 0,0207 |
| **R-SFC** | **R-SOC-CN** | **RD** | 0,5336 | 0,0177 | 0,5350 | 0,0192 | 0,5339 | 0,0200 | 0,5323 | 0,0178 | 0,5362 | 0,0192 | 0,5333 | 0,0169 | 0,5349 | 0,0169 | 0,5330 | 0,0165 | 0,5348 | 0,0214 | 0,5346 | 0,0191 |
| **R-SFC** | **R-SOC-CN** | **AD** | 1,0868 | 0,0300 | 1,0814 | 0,0310 | 1,0809 | 0,0306 | 1,0795 | 0,0281 | 1,0890 | 0,0277 | 1,0847 | 0,0269 | 1,0860 | 0,0266 | 1,0782 | 0,0301 | 1,0900 | 0,0264 | 1,0850 | 0,0298 |
| **L-Putamen** | **R-SOC-CN** | **MD** | 0,6785 | 0,0245 | 0,6741 | 0,0273 | 0,6748 | 0,0277 | 0,6735 | 0,0208 | 0,6772 | 0,0231 | 0,6796 | 0,0206 | 0,6768 | 0,0213 | 0,6718 | 0,0244 | 0,6843 | 0,0214 | 0,6784 | 0,0270 |
| **L-Putamen** | **R-SOC-CN** | **RD** | 0,4836 | 0,0235 | 0,4798 | 0,0281 | 0,4847 | 0,0276 | 0,4784 | 0,0207 | 0,4826 | 0,0244 | 0,4856 | 0,0234 | 0,4831 | 0,0264 | 0,4784 | 0,0241 | 0,4896 | 0,0235 | 0,4821 | 0,0252 |
| **L-Putamen** | **R-SOC-CN** | **AD** | 1,0684 | 0,0412 | 1,0628 | 0,0425 | 1,0551 | 0,0436 | 1,0638 | 0,0340 | 1,0665 | 0,0361 | 1,0676 | 0,0347 | 1,0642 | 0,0322 | 1,0586 | 0,0424 | 1,0736 | 0,0379 | 1,0710 | 0,0444 |
| **L-Pallidum** | **R-SOC-CN** | **MD** | 0,6809 | 0,0242 | 0,6751 | 0,0255 | 0,6755 | 0,0261 | 0,6763 | 0,0199 | 0,6783 | 0,0221 | 0,6789 | 0,0185 | 0,6756 | 0,0182 | 0,6732 | 0,0231 | 0,6823 | 0,0211 | 0,6796 | 0,0260 |
| **L-Pallidum** | **R-SOC-CN** | **RD** | 0,4904 | 0,0201 | 0,4864 | 0,0258 | 0,4902 | 0,0225 | 0,4850 | 0,0209 | 0,4868 | 0,0237 | 0,4904 | 0,0184 | 0,4851 | 0,0228 | 0,4843 | 0,0199 | 0,4911 | 0,0196 | 0,4865 | 0,0238 |
| **L-Pallidum** | **R-SOC-CN** | **AD** | 1,0619 | 0,0423 | 1,0524 | 0,0405 | 1,0462 | 0,0429 | 1,0590 | 0,0336 | 1,0613 | 0,0352 | 1,0558 | 0,0301 | 1,0567 | 0,0283 | 1,0510 | 0,0431 | 1,0646 | 0,0373 | 1,0660 | 0,0429 |
| **L-SFC** | **R-SOC-CN** | **MD** | 0,7115 | 0,0208 | 0,7092 | 0,0259 | 0,7120 | 0,0238 | 0,7100 | 0,0197 | 0,7113 | 0,0237 | 0,7157 | 0,0210 | 0,7110 | 0,0200 | 0,7072 | 0,0202 | 0,7163 | 0,0201 | 0,7127 | 0,0258 |
| **L-SFC** | **R-SOC-CN** | **RD** | 0,5100 | 0,0205 | 0,5081 | 0,0275 | 0,5141 | 0,0230 | 0,5102 | 0,0246 | 0,5110 | 0,0265 | 0,5166 | 0,0198 | 0,5124 | 0,0252 | 0,5089 | 0,0222 | 0,5156 | 0,0204 | 0,5108 | 0,0259 |
| **L-SFC** | **R-SOC-CN** | **AD** | 1,1146 | 0,0314 | 1,1114 | 0,0370 | 1,1078 | 0,0377 | 1,1096 | 0,0285 | 1,1119 | 0,0337 | 1,1139 | 0,0321 | 1,1082 | 0,0271 | 1,1037 | 0,0303 | 1,1176 | 0,0309 | 1,1165 | 0,0377 |
| **R-Medio_Dorsal** | **R-MGN** | **MD** | 0,7276 | 0,0304 | 0,7221 | 0,0360 | 0,7197 | 0,0338 | 0,7315 | 0,0421 | 0,7194 | 0,0324 | 0,7249 | 0,0325 | 0,7272 | 0,0357 | 0,7194 | 0,0322 | 0,7172 | 0,0327 | 0,7238 | 0,0387 |
| **R-Medio_Dorsal** | **R-MGN** | **RD** | 0,5802 | 0,0444 | 0,5692 | 0,0421 | 0,5801 | 0,0377 | 0,5787 | 0,0469 | 0,5749 | 0,0377 | 0,5763 | 0,0431 | 0,5754 | 0,0351 | 0,5779 | 0,0379 | 0,5753 | 0,0409 | 0,5836 | 0,0435 |
| **R-Medio_Dorsal** | **R-MGN** | **AD** | 1,0224 | 0,0869 | 1,0279 | 0,0869 | 0,9990 | 0,0869 | 1,0371 | 0,0783 | 1,0085 | 0,1007 | 1,0221 | 0,0700 | 1,0308 | 0,0879 | 1,0022 | 0,0872 | 1,0010 | 0,0891 | 1,0042 | 0,0945 |
| **R-Anterior** | **R-MGN** | **MD** | 0,7484 | 0,0333 | 0,7441 | 0,0345 | 0,7472 | 0,0375 | 0,7507 | 0,0344 | 0,7506 | 0,0304 | 0,7484 | 0,0380 | 0,7498 | 0,0310 | 0,7420 | 0,0299 | 0,7451 | 0,0349 | 0,7484 | 0,0401 |
| **R-Anterior** | **R-MGN** | **RD** | 0,5934 | 0,0389 | 0,5899 | 0,0422 | 0,5970 | 0,0372 | 0,5958 | 0,0393 | 0,5976 | 0,0324 | 0,5938 | 0,0435 | 0,5974 | 0,0274 | 0,5934 | 0,0309 | 0,5908 | 0,0338 | 0,5963 | 0,0401 |
| **R-Anterior** | **R-MGN** | **AD** | 1,0585 | 0,0687 | 1,0525 | 0,0648 | 1,0477 | 0,0770 | 1,0606 | 0,0629 | 1,0566 | 0,0747 | 1,0576 | 0,0620 | 1,0547 | 0,0762 | 1,0393 | 0,0696 | 1,0537 | 0,0724 | 1,0527 | 0,0748 |
| **R-precuneus** | **R-MGN** | **MD** | 0,7492 | 0,0360 | 0,7565 | 0,0285 | 0,7584 | 0,0308 | 0,7486 | 0,0331 | 0,7629 | 0,0292 | 0,7563 | 0,0272 | 0,7581 | 0,0237 | 0,7534 | 0,0324 | 0,7604 | 0,0270 | 0,7526 | 0,0348 |
| **R-precuneus** | **R-MGN** | **RD** | 0,5620 | 0,0297 | 0,5719 | 0,0289 | 0,5752 | 0,0302 | 0,5676 | 0,0264 | 0,5747 | 0,0330 | 0,5697 | 0,0307 | 0,5746 | 0,0243 | 0,5725 | 0,0239 | 0,5719 | 0,0306 | 0,5696 | 0,0264 |
| **R-precuneus** | **R-MGN** | **AD** | 1,1237 | 0,0702 | 1,1258 | 0,0379 | 1,1248 | 0,0403 | 1,1108 | 0,0653 | 1,1393 | 0,0379 | 1,1296 | 0,0383 | 1,1251 | 0,0317 | 1,1151 | 0,0646 | 1,1376 | 0,0366 | 1,1187 | 0,0659 |
| **R-entorhinal** | **R-MGN** | **MD** | 0,8008 | 0,0366 | 0,7872 | 0,0334 | 0,7926 | 0,0398 | 0,7907 | 0,0254 | 0,8013 | 0,0325 | 0,7864 | 0,0389 | 0,7966 | 0,0327 | 0,7946 | 0,0356 | 0,7977 | 0,0397 | 0,7970 | 0,0374 |
| **R-entorhinal** | **R-MGN** | **RD** | 0,6369 | 0,0397 | 0,6240 | 0,0307 | 0,6325 | 0,0358 | 0,6289 | 0,0276 | 0,6312 | 0,0344 | 0,6261 | 0,0362 | 0,6306 | 0,0334 | 0,6347 | 0,0312 | 0,6375 | 0,0376 | 0,6361 | 0,0345 |
| **R-entorhinal** | **R-MGN** | **AD** | 1,1287 | 0,0527 | 1,1136 | 0,0548 | 1,1129 | 0,0596 | 1,1141 | 0,0412 | 1,1414 | 0,0465 | 1,1069 | 0,0593 | 1,1285 | 0,0519 | 1,1143 | 0,0583 | 1,1180 | 0,0585 | 1,1186 | 0,0613 |
| **R-SOC-CN** | **R-MGN** | **MD** | 0,7360 | 0,0152 | 0,7383 | 0,0232 | 0,7342 | 0,0234 | 0,7342 | 0,0220 | 0,7365 | 0,0209 | 0,7365 | 0,0192 | 0,7335 | 0,0227 | 0,7365 | 0,0198 | 0,7364 | 0,0194 | 0,7417 | 0,0185 |
| **R-SOC-CN** | **R-MGN** | **RD** | 0,5357 | 0,0152 | 0,5390 | 0,0211 | 0,5374 | 0,0212 | 0,5355 | 0,0219 | 0,5395 | 0,0205 | 0,5380 | 0,0181 | 0,5384 | 0,0211 | 0,5361 | 0,0187 | 0,5369 | 0,0201 | 0,5413 | 0,0217 |
| **R-SOC-CN** | **R-MGN** | **AD** | 1,1368 | 0,0246 | 1,1369 | 0,0370 | 1,1279 | 0,0355 | 1,1315 | 0,0322 | 1,1304 | 0,0328 | 1,1336 | 0,0323 | 1,1239 | 0,0344 | 1,1375 | 0,0315 | 1,1353 | 0,0267 | 1,1425 | 0,0245 |
| **R-Putamen** | **R-MGN** | **MD** | 0,7218 | 0,0220 | 0,7227 | 0,0222 | 0,7201 | 0,0238 | 0,7227 | 0,0210 | 0,7274 | 0,0217 | 0,7203 | 0,0208 | 0,7279 | 0,0207 | 0,7227 | 0,0203 | 0,7237 | 0,0246 | 0,7247 | 0,0229 |
| **R-Putamen** | **R-MGN** | **RD** | 0,5282 | 0,0210 | 0,5272 | 0,0210 | 0,5268 | 0,0217 | 0,5285 | 0,0241 | 0,5320 | 0,0230 | 0,5279 | 0,0219 | 0,5355 | 0,0203 | 0,5288 | 0,0210 | 0,5308 | 0,0229 | 0,5310 | 0,0230 |
| **R-Putamen** | **R-MGN** | **AD** | 1,1092 | 0,0408 | 1,1137 | 0,0351 | 1,1066 | 0,0419 | 1,1110 | 0,0275 | 1,1181 | 0,0374 | 1,1051 | 0,0356 | 1,1127 | 0,0371 | 1,1107 | 0,0299 | 1,1095 | 0,0441 | 1,1123 | 0,0381 |
| **R-IPC** | **R-MGN** | **MD** | 0,7696 | 0,0216 | 0,7682 | 0,0291 | 0,7722 | 0,0272 | 0,7649 | 0,0271 | 0,7752 | 0,0228 | 0,7677 | 0,0256 | 0,7743 | 0,0211 | 0,7702 | 0,0236 | 0,7737 | 0,0232 | 0,7740 | 0,0234 |
| **R-IPC** | **R-MGN** | **RD** | 0,5783 | 0,0232 | 0,5780 | 0,0344 | 0,5817 | 0,0316 | 0,5745 | 0,0349 | 0,5842 | 0,0253 | 0,5761 | 0,0320 | 0,5847 | 0,0240 | 0,5809 | 0,0261 | 0,5813 | 0,0278 | 0,5826 | 0,0272 |
| **R-IPC** | **R-MGN** | **AD** | 1,1523 | 0,0263 | 1,1486 | 0,0339 | 1,1532 | 0,0318 | 1,1456 | 0,0208 | 1,1571 | 0,0304 | 1,1509 | 0,0278 | 1,1536 | 0,0295 | 1,1490 | 0,0311 | 1,1584 | 0,0264 | 1,1570 | 0,0273 |
| **R-Amygdala** | **R-MGN** | **MD** | 0,7923 | 0,0310 | 0,7882 | 0,0322 | 0,7908 | 0,0332 | 0,7970 | 0,0256 | 0,7956 | 0,0333 | 0,7872 | 0,0320 | 0,7927 | 0,0292 | 0,7882 | 0,0282 | 0,7958 | 0,0341 | 0,7982 | 0,0311 |
| **R-Amygdala** | **R-MGN** | **RD** | 0,6196 | 0,0396 | 0,6130 | 0,0361 | 0,6212 | 0,0359 | 0,6244 | 0,0306 | 0,6200 | 0,0413 | 0,6169 | 0,0374 | 0,6175 | 0,0357 | 0,6152 | 0,0364 | 0,6269 | 0,0401 | 0,6259 | 0,0355 |
| **R-Amygdala** | **R-MGN** | **AD** | 1,1377 | 0,0418 | 1,1386 | 0,0356 | 1,1301 | 0,0439 | 1,1424 | 0,0352 | 1,1467 | 0,0336 | 1,1277 | 0,0415 | 1,1431 | 0,0334 | 1,1342 | 0,0392 | 1,1338 | 0,0426 | 1,1427 | 0,0416 |
| **R-Pallidum** | **R-MGN** | **MD** | 0,7008 | 0,0195 | 0,7032 | 0,0234 | 0,6992 | 0,0239 | 0,7014 | 0,0215 | 0,7052 | 0,0203 | 0,6993 | 0,0186 | 0,7042 | 0,0202 | 0,7020 | 0,0175 | 0,7023 | 0,0226 | 0,7030 | 0,0218 |
| **R-Pallidum** | **R-MGN** | **RD** | 0,5071 | 0,0201 | 0,5098 | 0,0232 | 0,5081 | 0,0217 | 0,5078 | 0,0255 | 0,5127 | 0,0234 | 0,5063 | 0,0201 | 0,5107 | 0,0235 | 0,5100 | 0,0212 | 0,5102 | 0,0235 | 0,5100 | 0,0235 |
| **R-Pallidum** | **R-MGN** | **AD** | 1,0883 | 0,0340 | 1,0900 | 0,0329 | 1,0815 | 0,0371 | 1,0888 | 0,0297 | 1,0901 | 0,0354 | 1,0854 | 0,0275 | 1,0912 | 0,0338 | 1,0861 | 0,0287 | 1,0866 | 0,0390 | 1,0889 | 0,0345 |
| **L-SOC-CN** | **R-MGN** | **MD** | 0,7129 | 0,0173 | 0,7050 | 0,0153 | 0,7040 | 0,0166 | 0,7096 | 0,0187 | 0,7121 | 0,0163 | 0,7093 | 0,0144 | 0,7104 | 0,0177 | 0,7092 | 0,0182 | 0,7132 | 0,0207 | 0,7159 | 0,0205 |
| **L-SOC-CN** | **R-MGN** | **RD** | 0,5292 | 0,0189 | 0,5212 | 0,0155 | 0,5231 | 0,0161 | 0,5231 | 0,0143 | 0,5289 | 0,0194 | 0,5245 | 0,0195 | 0,5275 | 0,0163 | 0,5252 | 0,0167 | 0,5309 | 0,0198 | 0,5320 | 0,0190 |
| **L-SOC-CN** | **R-MGN** | **AD** | 1,0794 | 0,0285 | 1,0751 | 0,0268 | 1,0732 | 0,0297 | 1,0761 | 0,0288 | 1,0845 | 0,0248 | 1,0782 | 0,0214 | 1,0791 | 0,0262 | 1,0737 | 0,0252 | 1,0853 | 0,0287 | 1,0853 | 0,0241 |
| **R-fusiform** | **R-MGN** | **MD** | 0,7922 | 0,0363 | 0,7915 | 0,0301 | 0,8009 | 0,0495 | 0,7892 | 0,0303 | 0,7965 | 0,0351 | 0,7948 | 0,0389 | 0,8012 | 0,0367 | 0,7923 | 0,0300 | 0,8076 | 0,0496 | 0,8058 | 0,0456 |
| **R-fusiform** | **R-MGN** | **RD** | 0,6050 | 0,0408 | 0,6048 | 0,0361 | 0,6150 | 0,0472 | 0,6056 | 0,0421 | 0,6137 | 0,0385 | 0,6096 | 0,0453 | 0,6199 | 0,0411 | 0,6088 | 0,0373 | 0,6227 | 0,0480 | 0,6181 | 0,0462 |
| **R-fusiform** | **R-MGN** | **AD** | 1,1665 | 0,0407 | 1,1650 | 0,0337 | 1,1727 | 0,0648 | 1,1563 | 0,0308 | 1,1619 | 0,0354 | 1,1653 | 0,0414 | 1,1639 | 0,0394 | 1,1591 | 0,0302 | 1,1775 | 0,0625 | 1,1811 | 0,0577 |
| **R-rMFC** | **R-MGN** | **MD** | 0,7489 | 0,0250 | 0,7536 | 0,0275 | 0,7535 | 0,0291 | 0,7502 | 0,0215 | 0,7581 | 0,0304 | 0,7519 | 0,0297 | 0,7530 | 0,0232 | 0,7481 | 0,0215 | 0,7530 | 0,0321 | 0,7526 | 0,0306 |
| **R-rMFC** | **R-MGN** | **RD** | 0,5725 | 0,0245 | 0,5746 | 0,0305 | 0,5783 | 0,0268 | 0,5728 | 0,0253 | 0,5836 | 0,0326 | 0,5743 | 0,0310 | 0,5756 | 0,0259 | 0,5708 | 0,0234 | 0,5791 | 0,0314 | 0,5800 | 0,0296 |
| **R-rMFC** | **R-MGN** | **AD** | 1,1017 | 0,0339 | 1,1070 | 0,0341 | 1,1038 | 0,0438 | 1,1048 | 0,0314 | 1,1072 | 0,0371 | 1,1072 | 0,0354 | 1,1031 | 0,0353 | 1,0978 | 0,0328 | 1,0958 | 0,0440 | 1,0977 | 0,0391 |
| **R-ITC** | **R-MGN** | **MD** | 0,7857 | 0,0306 | 0,7764 | 0,0261 | 0,7870 | 0,0315 | 0,7807 | 0,0304 | 0,7830 | 0,0334 | 0,7782 | 0,0327 | 0,7905 | 0,0333 | 0,7789 | 0,0270 | 0,7895 | 0,0351 | 0,7873 | 0,0337 |
| **R-ITC** | **R-MGN** | **RD** | 0,5970 | 0,0394 | 0,5867 | 0,0311 | 0,6042 | 0,0387 | 0,5908 | 0,0403 | 0,5964 | 0,0428 | 0,5935 | 0,0396 | 0,6061 | 0,0416 | 0,5878 | 0,0341 | 0,6046 | 0,0435 | 0,5991 | 0,0402 |
| **R-ITC** | **R-MGN** | **AD** | 1,1632 | 0,0277 | 1,1560 | 0,0286 | 1,1528 | 0,0270 | 1,1603 | 0,0260 | 1,1563 | 0,0279 | 1,1476 | 0,0351 | 1,1595 | 0,0358 | 1,1609 | 0,0288 | 1,1593 | 0,0317 | 1,1637 | 0,0358 |
| **R-SPC** | **R-MGN** | **MD** | 0,7560 | 0,0204 | 0,7527 | 0,0245 | 0,7566 | 0,0248 | 0,7488 | 0,0193 | 0,7573 | 0,0248 | 0,7526 | 0,0234 | 0,7552 | 0,0211 | 0,7554 | 0,0195 | 0,7570 | 0,0234 | 0,7569 | 0,0208 |
| **R-SPC** | **R-MGN** | **RD** | 0,5683 | 0,0197 | 0,5671 | 0,0251 | 0,5701 | 0,0263 | 0,5645 | 0,0205 | 0,5700 | 0,0243 | 0,5655 | 0,0226 | 0,5694 | 0,0215 | 0,5704 | 0,0204 | 0,5691 | 0,0233 | 0,5689 | 0,0230 |
| **R-SPC** | **R-MGN** | **AD** | 1,1313 | 0,0281 | 1,1240 | 0,0306 | 1,1297 | 0,0297 | 1,1174 | 0,0211 | 1,1319 | 0,0317 | 1,1268 | 0,0296 | 1,1268 | 0,0270 | 1,1254 | 0,0248 | 1,1328 | 0,0315 | 1,1329 | 0,0234 |
| **R-SFC** | **R-MGN** | **MD** | 0,7204 | 0,0209 | 0,7234 | 0,0234 | 0,7209 | 0,0247 | 0,7190 | 0,0173 | 0,7222 | 0,0255 | 0,7199 | 0,0241 | 0,7246 | 0,0223 | 0,7217 | 0,0187 | 0,7265 | 0,0247 | 0,7240 | 0,0228 |
| **R-SFC** | **R-MGN** | **RD** | 0,5344 | 0,0218 | 0,5405 | 0,0243 | 0,5362 | 0,0249 | 0,5353 | 0,0190 | 0,5393 | 0,0242 | 0,5340 | 0,0232 | 0,5402 | 0,0251 | 0,5416 | 0,0216 | 0,5403 | 0,0270 | 0,5377 | 0,0218 |
| **R-SFC** | **R-MGN** | **AD** | 1,0923 | 0,0313 | 1,0892 | 0,0334 | 1,0902 | 0,0304 | 1,0864 | 0,0253 | 1,0880 | 0,0359 | 1,0916 | 0,0342 | 1,0933 | 0,0334 | 1,0820 | 0,0275 | 1,0990 | 0,0327 | 1,0965 | 0,0364 |
| **R-Ventral_Anterior** | **R-MGN** | **MD** | 0,7338 | 0,0455 | 0,7393 | 0,0645 | 0,7433 | 0,0724 | 0,7471 | 0,0624 | 0,7404 | 0,0609 | 0,7428 | 0,0697 | 0,7368 | 0,0411 | 0,7334 | 0,0325 | 0,7340 | 0,0415 | 0,7361 | 0,0425 |
| **R-Ventral_Anterior** | **R-MGN** | **RD** | 0,5973 | 0,0413 | 0,5952 | 0,0598 | 0,6042 | 0,0621 | 0,5954 | 0,0609 | 0,6052 | 0,0510 | 0,6071 | 0,0620 | 0,6003 | 0,0366 | 0,5926 | 0,0295 | 0,5988 | 0,0379 | 0,6013 | 0,0410 |
| **R-Ventral_Anterior** | **R-MGN** | **AD** | 1,0070 | 0,0947 | 1,0275 | 0,1012 | 1,0215 | 0,1136 | 1,0505 | 0,0966 | 1,0108 | 0,1023 | 1,0144 | 0,1100 | 1,0098 | 0,0812 | 1,0151 | 0,0743 | 1,0045 | 0,0811 | 1,0057 | 0,0833 |
| **R-Hippo_CA1** | **R-MGN** | **MD** | 0,7969 | 0,0647 | 0,7852 | 0,0447 | 0,7939 | 0,0553 | 0,7981 | 0,0497 | 0,8017 | 0,0604 | 0,7880 | 0,0565 | 0,7911 | 0,0490 | 0,7954 | 0,0614 | 0,7912 | 0,0527 | 0,7923 | 0,0633 |
| **R-Hippo_CA1** | **R-MGN** | **RD** | 0,6054 | 0,0694 | 0,6043 | 0,0795 | 0,6183 | 0,0809 | 0,6327 | 0,0939 | 0,6201 | 0,0898 | 0,6085 | 0,0895 | 0,5953 | 0,0620 | 0,6036 | 0,0692 | 0,6166 | 0,0819 | 0,5989 | 0,0731 |
| **R-Hippo_CA1** | **R-MGN** | **AD** | 1,1798 | 0,0653 | 1,1867 | 0,0831 | 1,1838 | 0,0935 | 1,2060 | 0,1113 | 1,2025 | 0,0832 | 1,1882 | 0,0874 | 1,1827 | 0,0453 | 1,1789 | 0,0537 | 1,1824 | 0,0919 | 1,1790 | 0,0527 |
| **R-Caudate** | **R-MGN** | **MD** | 0,7262 | 0,0295 | 0,7223 | 0,0310 | 0,7261 | 0,0379 | 0,7259 | 0,0242 | 0,7246 | 0,0272 | 0,7252 | 0,0350 | 0,7285 | 0,0289 | 0,7245 | 0,0211 | 0,7301 | 0,0325 | 0,7285 | 0,0330 |
| **R-Caudate** | **R-MGN** | **RD** | 0,5623 | 0,0291 | 0,5604 | 0,0261 | 0,5657 | 0,0337 | 0,5594 | 0,0250 | 0,5649 | 0,0210 | 0,5648 | 0,0298 | 0,5676 | 0,0262 | 0,5611 | 0,0218 | 0,5699 | 0,0298 | 0,5642 | 0,0312 |
| **R-Caudate** | **R-MGN** | **AD** | 1,0541 | 0,0507 | 1,0459 | 0,0552 | 1,0470 | 0,0562 | 1,0589 | 0,0457 | 1,0441 | 0,0554 | 1,0460 | 0,0570 | 1,0502 | 0,0521 | 1,0512 | 0,0419 | 1,0506 | 0,0535 | 1,0572 | 0,0549 |
| **L-IC** | **R-IC** | **MD** | 0,6816 | 0,0261 | 0,6721 | 0,0285 | 0,6781 | 0,0318 | 0,6805 | 0,0283 | 0,6798 | 0,0270 | 0,6821 | 0,0299 | 0,6784 | 0,0279 | 0,6758 | 0,0265 | 0,6871 | 0,0227 | 0,6835 | 0,0298 |
| **L-IC** | **R-IC** | **RD** | 0,4963 | 0,0188 | 0,4874 | 0,0270 | 0,4899 | 0,0271 | 0,4886 | 0,0227 | 0,4970 | 0,0235 | 0,4887 | 0,0254 | 0,4884 | 0,0243 | 0,4890 | 0,0212 | 0,4962 | 0,0190 | 0,4975 | 0,0189 |
| **L-IC** | **R-IC** | **AD** | 1,0622 | 0,0595 | 1,0637 | 0,0546 | 1,0639 | 0,0577 | 1,0707 | 0,0535 | 1,0618 | 0,0536 | 1,0686 | 0,0557 | 1,0587 | 0,0533 | 1,0555 | 0,0545 | 1,0717 | 0,0596 | 1,0729 | 0,0622 |
| **R-Putamen** | **R-IC** | **MD** | 0,6975 | 0,0232 | 0,6962 | 0,0232 | 0,6927 | 0,0251 | 0,7037 | 0,0233 | 0,6983 | 0,0206 | 0,6954 | 0,0227 | 0,6975 | 0,0217 | 0,6971 | 0,0228 | 0,6980 | 0,0207 | 0,7011 | 0,0235 |
| **R-Putamen** | **R-IC** | **RD** | 0,5155 | 0,0264 | 0,5115 | 0,0261 | 0,5140 | 0,0266 | 0,5214 | 0,0279 | 0,5133 | 0,0240 | 0,5132 | 0,0279 | 0,5161 | 0,0234 | 0,5138 | 0,0279 | 0,5138 | 0,0238 | 0,5174 | 0,0238 |
| **R-Putamen** | **R-IC** | **AD** | 1,0617 | 0,0440 | 1,0656 | 0,0353 | 1,0501 | 0,0482 | 1,0682 | 0,0300 | 1,0681 | 0,0351 | 1,0597 | 0,0415 | 1,0603 | 0,0359 | 1,0639 | 0,0319 | 1,0665 | 0,0406 | 1,0683 | 0,0398 |
| **R-Anterior** | **R-IC** | **MD** | 0,7157 | 0,0369 | 0,7002 | 0,0324 | 0,6959 | 0,0311 | 0,7053 | 0,0327 | 0,7078 | 0,0417 | 0,7150 | 0,0375 | 0,7016 | 0,0386 | 0,7006 | 0,0299 | 0,7108 | 0,0373 | 0,7115 | 0,0263 |
| **R-Anterior** | **R-IC** | **RD** | 0,5783 | 0,0383 | 0,5589 | 0,0336 | 0,5597 | 0,0272 | 0,5640 | 0,0341 | 0,5720 | 0,0448 | 0,5756 | 0,0440 | 0,5638 | 0,0369 | 0,5646 | 0,0272 | 0,5761 | 0,0386 | 0,5750 | 0,0282 |
| **R-Anterior** | **R-IC** | **AD** | 0,9906 | 0,0489 | 0,9829 | 0,0440 | 0,9684 | 0,0461 | 0,9880 | 0,0480 | 0,9793 | 0,0433 | 0,9938 | 0,0440 | 0,9771 | 0,0478 | 0,9727 | 0,0461 | 0,9803 | 0,0459 | 0,9844 | 0,0345 |
| **L-SOC-CN** | **R-IC** | **MD** | 0,7601 | 0,0282 | 0,7518 | 0,0305 | 0,7458 | 0,0294 | 0,7526 | 0,0288 | 0,7519 | 0,0252 | 0,7562 | 0,0244 | 0,7526 | 0,0272 | 0,7481 | 0,0265 | 0,7553 | 0,0318 | 0,7589 | 0,0252 |
| **L-SOC-CN** | **R-IC** | **RD** | 0,5898 | 0,0266 | 0,5829 | 0,0257 | 0,5767 | 0,0283 | 0,5786 | 0,0244 | 0,5812 | 0,0238 | 0,5834 | 0,0238 | 0,5817 | 0,0227 | 0,5796 | 0,0243 | 0,5849 | 0,0300 | 0,5859 | 0,0273 |
| **L-SOC-CN** | **R-IC** | **AD** | 1,1006 | 0,0425 | 1,0896 | 0,0507 | 1,0840 | 0,0446 | 1,1008 | 0,0500 | 1,0932 | 0,0393 | 1,1019 | 0,0428 | 1,0946 | 0,0455 | 1,0852 | 0,0394 | 1,0961 | 0,0470 | 1,1050 | 0,0366 |
| **R-MGN** | **R-IC** | **MD** | 0,7525 | 0,0318 | 0,7421 | 0,0299 | 0,7437 | 0,0323 | 0,7430 | 0,0269 | 0,7513 | 0,0293 | 0,7470 | 0,0264 | 0,7475 | 0,0275 | 0,7407 | 0,0250 | 0,7481 | 0,0320 | 0,7532 | 0,0263 |
| **R-MGN** | **R-IC** | **RD** | 0,5828 | 0,0345 | 0,5747 | 0,0286 | 0,5783 | 0,0320 | 0,5744 | 0,0263 | 0,5833 | 0,0310 | 0,5778 | 0,0300 | 0,5783 | 0,0258 | 0,5737 | 0,0258 | 0,5801 | 0,0308 | 0,5847 | 0,0263 |
| **R-MGN** | **R-IC** | **AD** | 1,0919 | 0,0344 | 1,0770 | 0,0396 | 1,0747 | 0,0417 | 1,0803 | 0,0345 | 1,0872 | 0,0399 | 1,0854 | 0,0299 | 1,0858 | 0,0402 | 1,0748 | 0,0321 | 1,0840 | 0,0421 | 1,0901 | 0,0315 |
| **R-Caudate** | **R-IC** | **MD** | 0,6970 | 0,0215 | 0,6903 | 0,0245 | 0,6919 | 0,0288 | 0,6972 | 0,0192 | 0,6987 | 0,0181 | 0,6946 | 0,0259 | 0,6969 | 0,0197 | 0,6890 | 0,0237 | 0,6943 | 0,0229 | 0,6983 | 0,0201 |
| **R-Caudate** | **R-IC** | **RD** | 0,5317 | 0,0227 | 0,5253 | 0,0258 | 0,5292 | 0,0295 | 0,5293 | 0,0213 | 0,5349 | 0,0165 | 0,5315 | 0,0256 | 0,5327 | 0,0187 | 0,5239 | 0,0252 | 0,5308 | 0,0217 | 0,5347 | 0,0213 |
| **R-Caudate** | **R-IC** | **AD** | 1,0275 | 0,0284 | 1,0204 | 0,0319 | 1,0174 | 0,0391 | 1,0328 | 0,0263 | 1,0263 | 0,0366 | 1,0208 | 0,0329 | 1,0254 | 0,0371 | 1,0194 | 0,0336 | 1,0214 | 0,0337 | 1,0255 | 0,0306 |
| **R-SOC-CN** | **R-IC** | **MD** | 0,7161 | 0,0200 | 0,7054 | 0,0235 | 0,7058 | 0,0235 | 0,7112 | 0,0176 | 0,7093 | 0,0211 | 0,7115 | 0,0214 | 0,7096 | 0,0200 | 0,7045 | 0,0172 | 0,7090 | 0,0219 | 0,7141 | 0,0212 |
| **R-SOC-CN** | **R-IC** | **RD** | 0,5390 | 0,0203 | 0,5301 | 0,0233 | 0,5314 | 0,0219 | 0,5293 | 0,0207 | 0,5344 | 0,0155 | 0,5339 | 0,0201 | 0,5333 | 0,0180 | 0,5261 | 0,0200 | 0,5342 | 0,0181 | 0,5347 | 0,0213 |
| **R-SOC-CN** | **R-IC** | **AD** | 1,0703 | 0,0442 | 1,0458 | 0,0465 | 1,0439 | 0,0478 | 1,0632 | 0,0489 | 1,0554 | 0,0441 | 1,0547 | 0,0446 | 1,0586 | 0,0386 | 1,0545 | 0,0462 | 1,0587 | 0,0438 | 1,0651 | 0,0489 |
| **R-SPC** | **R-IC** | **MD** | 0,7506 | 0,0258 | 0,7460 | 0,0282 | 0,7516 | 0,0283 | 0,7428 | 0,0232 | 0,7518 | 0,0268 | 0,7466 | 0,0272 | 0,7485 | 0,0224 | 0,7486 | 0,0220 | 0,7527 | 0,0265 | 0,7504 | 0,0243 |
| **R-SPC** | **R-IC** | **RD** | 0,5681 | 0,0243 | 0,5648 | 0,0284 | 0,5684 | 0,0281 | 0,5620 | 0,0244 | 0,5698 | 0,0258 | 0,5627 | 0,0260 | 0,5664 | 0,0233 | 0,5658 | 0,0237 | 0,5691 | 0,0262 | 0,5657 | 0,0267 |
| **R-SPC** | **R-IC** | **AD** | 1,1156 | 0,0362 | 1,1084 | 0,0348 | 1,1179 | 0,0363 | 1,1043 | 0,0294 | 1,1156 | 0,0395 | 1,1143 | 0,0368 | 1,1127 | 0,0322 | 1,1142 | 0,0255 | 1,1199 | 0,0366 | 1,1197 | 0,0303 |
| **R-SFC** | **R-IC** | **MD** | 0,7173 | 0,0235 | 0,7180 | 0,0250 | 0,7186 | 0,0257 | 0,7206 | 0,0238 | 0,7236 | 0,0221 | 0,7180 | 0,0234 | 0,7220 | 0,0200 | 0,7176 | 0,0253 | 0,7223 | 0,0223 | 0,7179 | 0,0251 |
| **R-SFC** | **R-IC** | **RD** | 0,5340 | 0,0229 | 0,5363 | 0,0240 | 0,5359 | 0,0275 | 0,5402 | 0,0241 | 0,5402 | 0,0225 | 0,5354 | 0,0254 | 0,5399 | 0,0220 | 0,5380 | 0,0266 | 0,5361 | 0,0272 | 0,5351 | 0,0234 |
| **R-SFC** | **R-IC** | **AD** | 1,0840 | 0,0398 | 1,0816 | 0,0369 | 1,0840 | 0,0388 | 1,0814 | 0,0309 | 1,0906 | 0,0297 | 1,0832 | 0,0358 | 1,0863 | 0,0258 | 1,0767 | 0,0364 | 1,0946 | 0,0263 | 1,0835 | 0,0388 |
| **R-Medio_Dorsal** | **R-IC** | **MD** | 0,7132 | 0,0411 | 0,7053 | 0,0405 | 0,6990 | 0,0435 | 0,7094 | 0,0449 | 0,7078 | 0,0535 | 0,7123 | 0,0289 | 0,7004 | 0,0486 | 0,7004 | 0,0413 | 0,7149 | 0,0504 | 0,7140 | 0,0505 |
| **R-Medio_Dorsal** | **R-IC** | **RD** | 0,5680 | 0,0370 | 0,5614 | 0,0386 | 0,5568 | 0,0399 | 0,5627 | 0,0399 | 0,5623 | 0,0484 | 0,5695 | 0,0266 | 0,5569 | 0,0435 | 0,5593 | 0,0357 | 0,5705 | 0,0435 | 0,5715 | 0,0450 |
| **R-Medio_Dorsal** | **R-IC** | **AD** | 1,0038 | 0,0629 | 0,9931 | 0,0541 | 0,9832 | 0,0590 | 1,0029 | 0,0664 | 0,9989 | 0,0737 | 0,9980 | 0,0444 | 0,9874 | 0,0709 | 0,9827 | 0,0675 | 1,0037 | 0,0755 | 0,9991 | 0,0713 |
| **R-Pallidum** | **R-IC** | **MD** | 0,6924 | 0,0194 | 0,6887 | 0,0197 | 0,6868 | 0,0215 | 0,6947 | 0,0204 | 0,6873 | 0,0224 | 0,6891 | 0,0174 | 0,6896 | 0,0226 | 0,6885 | 0,0179 | 0,6911 | 0,0179 | 0,6925 | 0,0199 |
| **R-Pallidum** | **R-IC** | **RD** | 0,5138 | 0,0250 | 0,5098 | 0,0220 | 0,5128 | 0,0225 | 0,5193 | 0,0247 | 0,5098 | 0,0184 | 0,5110 | 0,0260 | 0,5149 | 0,0233 | 0,5110 | 0,0257 | 0,5130 | 0,0230 | 0,5177 | 0,0216 |
| **R-Pallidum** | **R-IC** | **AD** | 1,0496 | 0,0427 | 1,0465 | 0,0383 | 1,0346 | 0,0481 | 1,0456 | 0,0308 | 1,0425 | 0,0480 | 1,0453 | 0,0361 | 1,0390 | 0,0450 | 1,0433 | 0,0393 | 1,0473 | 0,0441 | 1,0421 | 0,0409 |
| **R-lOFC** | **R-PAC** | **MD** | 0,7801 | 0,0181 | 0,7835 | 0,0221 | 0,7843 | 0,0244 | 0,7781 | 0,0165 | 0,7847 | 0,0255 | 0,7783 | 0,0204 | 0,7810 | 0,0218 | 0,7825 | 0,0169 | 0,7784 | 0,0236 | 0,7848 | 0,0228 |
| **R-lOFC** | **R-PAC** | **RD** | 0,5890 | 0,0225 | 0,5901 | 0,0261 | 0,5945 | 0,0265 | 0,5854 | 0,0198 | 0,5944 | 0,0257 | 0,5899 | 0,0253 | 0,5895 | 0,0231 | 0,5888 | 0,0201 | 0,5877 | 0,0252 | 0,5922 | 0,0265 |
| **R-lOFC** | **R-PAC** | **AD** | 1,1624 | 0,0314 | 1,1702 | 0,0237 | 1,1639 | 0,0340 | 1,1634 | 0,0259 | 1,1653 | 0,0381 | 1,1551 | 0,0300 | 1,1639 | 0,0306 | 1,1699 | 0,0259 | 1,1599 | 0,0335 | 1,1699 | 0,0301 |
| **R-pars** | **R-PAC** | **MD** | 0,7795 | 0,0218 | 0,7806 | 0,0246 | 0,7829 | 0,0252 | 0,7748 | 0,0177 | 0,7846 | 0,0267 | 0,7779 | 0,0236 | 0,7799 | 0,0230 | 0,7809 | 0,0180 | 0,7786 | 0,0263 | 0,7822 | 0,0245 |
| **R-pars** | **R-PAC** | **RD** | 0,5871 | 0,0242 | 0,5870 | 0,0289 | 0,5917 | 0,0278 | 0,5835 | 0,0224 | 0,5946 | 0,0288 | 0,5868 | 0,0263 | 0,5897 | 0,0254 | 0,5886 | 0,0198 | 0,5898 | 0,0269 | 0,5917 | 0,0294 |
| **R-pars** | **R-PAC** | **AD** | 1,1644 | 0,0353 | 1,1678 | 0,0329 | 1,1653 | 0,0348 | 1,1573 | 0,0235 | 1,1646 | 0,0371 | 1,1603 | 0,0349 | 1,1603 | 0,0330 | 1,1655 | 0,0331 | 1,1564 | 0,0384 | 1,1630 | 0,0306 |
| **R-parstr** | **R-PAC** | **MD** | 0,7720 | 0,0249 | 0,7733 | 0,0272 | 0,7763 | 0,0276 | 0,7670 | 0,0197 | 0,7779 | 0,0284 | 0,7705 | 0,0274 | 0,7720 | 0,0256 | 0,7729 | 0,0225 | 0,7744 | 0,0281 | 0,7719 | 0,0256 |
| **R-parstr** | **R-PAC** | **RD** | 0,5863 | 0,0244 | 0,5893 | 0,0268 | 0,5914 | 0,0280 | 0,5819 | 0,0243 | 0,5939 | 0,0289 | 0,5870 | 0,0253 | 0,5871 | 0,0255 | 0,5880 | 0,0213 | 0,5879 | 0,0291 | 0,5882 | 0,0316 |
| **R-parstr** | **R-PAC** | **AD** | 1,1433 | 0,0415 | 1,1414 | 0,0429 | 1,1462 | 0,0416 | 1,1371 | 0,0217 | 1,1461 | 0,0450 | 1,1376 | 0,0447 | 1,1419 | 0,0419 | 1,1427 | 0,0414 | 1,1472 | 0,0428 | 1,1392 | 0,0230 |
| **R-SOC-CN** | **R-PAC** | **MD** | 0,7309 | 0,0400 | 0,7209 | 0,0406 | 0,7256 | 0,0439 | 0,7309 | 0,0330 | 0,7257 | 0,0321 | 0,7254 | 0,0331 | 0,7154 | 0,0299 | 0,7251 | 0,0396 | 0,7266 | 0,0338 | 0,7225 | 0,0358 |
| **R-SOC-CN** | **R-PAC** | **RD** | 0,5443 | 0,0317 | 0,5355 | 0,0351 | 0,5419 | 0,0382 | 0,5387 | 0,0291 | 0,5473 | 0,0273 | 0,5455 | 0,0331 | 0,5353 | 0,0232 | 0,5381 | 0,0325 | 0,5457 | 0,0284 | 0,5409 | 0,0333 |
| **R-SOC-CN** | **R-PAC** | **AD** | 1,1005 | 0,0611 | 1,0829 | 0,0537 | 1,0896 | 0,0633 | 1,1062 | 0,0503 | 1,0883 | 0,0601 | 1,0851 | 0,0487 | 1,0813 | 0,0596 | 1,0900 | 0,0563 | 1,0935 | 0,0633 | 1,0855 | 0,0531 |
| **R-Caudate** | **R-PAC** | **MD** | 0,7805 | 0,0273 | 0,7823 | 0,0336 | 0,7859 | 0,0332 | 0,7825 | 0,0254 | 0,7900 | 0,0277 | 0,7851 | 0,0312 | 0,7879 | 0,0309 | 0,7809 | 0,0221 | 0,7834 | 0,0285 | 0,7863 | 0,0277 |
| **R-Caudate** | **R-PAC** | **RD** | 0,5934 | 0,0270 | 0,5942 | 0,0361 | 0,5977 | 0,0326 | 0,5942 | 0,0274 | 0,6035 | 0,0276 | 0,5965 | 0,0295 | 0,6005 | 0,0313 | 0,5947 | 0,0235 | 0,5991 | 0,0298 | 0,6014 | 0,0300 |
| **R-Caudate** | **R-PAC** | **AD** | 1,1548 | 0,0483 | 1,1584 | 0,0467 | 1,1623 | 0,0462 | 1,1591 | 0,0370 | 1,1630 | 0,0496 | 1,1623 | 0,0526 | 1,1626 | 0,0432 | 1,1534 | 0,0355 | 1,1518 | 0,0483 | 1,1561 | 0,0374 |
| **R-MGN** | **R-PAC** | **MD** | 0,7221 | 0,0130 | 0,7188 | 0,0171 | 0,7208 | 0,0151 | 0,7257 | 0,0151 | 0,7278 | 0,0097 | 0,7234 | 0,0130 | 0,7236 | 0,0137 | 0,7182 | 0,0138 | 0,7230 | 0,0124 | 0,7237 | 0,0137 |
| **R-MGN** | **R-PAC** | **RD** | 0,5230 | 0,0181 | 0,5159 | 0,0210 | 0,5188 | 0,0212 | 0,5233 | 0,0228 | 0,5240 | 0,0181 | 0,5186 | 0,0185 | 0,5190 | 0,0188 | 0,5193 | 0,0184 | 0,5204 | 0,0167 | 0,5256 | 0,0158 |
| **R-MGN** | **R-PAC** | **AD** | 1,1349 | 0,0214 | 1,1272 | 0,0273 | 1,1254 | 0,0256 | 1,1292 | 0,0227 | 1,1326 | 0,0240 | 1,1296 | 0,0217 | 1,1260 | 0,0232 | 1,1256 | 0,0212 | 1,1316 | 0,0230 | 1,1327 | 0,0240 |
| **R-IC** | **R-PAC** | **MD** | 0,7368 | 0,0201 | 0,7321 | 0,0214 | 0,7299 | 0,0216 | 0,7360 | 0,0235 | 0,7393 | 0,0180 | 0,7330 | 0,0169 | 0,7357 | 0,0183 | 0,7346 | 0,0167 | 0,7332 | 0,0203 | 0,7412 | 0,0183 |
| **R-IC** | **R-PAC** | **RD** | 0,5438 | 0,0173 | 0,5378 | 0,0204 | 0,5384 | 0,0188 | 0,5435 | 0,0234 | 0,5441 | 0,0176 | 0,5385 | 0,0207 | 0,5418 | 0,0181 | 0,5432 | 0,0166 | 0,5379 | 0,0158 | 0,5453 | 0,0179 |
| **R-IC** | **R-PAC** | **AD** | 1,1228 | 0,0357 | 1,1205 | 0,0361 | 1,1128 | 0,0389 | 1,1209 | 0,0385 | 1,1298 | 0,0318 | 1,1218 | 0,0246 | 1,1235 | 0,0263 | 1,1174 | 0,0253 | 1,1237 | 0,0375 | 1,1330 | 0,0292 |
| **R-Pallidum** | **R-PAC** | **MD** | 0,7462 | 0,0250 | 0,7468 | 0,0249 | 0,7448 | 0,0273 | 0,7477 | 0,0234 | 0,7539 | 0,0236 | 0,7438 | 0,0232 | 0,7475 | 0,0238 | 0,7430 | 0,0204 | 0,7483 | 0,0234 | 0,7461 | 0,0280 |
| **R-Pallidum** | **R-PAC** | **RD** | 0,5409 | 0,0283 | 0,5419 | 0,0285 | 0,5392 | 0,0321 | 0,5440 | 0,0332 | 0,5512 | 0,0281 | 0,5401 | 0,0263 | 0,5432 | 0,0289 | 0,5419 | 0,0278 | 0,5411 | 0,0295 | 0,5418 | 0,0355 |
| **R-Pallidum** | **R-PAC** | **AD** | 1,1528 | 0,0364 | 1,1525 | 0,0329 | 1,1519 | 0,0332 | 1,1549 | 0,0296 | 1,1556 | 0,0361 | 1,1468 | 0,0292 | 1,1521 | 0,0311 | 1,1406 | 0,0226 | 1,1588 | 0,0328 | 1,1547 | 0,0330 |
| **R-SPC** | **R-PAC** | **MD** | 0,7838 | 0,0255 | 0,7802 | 0,0280 | 0,7831 | 0,0295 | 0,7759 | 0,0242 | 0,7843 | 0,0302 | 0,7792 | 0,0258 | 0,7827 | 0,0278 | 0,7847 | 0,0238 | 0,7818 | 0,0272 | 0,7850 | 0,0238 |
| **R-SPC** | **R-PAC** | **RD** | 0,5901 | 0,0284 | 0,5925 | 0,0262 | 0,5950 | 0,0296 | 0,5877 | 0,0235 | 0,5922 | 0,0304 | 0,5901 | 0,0246 | 0,5924 | 0,0280 | 0,5938 | 0,0232 | 0,5887 | 0,0290 | 0,5911 | 0,0277 |
| **R-SPC** | **R-PAC** | **AD** | 1,1711 | 0,0325 | 1,1556 | 0,0433 | 1,1594 | 0,0396 | 1,1522 | 0,0361 | 1,1684 | 0,0391 | 1,1574 | 0,0380 | 1,1634 | 0,0373 | 1,1665 | 0,0335 | 1,1680 | 0,0350 | 1,1729 | 0,0280 |
| **R-ITC** | **R-PAC** | **MD** | 0,7986 | 0,0252 | 0,7969 | 0,0263 | 0,8006 | 0,0267 | 0,7914 | 0,0218 | 0,7992 | 0,0264 | 0,7967 | 0,0242 | 0,7985 | 0,0252 | 0,7965 | 0,0229 | 0,7992 | 0,0269 | 0,8001 | 0,0258 |
| **R-ITC** | **R-PAC** | **RD** | 0,6067 | 0,0278 | 0,6060 | 0,0262 | 0,6093 | 0,0281 | 0,6013 | 0,0270 | 0,6086 | 0,0291 | 0,6057 | 0,0237 | 0,6084 | 0,0287 | 0,6072 | 0,0271 | 0,6069 | 0,0312 | 0,6067 | 0,0300 |
| **R-ITC** | **R-PAC** | **AD** | 1,1825 | 0,0272 | 1,1787 | 0,0351 | 1,1831 | 0,0337 | 1,1716 | 0,0229 | 1,1803 | 0,0336 | 1,1787 | 0,0341 | 1,1786 | 0,0292 | 1,1751 | 0,0224 | 1,1840 | 0,0298 | 1,1871 | 0,0290 |
| **R-Amygdala** | **R-PAC** | **MD** | 0,7854 | 0,0217 | 0,7847 | 0,0256 | 0,7859 | 0,0260 | 0,7835 | 0,0215 | 0,7919 | 0,0237 | 0,7811 | 0,0220 | 0,7859 | 0,0220 | 0,7835 | 0,0196 | 0,7896 | 0,0247 | 0,7872 | 0,0253 |
| **R-Amygdala** | **R-PAC** | **RD** | 0,5847 | 0,0219 | 0,5830 | 0,0263 | 0,5873 | 0,0278 | 0,5824 | 0,0263 | 0,5921 | 0,0261 | 0,5807 | 0,0232 | 0,5845 | 0,0274 | 0,5853 | 0,0233 | 0,5856 | 0,0282 | 0,5843 | 0,0290 |
| **R-Amygdala** | **R-PAC** | **AD** | 1,1869 | 0,0375 | 1,1883 | 0,0386 | 1,1831 | 0,0379 | 1,1857 | 0,0267 | 1,1916 | 0,0354 | 1,1817 | 0,0339 | 1,1887 | 0,0299 | 1,1800 | 0,0295 | 1,1975 | 0,0305 | 1,1928 | 0,0339 |
| **R-Putamen** | **R-PAC** | **MD** | 0,7644 | 0,0228 | 0,7630 | 0,0234 | 0,7662 | 0,0248 | 0,7618 | 0,0208 | 0,7702 | 0,0257 | 0,7610 | 0,0211 | 0,7662 | 0,0245 | 0,7632 | 0,0221 | 0,7688 | 0,0236 | 0,7637 | 0,0249 |
| **R-Putamen** | **R-PAC** | **RD** | 0,5747 | 0,0224 | 0,5755 | 0,0244 | 0,5775 | 0,0273 | 0,5747 | 0,0229 | 0,5851 | 0,0243 | 0,5756 | 0,0213 | 0,5810 | 0,0236 | 0,5781 | 0,0236 | 0,5802 | 0,0244 | 0,5763 | 0,0285 |
| **R-Putamen** | **R-PAC** | **AD** | 1,1438 | 0,0359 | 1,1381 | 0,0309 | 1,1437 | 0,0329 | 1,1360 | 0,0267 | 1,1405 | 0,0387 | 1,1318 | 0,0293 | 1,1365 | 0,0352 | 1,1334 | 0,0256 | 1,1460 | 0,0330 | 1,1385 | 0,0293 |
| **R-LOC** | **R-PAC** | **MD** | 0,7951 | 0,0349 | 0,7941 | 0,0333 | 0,7953 | 0,0360 | 0,7826 | 0,0202 | 0,7942 | 0,0350 | 0,7905 | 0,0331 | 0,7950 | 0,0319 | 0,7938 | 0,0315 | 0,7945 | 0,0339 | 0,7912 | 0,0241 |
| **R-LOC** | **R-PAC** | **RD** | 0,5835 | 0,0382 | 0,5857 | 0,0357 | 0,5877 | 0,0393 | 0,5718 | 0,0299 | 0,5838 | 0,0361 | 0,5820 | 0,0357 | 0,5876 | 0,0315 | 0,5844 | 0,0337 | 0,5877 | 0,0345 | 0,5823 | 0,0305 |
| **R-LOC** | **R-PAC** | **AD** | 1,2182 | 0,0370 | 1,2110 | 0,0395 | 1,2104 | 0,0392 | 1,2041 | 0,0272 | 1,2151 | 0,0407 | 1,2074 | 0,0375 | 1,2099 | 0,0428 | 1,2125 | 0,0403 | 1,2082 | 0,0396 | 1,2091 | 0,0269 |
| **R-Anterior** | **R-PAC** | **MD** | 0,7793 | 0,0210 | 0,7811 | 0,0251 | 0,7772 | 0,0251 | 0,7724 | 0,0189 | 0,7838 | 0,0222 | 0,7752 | 0,0217 | 0,7799 | 0,0217 | 0,7797 | 0,0147 | 0,7820 | 0,0248 | 0,7818 | 0,0248 |
| **R-Anterior** | **R-PAC** | **RD** | 0,5930 | 0,0210 | 0,5901 | 0,0297 | 0,5913 | 0,0254 | 0,5840 | 0,0256 | 0,5970 | 0,0240 | 0,5874 | 0,0274 | 0,5919 | 0,0250 | 0,5932 | 0,0193 | 0,5942 | 0,0250 | 0,5947 | 0,0265 |
| **R-Anterior** | **R-PAC** | **AD** | 1,1519 | 0,0387 | 1,1631 | 0,0343 | 1,1491 | 0,0395 | 1,1490 | 0,0257 | 1,1574 | 0,0406 | 1,1508 | 0,0351 | 1,1557 | 0,0309 | 1,1525 | 0,0304 | 1,1576 | 0,0416 | 1,1560 | 0,0337 |
| **R-IPC** | **R-PAC** | **MD** | 0,7975 | 0,0258 | 0,7967 | 0,0306 | 0,8008 | 0,0306 | 0,7923 | 0,0271 | 0,8014 | 0,0293 | 0,7964 | 0,0264 | 0,7990 | 0,0284 | 0,7988 | 0,0268 | 0,8008 | 0,0285 | 0,8018 | 0,0271 |
| **R-IPC** | **R-PAC** | **RD** | 0,6106 | 0,0258 | 0,6128 | 0,0287 | 0,6163 | 0,0295 | 0,6081 | 0,0272 | 0,6154 | 0,0288 | 0,6121 | 0,0261 | 0,6144 | 0,0283 | 0,6144 | 0,0279 | 0,6139 | 0,0286 | 0,6138 | 0,0287 |
| **R-IPC** | **R-PAC** | **AD** | 1,1714 | 0,0323 | 1,1646 | 0,0391 | 1,1696 | 0,0376 | 1,1608 | 0,0313 | 1,1735 | 0,0371 | 1,1651 | 0,0317 | 1,1680 | 0,0329 | 1,1677 | 0,0323 | 1,1744 | 0,0338 | 1,1777 | 0,0323 |
| **R-TP** | **R-PAC** | **MD** | 0,8172 | 0,0297 | 0,8119 | 0,0331 | 0,8187 | 0,0306 | 0,8113 | 0,0264 | 0,8190 | 0,0317 | 0,8164 | 0,0312 | 0,8171 | 0,0322 | 0,8130 | 0,0293 | 0,8227 | 0,0312 | 0,8197 | 0,0270 |
| **R-TP** | **R-PAC** | **RD** | 0,6281 | 0,0297 | 0,6212 | 0,0350 | 0,6318 | 0,0295 | 0,6231 | 0,0308 | 0,6295 | 0,0322 | 0,6265 | 0,0336 | 0,6246 | 0,0336 | 0,6243 | 0,0320 | 0,6319 | 0,0330 | 0,6300 | 0,0335 |
| **R-TP** | **R-PAC** | **AD** | 1,1954 | 0,0394 | 1,1932 | 0,0406 | 1,1926 | 0,0416 | 1,1878 | 0,0274 | 1,1980 | 0,0400 | 1,1961 | 0,0382 | 1,2021 | 0,0386 | 1,1903 | 0,0372 | 1,2043 | 0,0368 | 1,1990 | 0,0254 |
| **R-fusiform** | **R-PAC** | **MD** | 0,7971 | 0,0261 | 0,7954 | 0,0265 | 0,8000 | 0,0258 | 0,7896 | 0,0221 | 0,7992 | 0,0265 | 0,7937 | 0,0229 | 0,7975 | 0,0248 | 0,7951 | 0,0233 | 0,7976 | 0,0247 | 0,7977 | 0,0216 |
| **R-fusiform** | **R-PAC** | **RD** | 0,5956 | 0,0252 | 0,6021 | 0,0240 | 0,6030 | 0,0276 | 0,5903 | 0,0264 | 0,6009 | 0,0253 | 0,5998 | 0,0211 | 0,6005 | 0,0235 | 0,5988 | 0,0256 | 0,5955 | 0,0266 | 0,5988 | 0,0249 |
| **R-fusiform** | **R-PAC** | **AD** | 1,2003 | 0,0369 | 1,1820 | 0,0416 | 1,1940 | 0,0394 | 1,1881 | 0,0309 | 1,1956 | 0,0439 | 1,1817 | 0,0392 | 1,1915 | 0,0426 | 1,1877 | 0,0356 | 1,2019 | 0,0363 | 1,1955 | 0,0316 |
